# Discovery of potent anti-toxoplasmosis drugs from secondary metabolites in *Citrus limon* (lemon) leaves, supported in-silico study

**DOI:** 10.1038/s41598-024-82787-9

**Published:** 2025-01-03

**Authors:** Magdy Mostafa Desoky Mohammed, Hala Sh. Mohammed, Salwa A. Abu El Wafa, Doaa A. Ahmed, Elham A. Heikal, Islam Elgohary, Ashraf M. Barakat

**Affiliations:** 1 Pharmacognosy Department, Pharmaceutical and Drug Industries Research Institute, National Research Centre, Dokki, Giza, 12622 Egypt; 2 Pharmacognosy and Medicinal Plants Department, Faculty of Pharmacy (Girls), Al-Azhar University, Cairo, Egypt; 3 Medical Parasitology Department, Faculty of Medicine, Al-Azhar University for Girls, Cairo, Egypt; 4 Department of Pathology, Agriculture Research Centre, Animal Health Research Institute, Dokki, Giza, Egypt; 5 Department of Zoonotic Diseases, National Research Centre, Dokki, Giza, 12622 Egypt

**Keywords:** *Citrus limon*, Rutaceae, *Toxoplasma Gondii*, UPLC-ESI-MS/MS, Molecular docking, Molecular dynamics simulation, Biochemistry, Drug discovery, Chemistry, Medicinal chemistry, Organic chemistry

## Abstract

Toxoplasmosis induced by *Toxoplasma gondii* is a well-known health threat, that prompts fatal encephalitis increased with immunocompromised patients, in addition, it can cause chorioretinitis, microcephaly, stillbirth in the fetus and even led to death. Standard therapy uses sulfadiazine and pyrimethamine drugs revealed beneficial results during the acute stage, however, it has severe side effects. UPLC-ESI-MS/MS used to explore *C. limon* MeOH ext. constituents, which revealed a list of **41** metabolites of different classes encompasses; unsaturated fatty acid, tricarboxylic acids, phenolic aldehyde, phenolic acids, phenolic glycosides, coumarins, sesquiterpene lactone, limonoid, steroid and flavonoids. *C. limon* MeOH ext. and the isolates reduced significantly the number of *T. gondii* tachyzoites. Consequently, histopathological examination, proved significant reduction in the number of mononuclear inflammatory cells in the kidney and liver sections, besides, lowering the number of shrunken and degenerative neurons in the brain sections of infected mice. Molecular docking study was performed targeted certain receptors, which are important for the life cycle fundamentals for the parasite mobility including invasion and egress, and further molecular dynamics simulation was conducted to get insights into the structural changes of the formed complexes, along with a pharmacophoric mapping approach, that confirmed the need for a free hydroxyl group and/or a phenolic substituted one, in order to form HB, Hyd/Aro and ML interactions, through which, cell cycle disruption via iron chelation, could be achieved. In addition, the ADMIT properties of all identified metabolites were predicted.

**Supplementary Information:**

The online version contains supplementary material available at 10.1038/s41598-024-82787-9.

## Introduction

Toxoplasmosis is a zoonotic infection with a parasite named *Toxoplasma gondii*, it affects the world’s population by one-third, and imposes a major public health problem, particularly in certain populations and regions, with pooled prevalence of *T. gondii* infection in Africa (21.74–74.8%), Asia (13.3–85.3%), Europe (40–76%) and North America (7.3–26.5%)^1^. However, Worldwide estimates revealed that congenital toxoplasmosis results in about 190,000 cases annually^2^. The collective frequency of chronic and acute *T. gondii* infection among neuropsychiatric patients was 38.27% and 6.78%, respectively^2,3^.

*T. gondii* has got wide range of hosts, with very high ubiquity rate in humans; pregnant women, immunocompromised patients (frequency of cancer and AIDS) and warm-blooded animals (increased pet ownership). Usually, *T. gondii* transferred by drinking water (contaminated with oocysts from a feline source), ingesting milk and food products (contaminated with infective oocysts), and by congenital and venereal transmission. Hence, toxoplasmosis is more common to be distributed in rural areas, based on the high contact between humans and animals such as cattle, goats and sheep, or by eating contaminated fruits and vegetables^4^.

Ideal anti-toxoplasmosis drugs should be safe for the host with powerful parasiticidal potential against the parasitic life cycle at different stages. However, the currently used drugs failed to achieve such criteria. Notwithstanding, the tragic consequences of toxoplasmosis, the standard therapy uses sulfadiazine and pyrimethamine drugs revealed beneficial results during the acute stage, although, it has severe side effects including allergy, kidney stones and hepatic or renal complications, even more, pyrimethamine can cause bone marrow suppression and folate depletion, thus, it cannot be used during the first trimester of pregnancy. Moreover, drugs like atovaquone, spiramicyne, and clindamycine are used in chronic conditions, but with limited efficacy due to their toxicity to the fetus and to the emergence of resistant strains. However, sulfadiazine (sulfonamide) along with pyrimethamine (dihydrofolate reductase ‘DHFR’ inhibitor) have significantly lower parasiticidal activity^5^. Recently, atovaquone, epiroprim (DHFR inhibitors) and fluoroquinolone antibiotics are effective in vitro and in vivo against *T. gondii*, but their usage in pregnancy is limited as their safety has not been established^6^.

The aforementioned drugs, even with its risk, used to reduce and/or drive out the symptoms of toxoplasmosis, with no clear activity on the parasite and/or to cure the infected host. Therefore, the development of more viable treatment options including new safe and affordable drugs to eradicate toxoplasmosis are of paramount importance. Recently, an endless number of medicinal herbs from different origins have been investigated against parasitic diseases (i.e., anti-toxoplasmosis), and resulted with satisfactory outcomes, helping the pharmaceutical industry to develop anti-parasitic drugs from natural origin^7–9^.

*Citrus limon* (L.) a medicinal plant belongs to the family Rutaceae, it is an evergreen tree with yellow fruits, and prevalent in Mediterranean regions of warm and/or mild climate^10^. It has been used by traditional healers in Eastern Tanzania for the treatment of schistosomiasis^11^, and have been reported by others for the treatment of malaria, measles, dysentery, etc^12^. *C. limon* is a very rich source with bioactive metabolites including; coumarin, flavonoids, phenolic acids, limonoids, carotenoids, etc., spread over the fruits, juice and leaves^13,14^. The thing that reflects its medicinal potential against a variety of ailments such as airway diseases (i.e. asthma, cough and tuberculosis), antibiotics, antiseptic, antiviral, cancer, cardiovascular diseases, diabetes and obesity^15^. Moreover, it showed antibacterial and antileishmanial activities^16^. In addition, *C. sinensis* seed oil was reported to possess anti-*toxoplasma gondii* and antimicrobial potentials^17^.

Noteworthy that, the acute and subacute toxicity of *C. limon* juice on Sprawgue Dawley rats were reported by Oyebadejo and Solomon^18^, who concluded that, with LD50 value (86.60%) of *C. limon* juice, non-clinical signs of toxicity, no ataxia and sign of mortality were recorded, moreover, the activated partial thromboplastin, prothrombin, clothing time, renal and liver function profile were not significantly changed.

Furthermore, a prospective in vitro/in vivo study was designed by Riaz et al., reported that^19^, *C. limon* juice increased significantly the thrombin time with partial activation of the thromboplastin time, whereas, fibrinogen concentration was significantly reduced. Moreover, in vivo results revealed significant changes in the hematological parameters i.e., hemoglobin, erythrocytes and mean corpuscular hemoglobin concentration. Along with, significant prolongation in the bleeding and thrombin time together with increase in protein C and thrombin antithrombin complex levels. Additionally, Aboelhadid and co-workers^20^ reported the in vitro and in vivo effect of *C. limon* oil on *Sarcoptes scabiei* var. *cuniculi* infected rabbits, and proved that, mites treated in vitro with 20% of the oil recorded significant increase in hydrogen peroxide and malondialdehyde. Whereas, complete recovery was achieved upon in vivo application of 20% *C. limon* oil, and resulted in complete absence of mite in microscopic examination, significant improvement in productivity, stoppage of scale formation in treated tissues and faster hair growth than deltamethrin treated rabbits. Additionally, Bonkian and others^21^ studied the in vivo antiplasmodial and insecticidal activities of *C. limon* leaves extracts, and concluded that, the acetone ext. considerably reduced mice parasitemia, while, the hexane ext. displayed poor insecticidal activity against *Anopheles gambiae* mosquitoes. Finally, Bekkouch et al. investigated the phytoconstituents and studied the anti-inflammatory potential of *Zingiber officinale* Roscoe and *C. limon* L. juices^22^, and clearly observed that, a suggestive anti-inflammatory protection against in vivo development of the rat paw edema was attained upon treatment with each juice, wherefore, enhanced with their formulation due to vascular permeability inhibition.

Based on what is previously mentioned, our present study was conducted to characterize the chemical constituents of *C. limon* MeOH leaf extract and its isolates, and to assess their efficacy against chronic toxoplasmosis, which was supported by an in-silico investigation to stand on their mechanism of action.

## Materials and methods

All methods were carried out in accordance with relevant guidelines.

### Plant collection and identification

*C. limon* (L.) leaves were collected from Ash Sharqia Governorate, Egypt (31.11660 N and 30.63333 E) in October 2019. The plant material was collected in accordance with applicable institutional, national and international rules and regulations, then it was air-dried and powdered to afford 1 kg fine powder. The authorization of the collected leaf specimens was delegated to Mrs. Therese Labib, the consultant of taxonomy at the ministry of agriculture and the former director of El-Orman Botanical Garden, Giza, Egypt. A voucher specimen (No. Cl-19) was kept at the Herbarium of the Pharmacognosy and Medicinal Plants Department, Faculty of Pharmacy (Girls), Al-Azhar University, Cairo, Egypt.

### Extraction and chromatographic isolation

Powdered air-dried leaves of *C. limon* was exhaustively extracted at room temperature using 70% aqueous alcoholic (4 × 2 L) by percolation. The aqueous alcoholic extracts were concentrated under vacuum at 60 °C to obtain a brown sticky residue (200 g), which was defatted by pet. ether (3 × 500 mL) to afford 27 g and was discarded.

Around of 135 g of the residual (170 g) were loaded on a polyamide column (Reidel De Haen AG, Seeize, Hannover), and eluted with 100% H_2_O followed by H_2_O/MeOH mixtures step-gradient up to 100% MeOH. Similar fractions were grouped into five major fractions (**F**_**A−E**_) according to their PC and TLC profiles. **F**_**A**_ (70 g) contained mainly sugars and inorganic salts with low content of polyphenols and then it was discarded. In addition, **F**_**B**_ (24 g) was fractionated over Sephadex LH-20 column using BIW (Butanol–Isopropyl alcohol–Water; 4:1:5 v/v/v/ upper layer) as an eluent, then identical sub-fractions were gathered together. The primary sub-fraction was then subjected to a successive column chromatography using various solvent systems to afford compound-C1 (0.4 g). Furthermore, **F**_**C**_ (11 g) was subjected to UPLC-ESI-MS/MS analysis, which tentatively led to the identification of diverse metabolites (Table 1). Moreover, **F**_**D&E**_ (20 and 15 g, respectively), were collected based on their chromatographic profile, and fractionated using Sephadex LH-20 with MeOH as an eluent, then, similar sub-fractions were collected according to their PC pattern into two major sub-fractions, which were further purified over Sephadex LH-20 using 100% MeOH to give compounds-C2 and -C3 (0.5 and 0.6 g) respectively.

### Experimental procedures

NMR analysis was conducted using Bruker spectrometer (Bruker Corp., Billerica, MA, USA) operating at (400 MHz for^1^H and 100 MHz for^13^C). All samples were prepared in DMSO-*d*_*6*_ with TMS as internal reference, chemical shifts were expressed in ppm, and coupling constants (*J*) in Hz. (The analysis was done at the NMR unit, the faculty of pharmacy, Cairo University, Egypt). Column chromatography was performed (silica gel 60, 0.060–0.200 mm, 70–230 mesh). To check the purity of the isolated compounds, Sephadex LH-20 and precoated TLC silica gel 60G F_254_ sheets (Sigma Aldrich, Germany) were used. Detection was accomplished by visualizing with a UV lamp at 254 and 365 nm, followed by, spraying with a 10% EtOH/H_2_SO_4_ (v/v) and AlCl_3_ solutions on TLC and PC respectively, and then heating at 120 °C and 5% alc.

### UPLC-ESI-MS/MS conditions

UPLC-ESI-MS/MS analyses (−ve modes) were performed on the UPLC system (Waters Acquity BEH, MA-01757, USA) linked to an electrospray mass spectrometer. A Waters triple-quadrupole mass spectrometer was employed for the analysis (Waters Acquity, XEVO, TQD, MA, USA). The LC effluent was introduced into the mass spectrometer through an electrospray capillary set to + 3.00 kV and a source temperature of 150 °C. At flow rates of 50 and 900 L/h, respectively, a desolvation temperature of 440 °C, and nitrogen was utilized as the cone and desolvation gas. 0.5 µL of the extract was injected on a C_18_ (50 × 2.1 mm, 1.7 μm particle size) column (post-filtering with a 0.2 mm filter membrane disc and degassed by sonication before injection ‘Waters’). Gradient elution was accomplished using the solvents (A) 0.1% formic acid in water (v/v), and (B) 0.1% formic acid in acetonitrile at a flow rate of 0.2 mL/min, with the linear gradient program providing the best sensitivity. For the structural identifications of phenolic compounds, mass spectra were detected in the ESI between *m/z* 100–1000, peaks and spectra were processed using the MassLynx 4.1 software, and tentatively identified by comparing its retention time (R_*t*_) and mass spectral fragmentations with the reported data from the literature and Wiley natural product library.

### Multiple reaction monitoring (MRM) condition

Sample analysis was performed using liquid chromatography electrospray ionization tandem mass spectrometry (LC-ESI-MS/MS) with Exion LC AC system for separation and SCIEX Triple Quad 5500 + MS/MS system equipped with an electrospray ionization (ESI) for detection. The separation was performed using ZORBAX SB-C_18_ Column (4.6 × 100 mm, 1.8 μm). Mobile phases consisted of two eluents A: 0.1% formic acid in water; B: acetonitrile (LC grade), and programmed as follow, 2% B (0–1 min), 2–60% B (1–21 min), 60% B (21–25 min), 2% B (25–28 min), the flow rate was 0.8 ml/min, and the injection volume was 3 μl. For MRM analysis of the selected polyphenols, positive and negative ionization modes were applied in the same run with the following parameters; curtain gas of 25 psi, ion Spray voltage of 4500 and − 4500 for (+ ve & − ve) modes respectively, source temperature of 400 °C, ion source gas 1 and 2 were 55 psi with a declustering potential of 50, collision energy of 25, and collision energy spread of 10.

### In silico molecular docking study

**Fig. 1:**
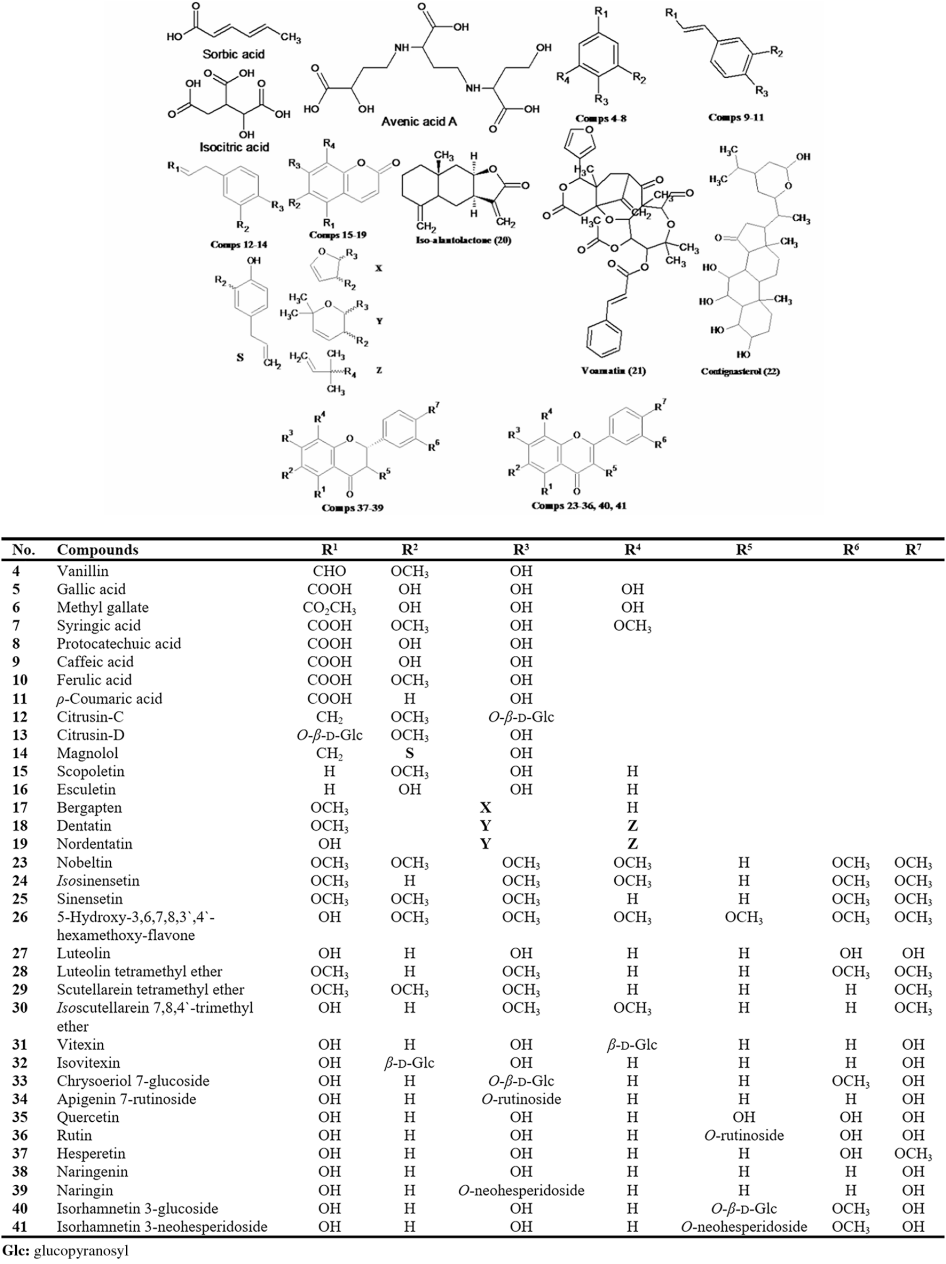
The identified metabolites from *C. limon* leaves MeOH ext.

Molecular docking study including minimization, protonation, etc. was performed as reported^9^. The X-ray crystallographic structure of protein targets (PDB IDs: *Tg*ROP18 (4JRN), *Tg*ROP5B (3Q5Z), *Tg*CDPK1 (4M84), UPRTase (1UPF), AK (1LIJ & 3MB8), *Ts*-DHFR (4KY4), ENR (2O2S), tRNA (6A88), *Tg*-MMIF (4DH4)) were downloaded from RCSB protein data bank (https://www.rcsb.org/). The chemical structures of all identified compounds (Fig. 1) were built using ACD/ChemSketch software, and then were converted into their canonical simplified molecular-input line-entry system (SMILE), then applied for energy minimization using the Merck molecular force field 94 (MMFF94x).

Validation of the docking process was done by re-docking the co-crystalized ligand with its receptor protein, moreover, RMS (Root Mean Square) distance with MMFF94X force-field and the partial charges were automatically calculated. The co-crystalized ligand with every receptor (S_1) was used to define the active site for docking, i.e., binding pocket, ligand interactions, and all amino acids at the active site were established, recorded and isolated^23^, alongside the grid box parameters (S_1). The top scored poses of each cluster were considered for further calculations, with only the best pose, is the one closest to the crystal structure that can be professed with sufficient certainty to occur in nature.

### Molecular dynamics (MD) simulation

A high-throughput dynamics simulation method must be built to study the ligand-target receptor binding process during differentiation, in order to better understanding the molecular mechanism of the docked ligand-target complex interactions, enhance docking findings, explore stability, and to achieve the optimal configuration.

To investigate the dynamic behavior and stability of the best-scored protein–ligand complexes, a conventional MD simulation was conducted using Nanoscale Molecular Dynamics (NAMD) simulation software (NAMD_3.0.1, 2024) for Windows Subsystem Linux-Ubuntu^24^. Simulation preparation including protein PSF builder, complex solvation and ionization, and the analysis of MD simulation results were integrated into the visualization package VMD, while CHARMM-GUI web-based platform, that provides input generator, was used to produce the ligand topology files utilizing the NAMD function input generator^25,26^, then VMD was used for the generation of protein-ligand complexes PSF and PDB files, solvation and finally ionization. The system was solvated by adding water molecules in a water box having a water layer of 11 Å thickness between the boundaries of the box and the minimum/maximum protein coordinates, then NaCl of conc. 0.15 Mol/L counter ions were added to neutralize the system with periodic boundary condition.

To start the conventional MD simulations, in the first stage, the system (solvated and neutralized) was subjected to energy minimization for 10,000 time-steps, at temperature (300 K) with constant pressure using conjugate gradient method. The switching function was used to deal with long-range interactions. Moreover, the electrostatic and van der Waals interactions were considered based on a cutoff value 12.0 and a switchdist value of 8.0 Å throughout the simulation processes. To constrain all bonds between both hydrogen and heavy atoms, the SHAKE algorithm was applied^27^. Both the bonded and non-bonded forces were evaluated at every time step, and the electrostatic forces were evaluated by applying the particle mesh Ewald (PME) method^28^. In the next stage, a linear increase in the temperature of the system from 0 to 300 K within a time span of 600 ps. Furthermore, velocities were reassigned from a new Maxwell distribution, and temperature was increased by 0.001 K, at each integration step. During equilibration simulation of 1 ns, the system was allowed to relax in order to attain stable thermodynamic state. Langevin piston method was used for controlling the pressure^29,30^, and the temperature was controlled using Hoover thermostat^31^. In addition, the integration time-steps during heating and equilibration stages were set to 1 and 2 fs, respectively. During equilibration, a frame was recorded to trajectory file after every 1 ps of simulation. Final production run for each complex and the apo-form was carried out for 100 ns in NVE ensemble. MD trajectory obtained at this stage was used to sample the structural characteristics and dynamics of system.

#### Trajectory analysis

The collected trajectory was utilized to analyze several key properties of the complex. This analysis aided in identifying important motions and conformational changes that are relevant to the function and stability of the complex. Briefly, in order to understand the complex stability, the root mean-square deviation (RMSD) values for all protein atoms excluding hydrogens along with ligand atoms were calculated using VMD software. Furthermore, the root mean-square fluctuation (RMSF) was calculated to get insights into the conformational changes and local flexibility of the complexes. The obtained RMSD and RMSF values were plotted with respect to time in nanoseconds and chain-Cα residue numbers, respectively. Additionally, the radius of gyration (RoG) and solvent accessible surface area (SASA) values were calculated to assess the overall compactness of the complexes, along with the cluster analysis calculation to stand on the behavior of H-bonding throughout the simulation course.

The complete trajectory resulted from MD production was subjected to clustering technique of GROMACS using the g_cluster tool with a cutoff radius of 2 Å^32^. The resulted DCD trajectory files (obtained from NAMD) were converted to GROMACS readable XTC format using VEGAZZ software (http://www.vegazz.net/), taking into account, all frame numbers in the resulted XTC file format were converted to 0 by default during that conversion, so, to renumber the trajectory frames in the final XTC file, a trjconv command was used. The frames of the trajectory were clustered using g_cluster tool with an RMSD threshold of 0.11 nm, and the middle frame of the cluster that showed maximum number of frames was considered as the representative conformation. This representative structure was extracted, converted into pdb format using trjconv command and used as an input to the LigPlot^+^ software to again generate a 2D interaction plot between the ligand and the protein. The generated plot can be used to indicate the stability provided to the structure as a result of possible stable hydrogen bonds and hydrophobic contacts formed during MD simulation.

### In silico pharmacokinetics, toxicity predictions and drug-likeness properties

All the identified compounds (**41**) along with Spiramycin (**Spi**) were converted into their canonical simplified molecular-input line-entry system (SMILE), then they were submitted to the SwissADME^33^, PreADMET, ADMETlab 2.0 and Molsoft L.L.C. servers, in order to calculate their pharmacokinetic parameters including **A** (Absorption i.e., Human intestinal absorption (HIA), Caco-2, MDCK and Skin permeability), **D** (Distribution i.e., Plasma protein binding (PPB), P-glycoprotein substrate, inhibitor and C_Blood_-C_Brain_ barrier penetration (BBB)), **M** (Metabolism i.e., CYP family substrate/inhibitor), **E** (Excretion i.e., Elimination half-life (t_*1/2*_)) and **T** (Toxicity i.e., Ames mutagenesis and Carcinogenesis), along with the drug-likeness score^34^.

### Animals, parasites, materials and ethics

Our study is in accordance with ARRIVE guidelines (https://arriveguidelines.org). Briefly, this study was conducted at National Research Centre (NRC) and Al-Azhar Faculty of Medicine between June 2021 and July 2022. Swiss albino mice were obtained from Animal House—National Research Centre. Handling, anesthetic and sacrifice procedures^9^ followed ethical guidelines approved by the Ethical Committee of the Federal Legislation, the National Institutes of Health Guidelines in the United States and approved by the Research Ethics Committee of the Faculty of Medicine for Girls, Al Azhar University, Egypt, for the conduct of animal experiments (Ethics Approval No. 1574), a total of 70 laboratory-bred female Swiss Albino mice (20–25 g) were obtained from Laboratory Animals House, National Research Centre, Egypt, then housed in standard environmental conditions at temperature (24 °C) and relative humidity (50%) with a 12:12 light: Dark cycle, with free access to a standard commercial diet and water. Then were categorized into 7 groups (10 each), with the note that, for normal control **G-I**; it consisted of (5 non-infected and non-treated mice + 5 mice non-infected and treated only with a dose (0.72 mg/kg) for the detection of histopathological changes). The ME49 Avirulent strain of *T. gondii* was obtained from Zoonotic Diseases Department National Research Centre (NRC), Egypt. Mice were orally infected via esophageal tube starting from the first day of infection, and then were sacrificed at 6th day post infection. Spiramycin (**Spi**) tablets (1.5 mg) were purchased from Pharaonia Pharmaceuticals, Egypt. Spiramycin tablets were smashed into powder, dissolved in 15 mL water and given orally to mice using esophageal tube at a concentration (3MIU = 3000 mg).

## Experimental design and infection

### Experimental animals

Seventy Swiss Albino mice (20–25gm) were used to establish an animal model of acute *T. gondii* infection, and were randomly divided into 7 groups (each of 10 mice): **G-1**) ‘control’ non-infected–non-treated (– ve), **G-2**) infected untreated (+ ve), **G-3**) infected and treated with Spiramycin alone, **G-4**) infected and received *C. limon* MeOH extract, **G-5**) infected and received bergapten, **G-6**) infected and received isovitexin, and **G-7**) infected and received vitexin, after 60 days of infection, treatments were started daily for 21 days. The concentrations of MeOH ext., (100 mg/kg)^35^, vitexin, isovitexin^36^ and bergapten^37^ (1 mg/kg), were adjusted so that each 0.3 mL (2 mg/mice of MeOH ext., and 2 µg/mice of vitexin, isovitexin and bergapten) were administrated intra-esophageal per mice daily.

### Propagation of strain in vitro

Mice infection was performed at the Animal House of National Research Centre, by maintaining regular mice passage every 8 weeks for continuous collection of fresh cysts^38^, the obtained cysts from mice bran fluid were used for infection after dilution with 1mL saline and used for infection at dose of 25 cysts per mouse^39^.

### Euthanasia/sacrifice method

Swiss albino mice were euthanized via cervical dislocation (CD) method under anesthesia or tranquilization. Blood samples (2 ml/kg bw, once) were collected during scarification process. Animal death was confirmed via open chest inspection of the heart. All animals were euthanized after the protocol, using waste incinerator at National Research Centre.

### Endpoints

Starting treatment (50) days post-infection for 10 days. Mice will be sacrificed after the end of treatment (60 days post-infection).

### Parasite load (brain cyst burden)

After scarifications of the mice, the brain per mouse was homogenized with an equal volume of PBS at pH 7.4 and passed through 16-gauge needle ten times (by means of a syringe) to release tissue cysts^40,41^, followed by, 20 µL were placed on glass slides and left at room temperature for completely dryness, and then fixed using absolute methanol, after-that, it was stained using Gimsa stain for 1 min, and then washed with tap water, followed by cysts counting using bright-field microscope at (× 40). The average parasite load (APL/10 mg/tissue) was brain corresponding to chronic treatments, parasitic load was assessed by counting the total number of free bradyzoites as well as cysts/mg/brain in all groups^42^.

### Histopathological evaluation

The histological procedure used in this study adhered to methods outlined by Bancroft’s Theory and Practice of Histological Techniques^43^. Fresh tissue samples were carefully excised and immediately fixed in 10% neutral buffered formalin at room temperature for 24–48 h, ensuring optimal preservation of cellular and structural integrity. After fixation, tissues were dehydrated in a graded ethanol series (70%, 80%, 90%, and 100%), with each immersion lasting approximately one hour, adjusted based on tissue size and density. Following dehydration, tissues were cleared in xylene to remove ethanol and prepare for paraffin wax infiltration. The clearing was performed in two one-hour intervals to achieve tissue transparency, which allowed effective infiltration. Subsequently, tissues were infiltrated with molten paraffin wax at 60 °C for three to four hours, then embedded in paraffin blocks, which were allowed to cool and solidify to maintain tissue morphology. Embedded blocks were sectioned at 5 µm thickness using a microtome, and the sections were mounted on glass slides treated with adhesive to ensure adherence during staining.

Staining followed Bancroft’s protocol for hematoxylin and eosin (H&E): sections were first deparaffinized in xylene, then rehydrated through a descending ethanol series before hematoxylin staining for 3–5 min to highlight nuclei. After rinsing and differentiation, sections were counterstained with eosin for 1–2 min to enhance cytoplasmic details. Post-staining, sections were dehydrated, cleared in xylene, and mounted with synthetic resin. The slides were then examined under a light microscope, and images were captured for analysis. Rigorous quality control measures were implemented throughout all steps to ensure consistency, reproducibility, and accuracy in histological preparation and staining^43^.

### Statistical analysis

Data analysis was performed using SPSS software (vers-20) and were presented as mean ± SD. ANOVA (F) followed by post-hoc tests were performed to compare variables between the different groups. Significance level was set at *p* < 0.05, highly significant *p* < 0.001 and non-significant *p* > 0.05.

## Results and discussion

### Characterization of *C. Limon* metabolites

Our reported study characterized the detailed phytochemical investigation of the MeOH extract of *C. limon* leaves (Table 1), which produced the isolation of three known compounds and identified as bergapten (**17**)^44^, vitexin (**31**) and isovitexin (**32**)^45^, from fractions (**F**_**B**_, _**D & E**_) of the defatted MeOH ext., respectively, based on their NMR spectral data and comparison with literature (S_2–7). Moreover, one major fraction (**F**_**C**_) of the defatted MeOH ext., was scrutinized using LC-MS/MS (+ ve & – ve modes, S_8–12), and this led to the identification of forty compounds (**1**–**31**, **33**–**41**) in view of literature comparison of their mass spectral fragmentation patterns along with available standards (Table 1).

**Table 1:** LC-MS/MS of all identified compounds from *C. Limon* leaves MeOH Ext.

No.	MF	*R*_t_	Area %	[M - H, -2 H]^−^, CID (m/z)	[M + H, +2 H]^+^, CID (m/z)
1	C_7_H_12_O	0.79	1.23	**111**	
2	C_6_H_8_O_7_	7.21	0.52	**191**, *111*	
3	C_12_H_22_N_2_O_6_	9.38	0.65	**321**, *293*,* 275*,* 201*,* 157*,* 119*	
4	C_8_H_8_O_3_^*^	9.46	0.01	**151**, *136*,* 121*,* 108*	
5	C_7_H_6_O_5_^*^	3.89	0.002	**169**, *141*	
6	C_8_H_8_O_5_^*^	7.41	0.02	**183**, *182*,* 155*,* 154*,* 137*,* 124*,* 119*	
7	C_9_H_10_O_5_^*^	8.36	0.01	**196**, *181*	**199**, *183*,* 153*,* 126*
8	C_7_H_6_O_4_^*^	5.72	0.03	**153**, *135*,* 108*,* 107*	
9	C_9_H_8_O_4_^*^	8.03	10.45	**179**, *134*,* 108*	
10	C_10_H_10_O_4_^*^	10.19	0.13	**193**, *150*,* 133*	
11	C_9_H_8_O_3_^*^	5.01	19.35	**163**, *145*,* 135*,* 119*	**165**, *150*,* 121*
12	C_16_H_22_O_7_	14.06	0.98	**325**, *183*	**327**, *245*,* 172*,* 118*
13	C_16_H_22_O_7_	7.02	9.05	**341**, *341*,* 295*,* 209*,* 179*,* 161*,* 113*	
14	C_18_H_18_O_2_	14.11	1.51	**265**, *265*,* 179*	
15	C_12_H_16_O_2_	6.82	2.40	**191**, *176*,* 148*,* 104*	
16	C_9_H_6_O_4_	8.08	11.02	**177**, *162*,* 133*,* 121*,* 117*	
17^a^	C_12_H_8_O_4_				**217**, *202*,* 174*
18	C_20_H_22_O_4_	14.00	0.50	**325**, *279*,* 183*	**327**, *325*,* 307*,* 183*
19	C_19_H_20_O_4_	11.28	1.31	**311**, *293*,* 185*,* 171*	
20	C_15_H_20_O_2_	7.64	1.90	**231**	**233**, *232*,* 217*,* 189*,* 172*,* 161*,* 133*
21	C_36_H_38_O_11_	9.75	0.61		
22	C_30_H_20_O_8_	6.54	0.58	**507**	**509**, *346*,* 331*
23	C_20_H_18_O_9_	8.87	2.35	**401**, *401*,* 355*,* 193*,* 175*	
24	C_20_H_20_O_7_	7.73	0.91	**371**	**373**, *341*,* 281*,* 229*,* 215*,* 203*
25	C_20_H_20_O_7_	7.56	1.91	**371**, *371*,* 317*,* 249*,* 121*	**373**, *229*,* 215*,* 121*
26	C_21_H_22_O_9_	6.69	0.49	**417**, *371*,* 295*,* 149*,* 151*,* 121*	**419**, *387*,* 401*,* 310*,* 278*
27	C_15_H_10_O_6_^*^	13.46	0.003	**285**, *229*,* 185*	**286**, *201*,* 187*
28	C_19_H_18_O_6_	7.30	3.35	**341**, *282*,* 267*,* 134*	
29	C_19_H_18_O_6_	7.40	3.17	**341**, *311*,* 265*,* 251*,* 171*	
30	C_18_H_16_O_6_	7.96	3.43	**326**	**328**, *178*,* 149*,* 121*
31^b^	C_21_H_20_O_10_	5.97	16.55	**431**, *385*,* 341*,* 325*,* 311*,* 223*,* 209*	
32^c^	C_21_H_20_O_10_				
33	C_22_H_22_O_11_	6.26	2.76	**461**, *460*,* 446*,* 415*,* 299*,* 282*	
34	C_27_H_30_O_14_	6.47	0.54	**577**, *432*,* 270*	**579**, *433*,* 271*
35	C_15_H_10_O_7_^*^	13.53	0.01	**301**, *283*,* 255*,*212*,* 113*	
36	C_27_H_30_O_16_	9.72	0.94	**609**, *299*	**612**, *611*,* 402*,* 356*,* 355*,* 354*,* 220*
37	C_16_H_14_O_6_^*^	15.51	0.007	**301**, *179*,* 151*,* 121*,* 107*	
38	C_15_H_12_O_5_	14.89	0.003	**271**, *225*,* 223*	
39	C_27_H_30_O_14_	9.10	2.37	**577**	**579**, *578*,* 433*,* 271*
40	C_22_H_22_O_12_	6.61	1.60	**477**, *476*,* 462*,* 315*,* 314*,* 300*,* 292*	
41	C_28_H_32_O_16_	6.42	0.42	**623**, *622*,* 337*,* 323*,* 315*	

^*^: Standard, ^a^: Isolated from **F**_**B**_ (C1, 0.4 g), ^b^: Isolated from **F**_**D&E**_ (C3, 0.6 g), ^c^: Isolated from **F**_**D&E**_ (C3, 0.5 g).

The identified metabolites (Fig. 1) were grouped into eleven classes including; an unsaturated fatty acid identified as sorbic acid (**1**)^46^, two tricarboxylic acids identified as *iso*-citric acid (**2**) and avenic acid A (**3**)^47,48^. Moreover, a phenolic aldehyde characterized as vanillin (**4**), and seven phenolic acids identified as gallic acid (**5**), methyl gallate (**6**), syringic acid (**7**), protocatechuic acid (**8**), caffeic acid (**9**), ferulic acid (**10**) and *ρ*-coumaric acid (**11**), based on comparison with standards. Furthermore, two phenolic glycosides termed as citrusin-C (**12**)^14^ and citrusin-D (**13**)^49^, and one lignan named magnolol (**14**)^50^. In addition, five coumarins described as scopoletin (**15**)^51^, esculetin (**16**)^52^, bergapten (**17**) isolated from **F**_**B**_^44^, dentatin (**18**)^53^, and nordentatin (**19**)^54^. Besides, a sesquiterpene lactone of the eudesmanolide group known as *iso*-alantolactone (**20**)^55^, a limonoid identified as voamatin-D (**21**)^56^ and a steroid characterized as contignasterol (**22**)^57^.

Additionally, eighteen flavonoids distinguished into; nobeltin (**23**) and *iso*-sinensetin (**24**)^58^, sinensetin (**25**)^59^, 5-hydroxy-3,6,7,8,3′,4′-hexamethoxyflavone (**26**)^60^, luteolin (**27**) (standard), luteolin tetramethyl ether (**28**)^61^, scutellarein tetramethyl ether (**29**)^62^, *iso*-scutellarein 7,8,4′-trimethyl ether (**30**)^63^, vitexin (**31**) and isovitexin (**32**)^45^, chrysoeriol 7-glucoside (**33**)^47^, apigenin 7-rutinoside (**34**)^64^, quercetin, rutin, hesperetin, naringenin (**35**–**38**) (standards), naringin (**39**)^65^, isorhamnetin 3-glucoside (**40**)^66^ and isorhamnetin 3-neohesperidoside (**41**)^45^.

As clearly appeared in Table 1, all the identified compounds recorded the characteristic MS^2^ fragment ions attributed for their different classes; including decarboxylation [M-CO_2_]^−^ and dehydration [M-H_2_O]^−^ for phenolic acids, in addition to, the loss of the hexose moiety [M-162]^−^ as in phenolic glycosides^67,68^. Furthermore, flavonoid glycosides recorded the loss of their sugar moieties to give the corresponding aglycone, followed by removal of H, OH, CO and/or RDA cleavage^69^. Besides that, a noteworthy loss of CO or CO_2_ which is a characteristic for coumarins^70,71^.

## Parasitological results

### Inhibition rate of tachyzoites in vivo

The inhibition rate experiment was carried out in order to stand on the degree of *T. gondii* tachyzoites inhibition, as clearly summarized in (Table 2), the number of tachyzoites invading host cells was highly recorded upon infection (**G-2**), whereas, it was reduced significantly upon the administrated of *C. limon* MeOH ext., alongside bergapten, vitexin and isovitexin, intra-esophageal (0.3 mL/mice) daily, with 32.45% recorded for bergapten compared with 39.62% listed for **Spi**, followed by *C. limon* MeOH extract, vitexin and isovitexin, with 45.28, 48.30 and 71.32% respectively.

**Table 2:** The mean of tachyzoite reduction rate after different treatment regimens.

Group	No.	No. of tachyzoites		Red. rate (%)	Pairwise group significance					
					*P* against other groups in order					
		Mean	SD		G-2	G-3	G-4	G-5	G-6	G-7
G-2	10	2650.0	168.40	**–**	**–**	< 0.001	< 0.001	< 0.001	< 0.001	< 0.001
G-3	10	1100.0	134.40	39.62%	< 0.001	**–**	0.007	< 0.001	< 0.001	< 0.001
G-4	10	1200.0	148.21	45.28%	< 0.001	0.007	**–**	0.054	0.519	< 0.001
G-5	10	860.0	138.22	32.45%	< 0.001	< 0.001	0.054	**–**	0.184	0.002
G-6	10	1890.0	145.97	71.32%	< 0.001	< 0.001	0.519	0.184	**–**	< 0.001
G-7	10	1280.0	101.87	48.30%	< 0.001	< 0.001	< 0.001	0.002	< 0.001	–

p **<** 0.05: Significant., p **<** 0.001: Highly Significant., *p* < 0.05: Non-Significant.

### Histopathological results

**Fig. 2:**
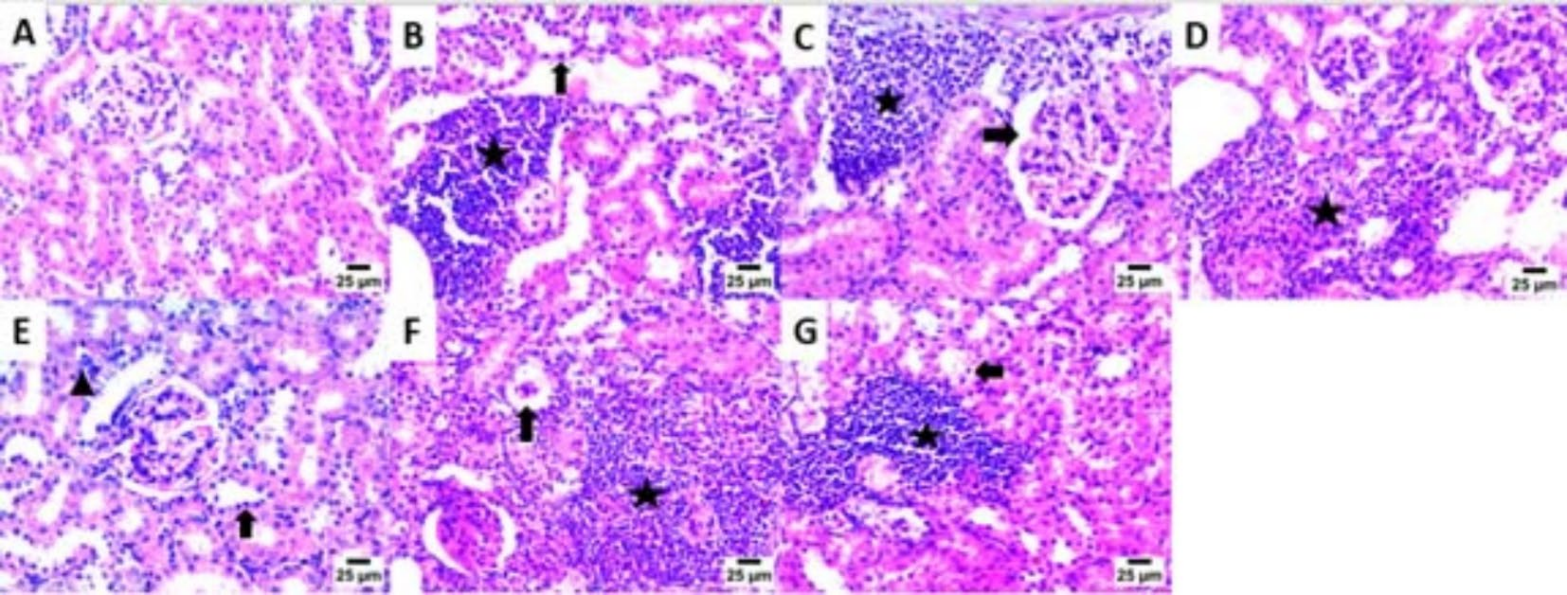
Photomicrograph of the Kidney showing: (**A**) Normal histological structure of glomeruli and renal tubules [**G-1**]. (**B**) High number of mononuclear inflammatory cells between renal tubules (star) and presence of nuclear pyknosis in renal tubular epithelium (arrow) [**G-2**]. (**C**) High number of mononuclear inflammatory cells between renal tubules (star) and increase of urinary space (arrow) [**G-3**]. (**D**) High number of mononuclear inflammatory cells between renal tubules (star) [**G-4**]. **E**) Low number of mononuclear inflammatory cells between renal tubules (arrowhead) and presence of nuclear pyknosis in renal tubular epithelium (arrow) [**G-5**]. (**F**) High number of mononuclear inflammatory cells between renal tubules (star) and presence of collapsed glomeruli (arrow) [**G-6**]. (**G**) High number of mononuclear inflammatory cells between renal tubules (star) and presence of necrobiotic changes including pyknotic nuclei and vacuolar degeneration in renal tubular epithelium (arrow) [**G-7**], (200×).

The main part of histopathology is the liver, which plays a vital role in harmonizing adaptation of innate immune responses, whereas, the kidney as a vital organ with high energy demand plays a key role in regulating the disease related metabolic process^72^. Consequently, kidney and liver indices are used to assess the protective effect of drugs on viscera. As clearly appeared in (Fig. 2A–G). Kidney sections of the infected mice with *T. gondii*, revealed high mononuclear inflammatory cells with the mean (SD) number 791.10 (32.07), which unfolded between renal tubules together with the presence of nuclear pyknosis in renal tubular epithelium, these inflammatory cells were significantly decreased upon treatment with the tested drugs; bergapten, **Spi**, *C. limon* MeOH ext., vitexin and isovitexin, with the mean (SD) numbers; 56.80 (14.59), 291.60 (30.87), 401.90 (15.45), 474.80 (20.39) and 617.50 (28.15) respectively, as documented in (Fig. 4).

**Fig. 3:**
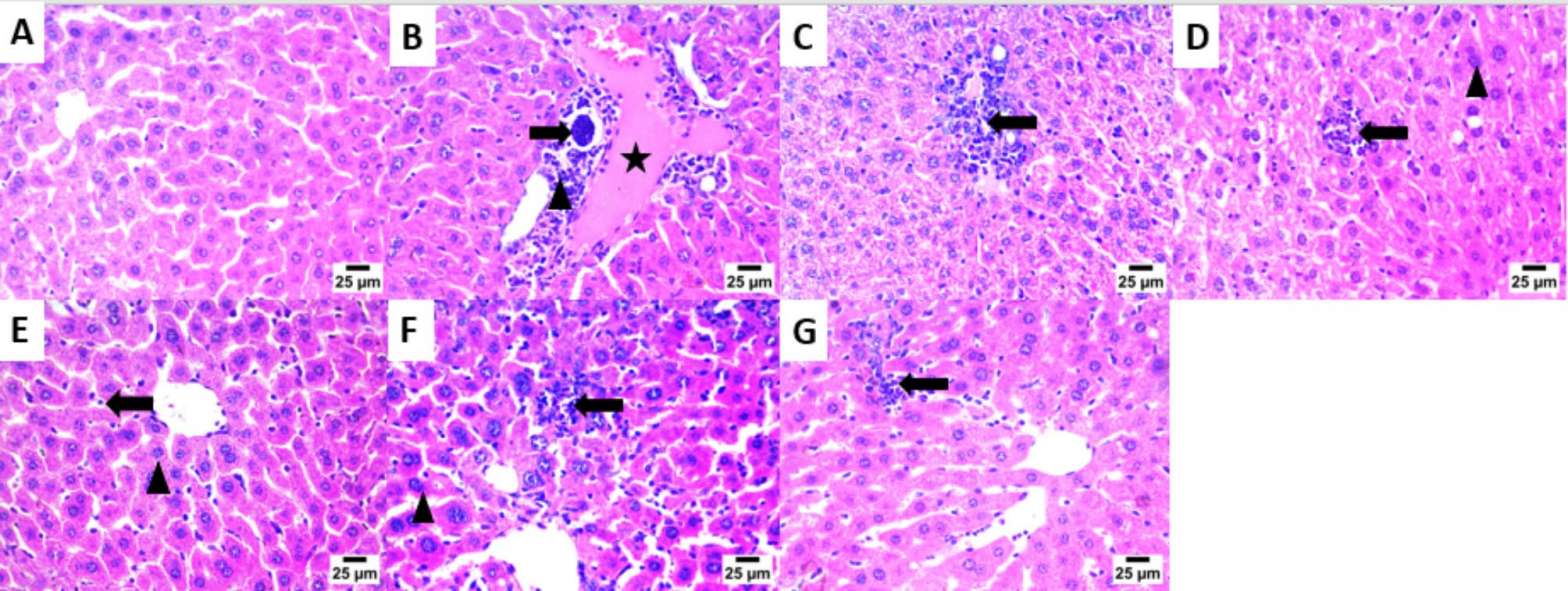
Photomicrograph of the Liver showing: (**A**) Normal histological structure of hepatocytes and hepatic sinusoids [**G-1**]. (**B**) Bradyzoites cyst in portal area (arrow) with infiltration by mononuclear inflammatory cells (arrowhead) and presence of congestion in portal blood vessels (star) [**G-2**]. (**C**) Infiltration of portal area by mononuclear inflammatory cells (arrow) [**G-3**]. (**D**) Aggregates of mononuclear inflammatory cells between hepatocytes in hepatic sinusoids (arrow) and presence of presence of nuclear pyknosis in some hepatocytes (arrowhead) [**G-4**]. (**E**) Low number of mononuclear inflammatory cells in hepatic sinusoids (arrow) and presence of nuclear pyknosis in some hepatocytes (arrowhead) [**G-5**]. (**F**) High number of mononuclear inflammatory cells between hepatocytes in hepatic sinusoids (arrow) and presence of nuclear pyknosis in some hepatocytes (arrowhead) [**G-6**]. (**G**) Presence of moderate number of mononuclear inflammatory cells in hepatic sinusoids (arrow) [**G-7**], (200×).

**Fig. 4:**
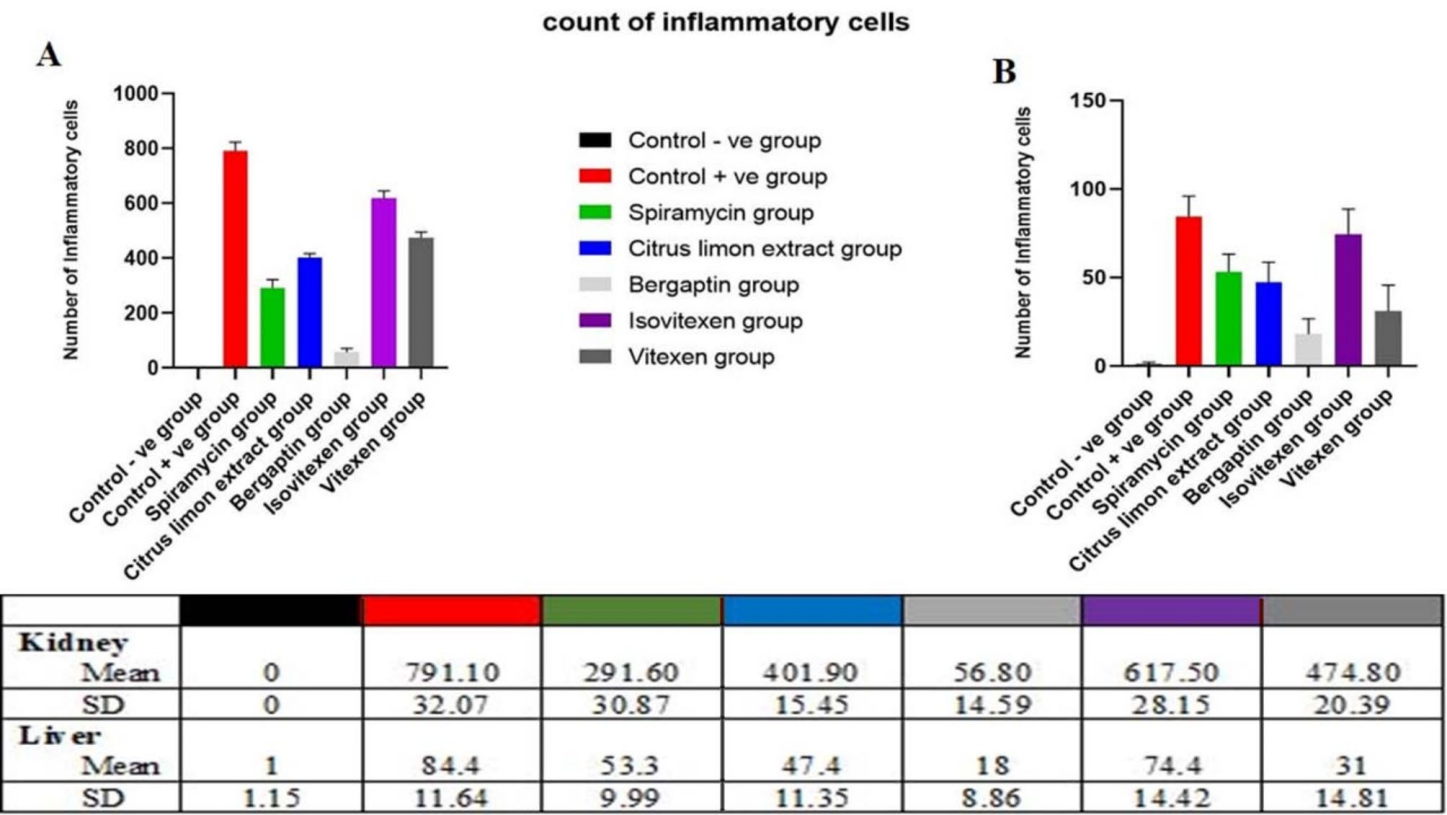
Inflammatory cells count and data represented as a mean ± SD (*n* = 10), values indicated that, bergapten group was significantly variable than other group (*P* ≤ 0.0001) according to one-way ANOVA and Tukey tests.

Moreover, sections in the liver (Fig. 3A–G) of *T. gondii* infected non-treated mice (**G-2**) showed bradyzoites cyst in portal area with infiltration by mononuclear inflammatory cells and the presence of congestion in portal blood vessels, compared to normal histological structure of hepatocytes and hepatic sinusoids (**G-1**), with mononuclear inflammatory cell count appeared in (Fig. 4).

Interestingly, bergapten treated group (**G-5**) displayed mild mononuclear inflammatory cells aggregate between hepatocytes in hepatic sinusoids (Fig. 4E) and the presence of nuclear pyknosis in some hepatocytes. Additionally, aggregation of moderate numbers of mononuclear inflammatory cells in hepatic sinusoids were appeared in **Spi**, *C. limon* MeOH ext., and vitexin treated groups **G-3**, **-4** and **-7**, respectively.

It is well known that immune-competent individuals infected with *T. gondii* often acquired latent chronic infection, with parasite cyst formation in brain tissue, and as a result of wanes in infected individuals’ immunity, acute infection and toxoplasmosis manifestation takes place^73^.

**Fig. 5:**
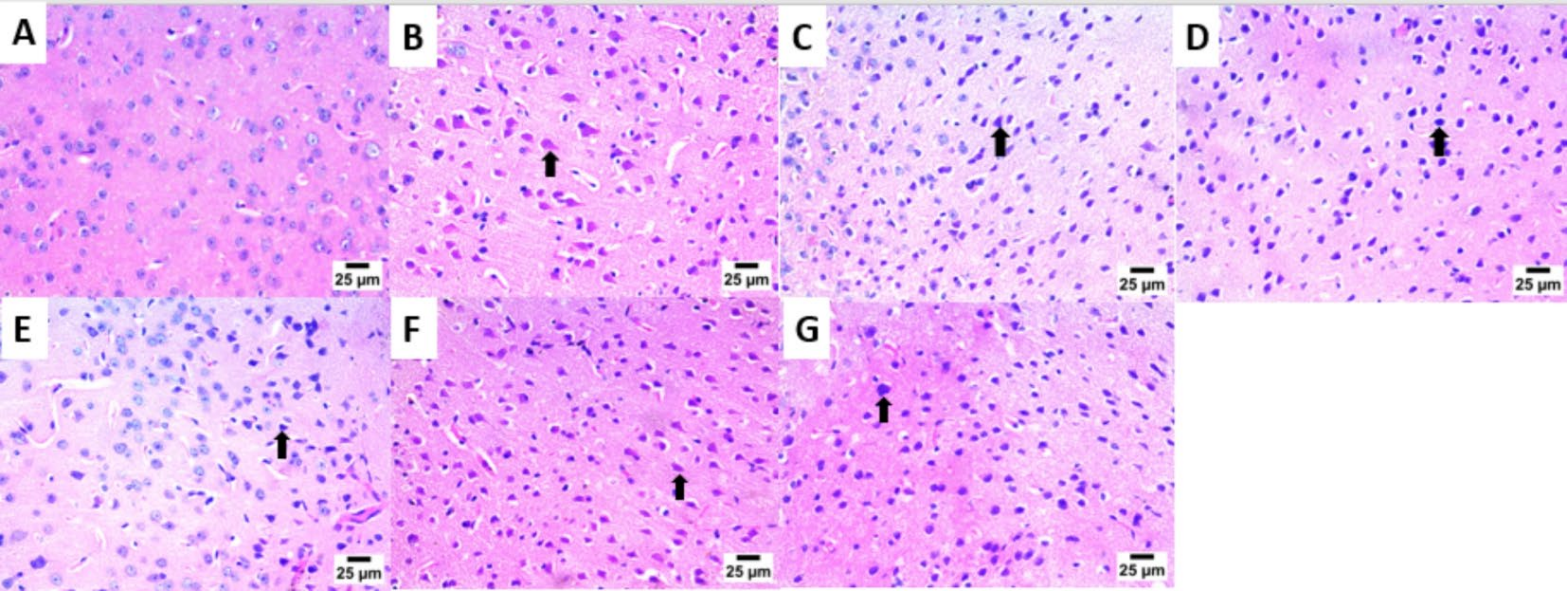
Photomicrograph of the Brain showing: (**A**) Normal histological structure of striatum [**G-1**]. (**B**) High number of necrotic neurons with acidophilic cytoplasm and nuclei (arrow) [**G-2**]. (**C**) Moderate number of shrunken and degenerated neurons with pyknotic nuclei (arrow) [**G-3**]. (**D**) High number of shrunken and degenerated neurons with pyknotic nuclei (arrow) [**G-4**]. (**E**) Low number of shrunken and degenerated neurons with pyknotic nuclei (arrow) [**G-5**]. (**F**) Moderate number of necrotic neurons with acidophilic cytoplasm and nuclei (arrow) [**G-6**]. (**G**) High number of shrunken and degenerated neurons with pyknotic nuclei (arrow) [**G-7**], (200×).

The brain tissues (Fig. 5A–G) of *T. gondii* infected non-treated mice showed high number of necrotic neurons with acidophilic cytoplasm and nuclei (**G-2**). Furthermore, high number of shrunken and degenerative neurons with pyknotic nuclei was observed in *C. limon* MeOH ext. and vitexin treated groups (**G-4** and **G-7**, respectively). However, the number of shrunken and degenerative neurons with pyknotic nuclei was moderate in **Spi** and isovitexin treated groups (**G-3** & **G-6**, respectively). Interestingly, the number of shrunken and degenerated neurons with pyknotic nuclei was lowered significantly in case of bergapten treated group (**G-5**). The cellular immune response affected highly the rate of ME 49 parasite strain, which explains the observed reduction in the number of liver cysts upon infection^74^.

Histopathological outcomes reflected the significant effect of the tested drugs on the kidney, liver and brain in *T. gondii* infected non-treated mice, with which we can infer that, a sever tissue damage presented in these tissues, with extensive vacuolar degeneration, necrosis of hepatocytes, isolated regions of inflammatory cell infiltration and congestion of hepatic blood vessel. The free tachyzoites attracted inflammatory cells, which is then sparked an inflammatory response and led to cell lysis within tissue sections compared to the healthy control and treated mice, these results are consistent with other reports from earlier investigations^75,76^.

Histopathological investigations promoted notable improvements including mild focal aggregation, decrease in the inflammatory cell infiltration, vacuolar degeneration of hepatic cells, as well as shrunken and degenerated neurons with pyknotic nuclei, in the tissues of kidney, liver and brain, respectively, with the tested drugs in the order, bergaptin, **Spi**, *C. limon* MeOH ext., isovitexin and vitexin.

The noticed inhibition could be attributed to the phenolic nature of bergapten, isovitexin and vitexin, which are well known antioxidants as reported throughout the literature, besides that, *C. limon* MeOH ext. is a rich source with different active metabolites, which all work synergistically to inhibit the parasite antioxidant metalloenzymes (i.e., superoxide dismutase and catalase), which are important for the antioxidant protection of the parasite by generating oxidative stress, and hence, alter parasite morphology and disrupt cell cycle^77^.

Briefly, Olanrewaju and co-workers^78^ reported that plant siderophores i.e., avenic acid (a linear hydroxy-imino-carboxylic acid) bind with iron more efficiently than bacteria siderophores. Moreover, Piao and colleagues^79^ mentioned that, phenolic acids e.g., gallic acid, ethyl gallate and protocatechuic acid effectively prolong the survival time of mice acutely infected with *T. gondii*, due to their ability to exert antioxidant activity based on the presence of phenolic hydroxy i.e., vitexin and isovitexin.

Furthermore, Abugri and others^80^ proved that quercetin, a well-known antioxidant, is able to dysfunction the mitochondria membrane of both intracellular and extracellular *T. gondii* tachyzoites. Besides that, Jiang and associates^81^ stated that, plant natural phenolics are able to reduce the number of extracellular grown tachyzoites by disrupting the redox homeostasis of the parasite, and hence, promote apoptosis in *T. gondii*. In addition, Elazab and co-workers^82^ proved the anti-toxoplasmosis of citrusin-C and -D identified in some Egyptian plants based on their ability for iron chelation. We can conclude that, flavonoids and phenolic compounds work against many extracellular and intracellular protozoan parasites^83^, they displayed their mechanisms via blocking the cytoplasmic membrane function, preventing bacterial virulence factors, the suppression of energy metabolism and nucleic acid synthesis, and work synergistically with current synthetic drugs, etc^84,85^. Besides that, they can brace the immune system, especially the cellular one through stimulating natural killer cells and macrophages, phagocytosis, regulation of cytokine excretion and immunoglobulins production^86,87^. Hence, it can be acclaimed that *C. limon* MeOH ext. displayed prophylactic effects against toxoplasmosis induced by *T. gondii*, through the direct effect on parasites and also indirect mechanisms, especially ones that reinforce the immune system, mainly the cellular immune system.

### Molecular docking study

In order to demonstrate and benchmark our obtained fundamental biochemical results, we had to define the action of the small molecules (**1**–**41**) at the binding site of the target protein receptors, which had been achieved via molecular docking approach, that models the binding interactions between a small molecule and a target receptor at the atomic level. A detailed in silico study was carried out to examine all identified compounds (**1**–**41**) against selected protein targets as reported in (**S_13**).

In brief, rhoptry proteins i.e.,* Tg*ROP5 and *Tg*ROP18 are unique kinases from the rhoptry organelles of the apicomplexan phylum. They are involved in the invasion and egress of the parasite, as they are essential for their internalization and manipulation of the host immune response. Reese and Boothroyd^88^, and Reese and co-workers^89^, reported that, ROP5 plays a key role in *T. gondii* pathogenesis, as it affects critically the subcellular localization of specific receptors, recognition of its partner ligands and regulates signaling networks, moreover, Behnke and colleagues^90^, demonstrated that, *Tg*ROP5 controls the virulence of *T. gondii* by regulating the active kinase ROP18. In addition, Lim and others^91^ stated that, ROP18 is a key factor for virulence modulation of *T. gondii*, as it controls the intercellular proliferation of the parasite, and manipulates the host’s immunity and cell apoptosis^92,93^.

As a consequence, the above mentioned roles make *Tg*ROP5 and *Tg*ROP18 perfect targets for *T. gondii* inhibition, through a mechanism of binding and disrupting its pathogenesis process, as clearly appeared in (**S_13**) all the docked ligands (**1**–**41**) anchored well in the binding site of *Tg*ROP5 and *Tg*ROP18, and bonded to the key amino acids of the active pocket with binding affinities of (− 9.47 to − 4.38) and (− 8.58 to − 4.22) kcal/mol respectively, with RMSD < 2 Å which is closest to and even consider better than **Spi** of binding energy (− 9.67 and − 10.18) kcal/mol respectively, and RMSD > 2 Å at the active pocket of most tested targets.

**Fig. 6:**
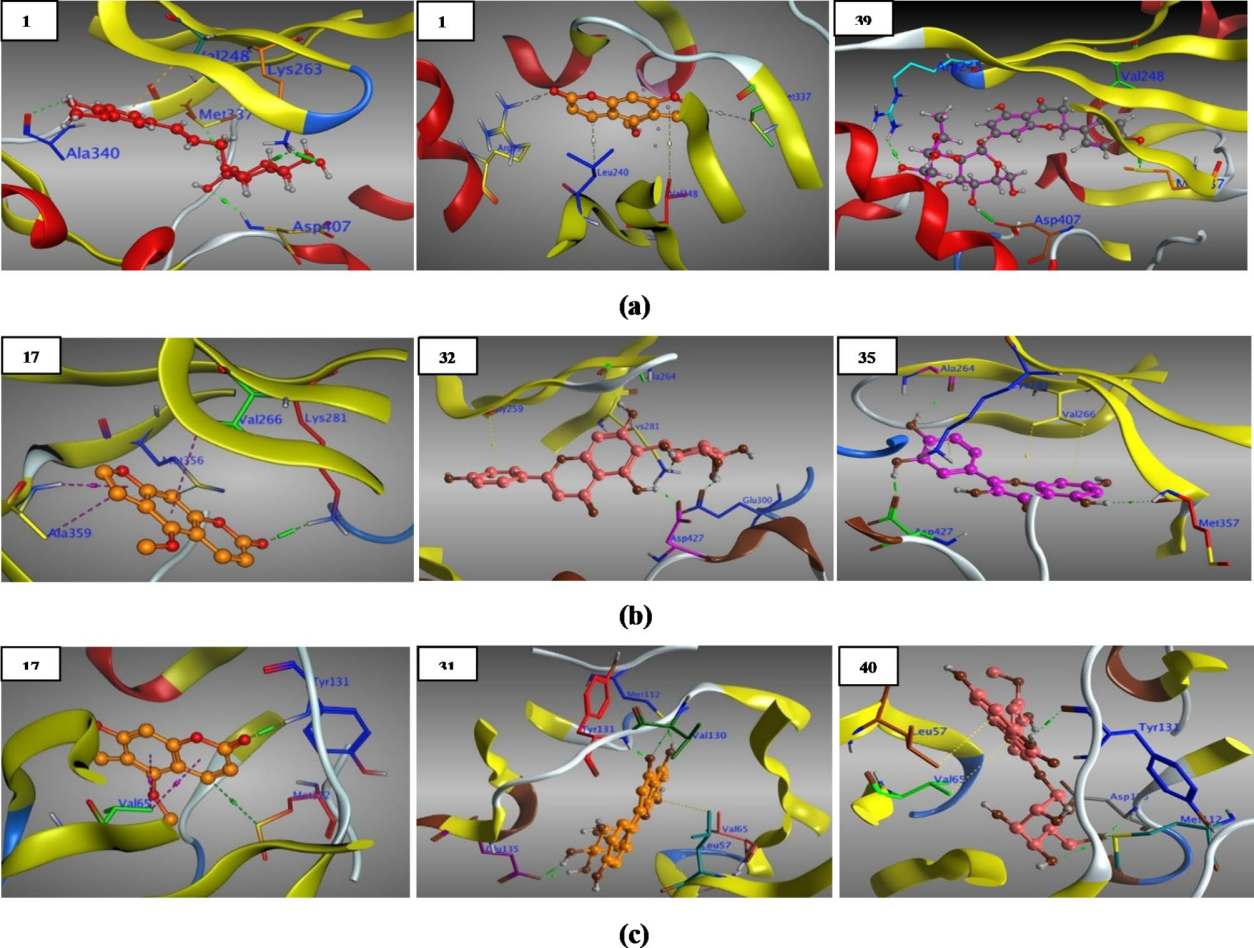
(**a**) 3D Structure of the docked poses of citrusin-D (**13**), bergapten & naringin (**39**) with 3Q5Z. (**b**) 3D Structure of the docked poses of bergapten (**17**), isovitexin (**32**) & quercetin (**35**) with 4JRN. (**c**) 3D Structure of the docked poses of bergapten (**17**), vitexin (**31**) & isorhamnetin 3-glucoside (**40**) with 4M84.

Briefly, for *Tg*ROP5 (3Q5Z), the active pocket’s key amino acids were (**Arg241**,** Arg245**,** Val248**,** Lys263**,** Phe265**,** Glu275**,** Leu279** and **Met337**) as reported^90^, out of the frontrunner compounds (**1**–**41**), only two compounds **13** and **39** (Fig. 6a) created strong and stable interactions with the same 3 key amino acids along with others, while bergapten (**17**), vitexin (**31**) and other ligands (**12**, **18**, **19**, **22**–**24**, **26**–**28**, **33**, **34**, **36**, **40**, **41**) displayed interactions with 2 key amino acids, in addition, isovitexin (**32**) along with ligands (**3**, **5**–**8**, **10**, **14**–**16**, **20**, **21**, **25**, **29**, **30**, **35**, **37**, **38**) revealed interactions with only one key amino acid, moreover, compounds (**1**, **2**, **4**, **9**, **11**) interacted with other amino acids in the active pocket.

In addition, *Tg*ROP18 key amino acids (**S_13**) were reported by Molina and co-workers^94^, based on their important roles; as catalytic residues (i.e., **Lys281**, ** Ala359**, **Asp409**, **Lys411**, **Asn414**, **Asp427**), to hold the substrate in the active site (i.e., **Gly259**, **Gly261**, **Gly262**, **Phe263**, **Val266**), as conserved and regulatory residues (i.e., **Phe428**, **Gly429**), as a key residue for salt-bridge formation (i.e., **Lys281**, **Glu300**) and finally as a gatekeeper cover the hydrophobic pocket (**Met356**).

In brief, as clarified in (**S_13**) from the total list, three compounds, bergapten (**17**), isovitexin (**32**) and quercetin (**35**) (Fig. 6b) interacted strongly with 5 key amino acids, moreover, compounds **3**, **16**, **27** and **33** bonded to 4 key amino acids and others, furthermore, compounds (**4**, **9**–**11**, **13**, **15**, **19**, **34**, **36**, **40**, **41**) formed interactions with 3 key amino acids and with others, additionally, vitexin (**31**) and compounds (**2**, **5**–**8**, **12**, **14**, **18**, **22**, **25**, **26**, **37**–**39**) revealed 2 key amino acids together with other interactions, besides, one key amino acid interaction and others were reported for compounds (**1**, **20**, **21**, **23**, **24**, **28**, **29**, **30**).

Based on a report by Zhang and co-workers^95^, *Tg*CDPK1 (4M84) was confirmed as a life cycle fundamental for the parasite mobility including invasion and egress, as it have been implicated in specific functions and in the development of distinct stages of its complex life cycle, once again, back to (**S_13**) putting in our mind the key amino acids (**Leu57**,** Val65**,** Met112**,** Glu129**,** Tyr131**,** Glu135**,** Asp195**) of the active pocket of *Tg*CDPK1, we can concluded that, all the docked compounds fit in the active cavity and recorded binding affinities of (– 8.75 to – 4.55) kcal/mol, out of the frontrunner list, two compounds, vitexin (**31**) and isorhamnetin 3-glucoside (**40**) (Fig. 6c) revealed 5 key amino acids interactions, in addition, bergapten (**17**) (Fig. 6c) and another five compounds (**6**, **24**, **28**, **35**, **36**) interacted with 4 key amino acids and others, moreover, five compounds (**4**, **5**, **9**, **16**, **39**) showed 3 key amino acids interactions, compounds (**1**, **10**, **12**, **14**, **18**, **25**, **26**, **29**, **34**, **37**, **38**) displayed one key amino acid together with other amino acids interaction, while isovitexin (**32**) and compounds (**2**, **3**, **7**, **8**, **11**, **13**, **15**, **19**, **23**, **27**, **30**, **41**) showed two key amino acids along with other interactions, finally, compounds (**20**–**22**, **33)** interacted with amino acids, other than the key, at the binding pocket of the active site.

Consequently, we set a directed docking approach targeting parasite’s DNA and RNA, as rational lethal targets for the design of specific anti-toxoplasma subversive substrates^96^, and hence could affect *T. gondii*’s DNA replication, transcriptional and translational activities through the following; uracil phosphoribosyltransferase ‘UPRTase’ (1UPF) for pyrimidine synthesis^97^, adenosine kinase ‘AK’ (3MB8 & 1LIJ) for purine synthesis and recovery, respectively^98,99^, thymidyl synthase ‘Ts’ (4KY4) for nucleotide synthesis^100,101^, enoyl acyl reductase carrier protein ‘ENR’ (2O2S) required for parasite’s fatty acid chains synthesis^102^, prolyl tRNA synthetase (6A88) for parasite’s protein translational^103^, and finally a good target that could be interfered with and deregulated the parasite invasion, it is macrophage migration inhibitory factor ‘MMIF’ (4DH4), which plays an immunomodulatory role^104^.

All the docked ligands (**1**–**41**) embedded well in the active pocket of the selected proteins (**S_13**) with relatively high free binding energies compared to **Spi**, and even with better RMSD < 2 Å, most of the docked ligands interacted with the same key and/or others amino acids within the active pocket and hence can combat against toxoplasmosis.

**Fig. 7:**
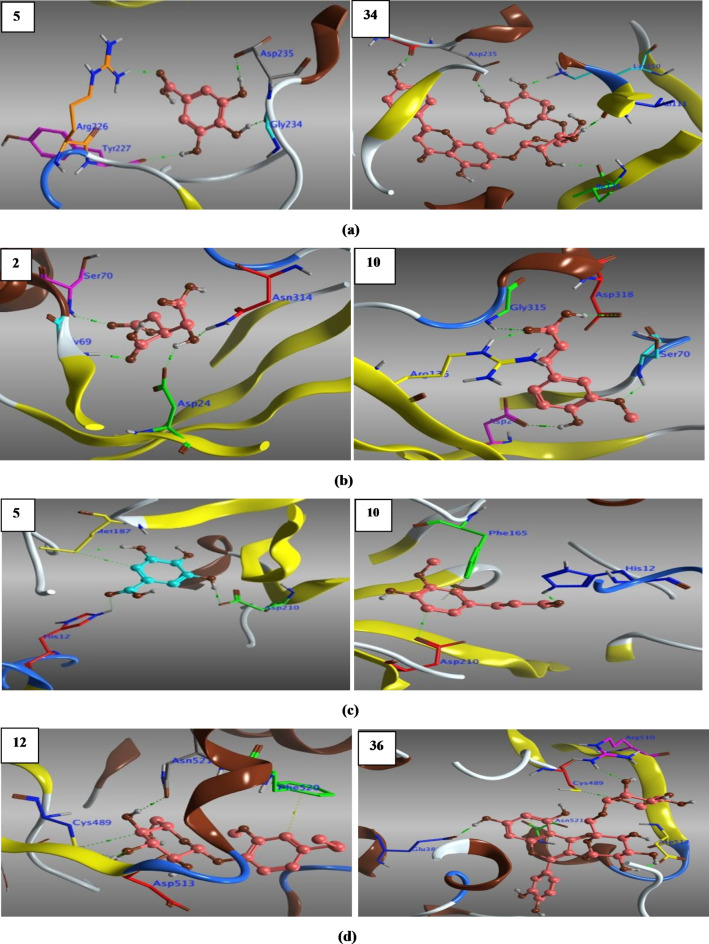

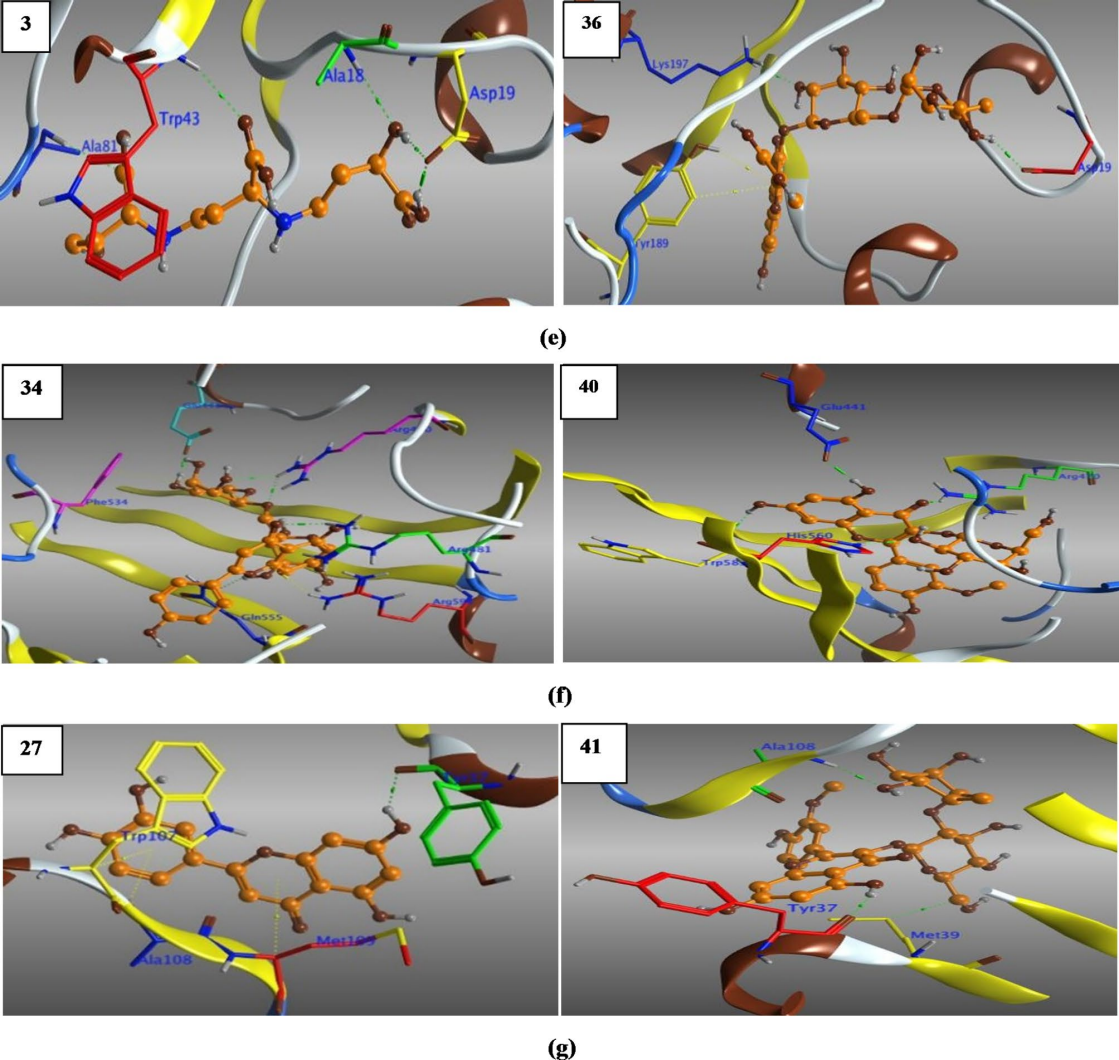
**(a)** 3D Structure of the docked poses of gallic acid (**5**) & apigenin 7-rutinoside (**34**) with 1UPF. (**b)** 3D Structure of the docked poses of *iso*-citric acid (**2**) & ferulic acid (**10**) with 1LIJ. (**c)** 3D Structure of the docked poses of gallic acid (**5**) & ferulic acid (**10**) with 3MB8. (**d)** 3D Structure of the docked poses of citrusin-C (**12**) & rutin (**36**) with 4KY4. (**e)** 3D Structure of the docked poses of avenic acid A (**3**) & rutin (**36**) with 2O2S. (**f)** 3D Structure of the docked poses of apigenin 7-rutinoside (**34**) & Isorhamnetin 3-glucoside (**40**) with 6A88. (**g)** 3D Structure of the docked poses of luteolin (**27**) & isorhamnetin 3-neohesperidoside (**41**) with 4DH4.

Concisely, as shown in (**S_13**) out of the frontrunner list, compounds **5** and **34** (Fig. 7a) fit well in the active pocket of 1UPF and hence struck pyrimidine synthesis of *T. gondii* via HB-interactions with the same key amino acids (**Gly234**,** Asp235**) along with others. Moreover, compounds **2** and **10** (Fig. 7b) influenced parasite purine synthesis pathway through hydrogen bonding with the key amino acids of the active site of 1LIJ (**Asp24**,** Gly69**,** Ser70**,** Asn314**) and (**Asp24**,** Gly69**,** Ser70**,** Arg136**,** Gly315**,** Asp318**) respectively. Furthermore, compounds **5** and **10** (Fig. 7c) affected purine recovery of *T. gondii* by interaction with key amino acids (**His12**,** Met187**,** Asp210**) of 3MB8 active site. In addition, compounds **12** and **36** (Fig. 7d) combated *T. gondii* growth by suppressing its nucleotide synthesis via HB interactions with key amino acids (**Cys489**,** Asp513**,** Asn521**) of 4KY4 energetic pocket. Besides that, compounds **3** and **36** (Fig. 7e) suppressed best the synthesis of *T. gondii* fatty acids chains by interaction with (**Trp43**,** Ala81**) and (**Tyr189**,** Lys197**) of 2O2S active pocket, respectively. Additionally, compounds **34** and **40** (Fig. 7f) bonded to the key amino acids (**Glu441**,** Arg470**,** Arg481**,** Gln555**) and (**Glu441**,** Arg470**,** His560**) respectively, and subverted *T. gondii* protein (6A88) translation. Finally, compounds **27** and **41** (Fig. 7g) were recorded good interactions with the key amino acid **Tyr37** and others of the robust pocket of 4DH4, and hence could be interfered with and deregulated the parasite invasion.

Assessment of our molecular docking outcomes was presupposed on receptor–ligand complementarity considering electrostatic and steric characteristics together with the calculated potential interaction energy in the complex and ligand intramolecular interactions i.e., (HB-donor and/or -acceptor, H-π, π-π, π-cation), which play a supreme role in energetically stabilizing ligands at the binding site of protein receptors. In our study, H-bond interaction of protein/ligand was the predominated as it modulates their binding affinity as a result of setting up of like-for-like synergistic interactions, besides other hydrophobic molecular interactions^105^.

**Fig. 8:**
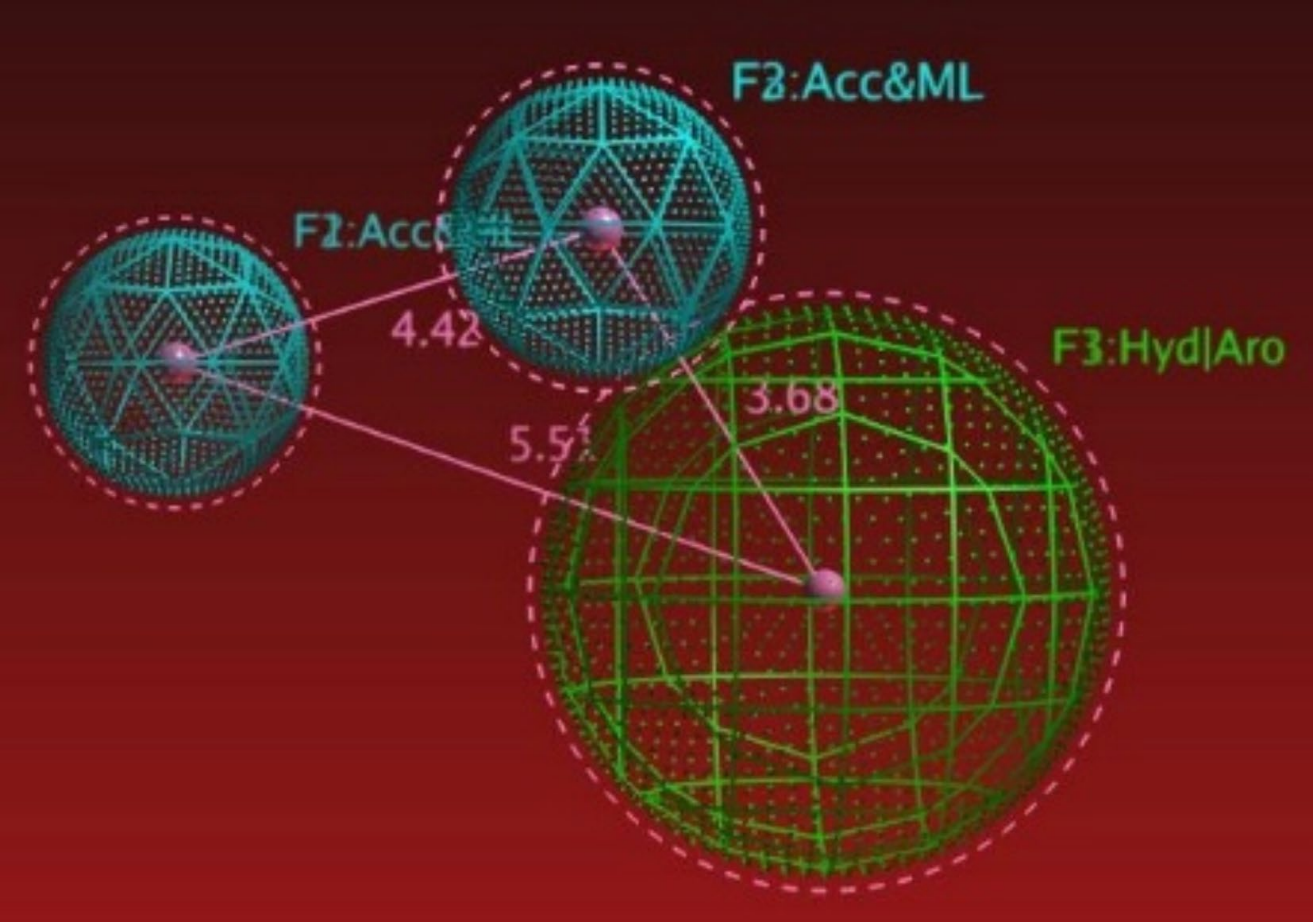
3D pharmacophoric features.

Finally, in order to emphasize our molecular docking findings and to point out the structural features of the active compounds, a ligand-based pharmacophoric study using the currently available anti-toxoplasmosis drugs i.e., sulfadiazine ‘sulfonamide’, pyrimethamine, atovaquone, spiramicyne, clindamycine and epiroprim^5,6^, were set to build a pharmacophoric model using fixable alignment approach, with which, we got the common structural features (Fig. 8) including; F1: hydrophobic region/aromatic ring center ‘Hyd/Aro’, F2: and F3: H-bond acceptor and Metal ligator ‘Acc&ML’.

After what, we performed a pharmacophoric search for all identified compounds (**1–41**) as ‘a test set’, this resulted with 38 hits representing compounds (**2**–**10**, **12–19**, **21**–**41**) which attained the characteristic pharmacophoric features. Briefly, from the most promising results we got the conclusion that, the presence of linear OH-groups as in compounds (**2**,** 3**) are able to form HB-Acc and/or ML, along with their side chain which accounted for their hydrophobicity. Moreover, it becomes mush bettered by the presence of a phenolic hydroxyl (**OH**), a carbonyl (C=**O**) and/or a methoxy group (**O**CH_3_) to attain HB-Acc and/or ML, besides, the side chain (–**CH**_**3**_) accounted for their hydrophobicity and/or the Arc-ring center as attained by compounds (**4**–**10**, **14**–**19**,** 21**,** 22**). Additionally, flavone and flavonol aglycones (**23**–**30**,** 35**) exhibited HB-Acc and/or ML via hydroxy methyl group (–**O**CH_3_) substituted on A-ring, and attained Hyd/Arc with C-ring system. However, flavanone aglycones (**37**,** 38**) displayed HB-Acc and/or ML through (–**O**– & C=**O**) groups of C-ring, while B-ring displayed Hyd/Arc. Finally, in case of phenolic, flavanone and flavonol glycosides compounds (**12**,** 13**,** 31**–**34**,** 36**,** 39**–**41**), all the pharmacophoric features **F1** (Hyd), **F2** & **F3** (Acc&ML) achieved mainly within the sugar moieties, while, in case of vitexin, rutin, isorhamnetin 3-glucoside, and isorhamnetin 3-neohesperidoside, the **F1**: Arc ring center appeared within A-, C-, B-, and C-ring system, respectively.

We can deduce that, our molecular docking findings are expressive for *C. limon* MeOH ext., containing compounds (**1**–**41**) having the power to affect the target proteins, in such a way to combat against the parasite, and an indicative for their potential anti-toxoplasmosis activity against *T. gondii* at its life stages. It noteworthy, that *C. limon* MeOH ext. exerts its anti-toxoplasmosis via multiple modes of action i.e., extracellular grown tachyzoites reduction and redox homeostasis disruption, morphological alterations and cell cycle disruption via iron chelation^81^. However, the most promising candidates mentioned above work together synergistically for acquiring their anti-toxoplasmosis activity.

### Molecular dynamic analysis

Proteins may experience conformational changes at different degrees to perform various cellular functions. Even small structural changes can impact the protein-ligand functionality. The dynamic behavior of a protein–ligand complex can be understanding via the extensive sampling of MD trajectory frames over the entire simulation period, in our study, from the frontrunner list (S_13), complexes 4JRN_lig-34, 2O2S_lig-36 and 4M84_lig-39 were selected based on the highest binding affinity and then evaluated for their conformational stability within the receptor’s binding pocket. For MD simulation period lasted for 100 ns, we examined protein-ligand RMSD, RMSF, RoG, SASA and H-bonding stability via cluster analysis.

### Root-mean square deviation

**Fig. 9:**
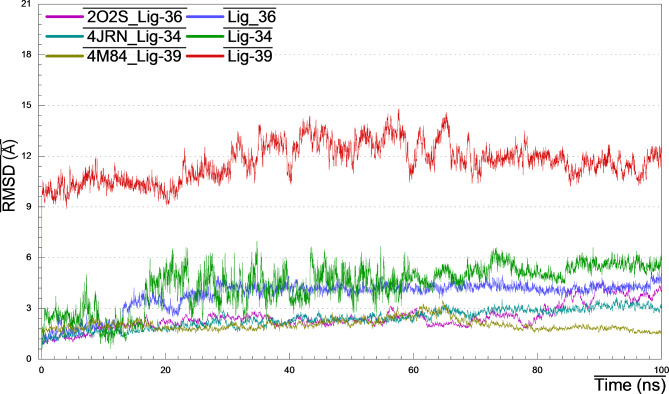
RMSD plots of 4JRN_lig34, 2O2S_lig-36 and 4M84_lig-39 during 100 ns MD simulation.

The average deviation in the displacement of a group of atoms with respect to a reference frame through a given frame, is defined as RMSD. As detected in (Fig. 9) that all complexes showing stable behavior throughout most of the 100 ns trajectory, maintained fluctuations within the range of 2–3 Å, and hence, are believed to have attained stability, convergence and conformational stability. On the other hand, lig-34 and − 36 fluctuated steadily during the first 18 ns with RMSD values of ~ 2.5 Å, then after, increased till 5–5.5 Å and held equilibrium. Surprisingly, and at the beginning of MD simulation, lig-39 fluctuated drastically with RMSD 10 ± 1.5 Å, and then kept equilibrium till the simulation ended.

### Root-mean square fluctuation

**Fig. 10:**
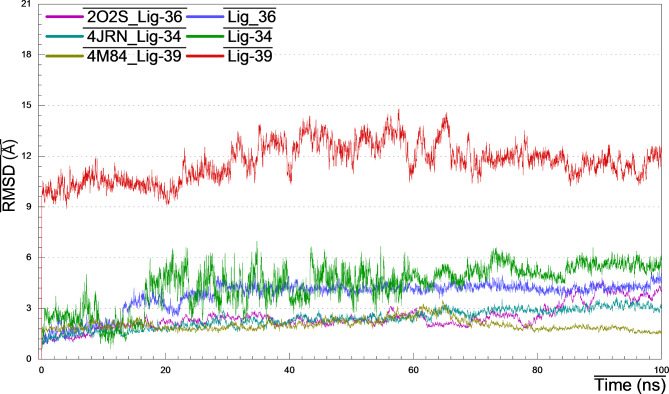
RMSF (Chain Cα) plots of 4JRN_lig34, 2O2S_lig-36 and 4M84_lig-39 during 100 ns MD simulation.

The distance between an atom and its average position in a given set of structures, is a determinant of structural flexibility, so, the higher stability of a protein-ligand complex is connected with the lower RMSF degrees. Keeping in mind that, loop regions with more conformational flexibility, where the structure is not well defined, preserve the higher RMSF values. As appeared in (Fig. 10) the core structural fold region of the three complexes showed the lowest RMSF values < 2 Å. Noteworthy that, 4M84_lig-39 complex recorded the least RMSF ~ 1.5 Å, however, the highest fluctuations with 6 Å was a record for complex 2O2S_lig-36 at residues 230–235, besides that, residues 25–30 and 255–265 of 4JRN_lig-34 complex fluctuated with ~ 3.5 Å. Notwithstanding, the resulted RMSF supports the concept that formed complexes are stable at the site each ligand binds to and do not significantly change the residue fluctuations at any regions.

### Radius of gyration (RoG)

**Fig. 11:**
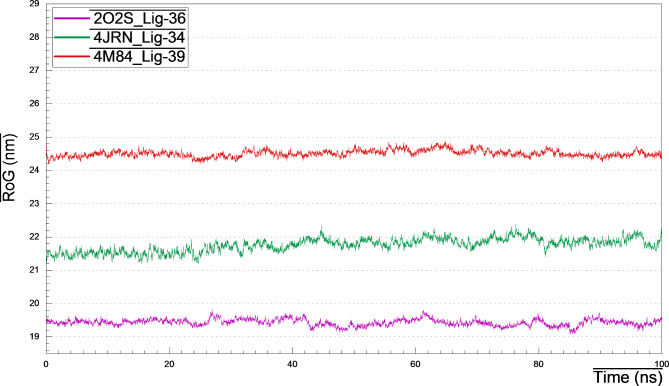
RoG plots of 4JRN_lig34, 2O2S_lig-36 and 4M84_lig-39 during 100 ns MD simulation.

The radius of gyration measurement is a determinant of the compactness of a protein structure, which reflects its stability. Hence, less stable structures are those with higher fluctuations and higher RoG. Over time span of 100 ns simulation (Fig. 11), the average RoG was ranged between 19.2 and 19.4 nm for 2O2S_lig-36 complex, 21.4–22.0 nm for 4JRN_lig-34 complex, and 24.2–24.6 nm for 4M84_lig-39 complex. The lower values of RoG indicated that, the ligand binding to the protein’s active site does not induce major conformational changes in the protein structure, and accounted for their stability.

### Solvent accessibility surface area (SASA)

**Fig. 12:**
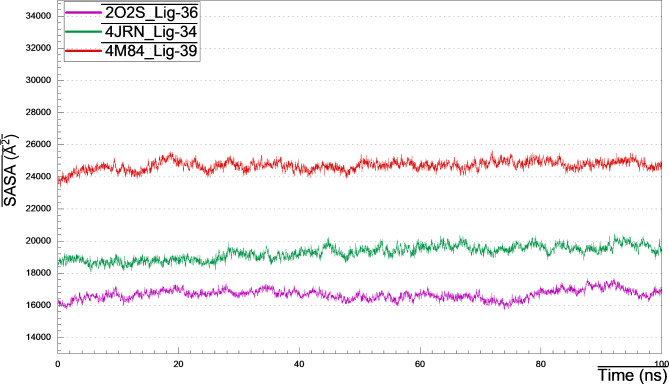
SASA plots of 4JRN_lig34, 2O2S_lig-36 and 4M84_lig-39 during 100 ns MD simulation.

The influence of the surrounding solvent on the protein-ligand complex, is known as SASA, which is a fundamental and pivotal parameter in complex structure analysis, and affects consequently on the rate of absorption and metabolism of a drug candidate. It provides insights into the protein’s folding, interactions with other molecules and stability. As observed in (Fig. 12), the SASA values (Å^2^) against time for all complexes over the entire simulation, are within the acceptable range, the thing that reflected their folding, stability and compactness, which unveiled the effectless of ligand binding to the protein’s active pocket.

### H-bonding behavior via cluster analysis

#### 2O2S_lig-36_complex

**Fig. 13:**
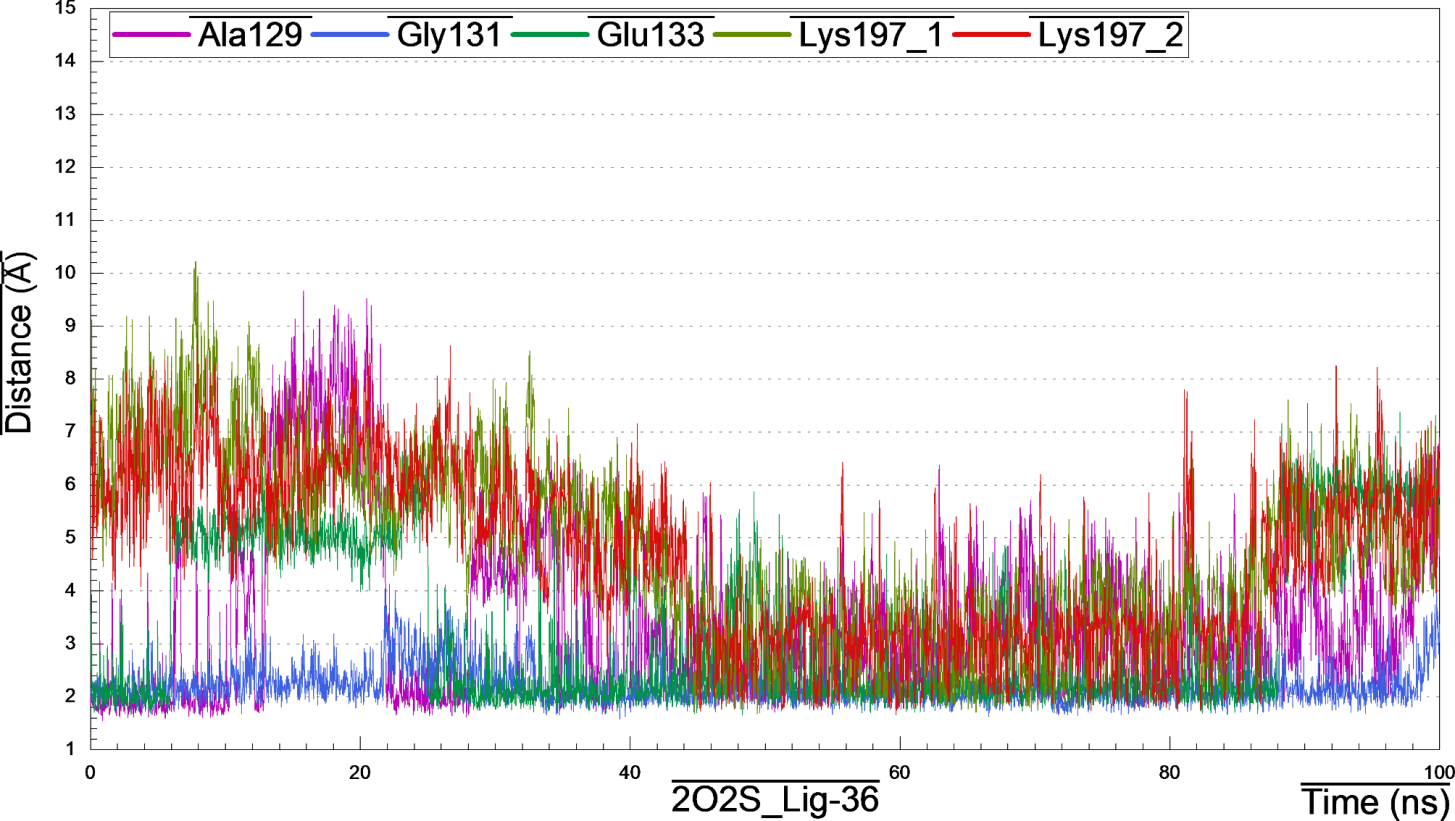
Distance plot of atom pairs involved in the formation of hydrogen bonds between lig-36 and Ala129, Gly131, Glu133 and Tyr197 in the course of MD simulation trajectory.

Visual inspection of the structural pose of 2O2S_lig-36 complex obtained from docking analysis (S_13) demonstrated that, Lig-36 might form hydrogen bonds with key amino acids like Asp19, Tyr189 and Lys197. However, MD simulation cluster analysis indicated that, the most representative pose owned H-bonding with Ala129, Gly131, Glu133 and Lys197 (Fig. 13), the thing that unveil the instability of the above-mentioned H-bonds from docking analysis.

**Fig. 14:**
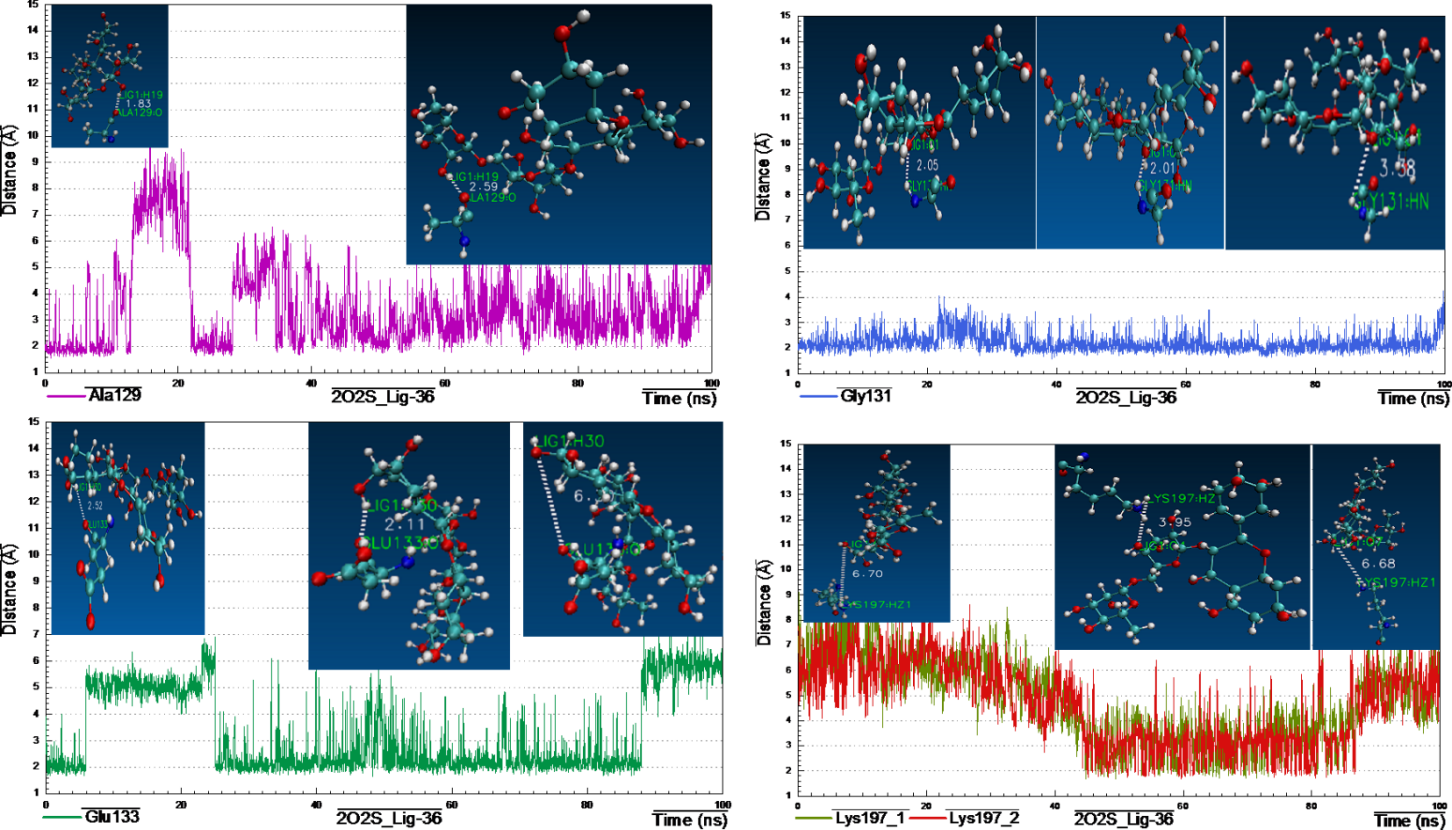
Distance plots of atom pairs involved in the formation of hydrogen bond between lig-36 and Ala129, Gly131, Glu133 and Lys197 as extracted by cluster analysis during the course of MD simulation trajectory.

Briefly, after monitoring and plotting the H-bond distance between lig-36 and amino acids Ala129, Gly131, Glu133 and Lys197, it appeared clearly that, the hydroxyl hydrogen at the backbone of Ala129 form stable H-bond with the hydroxyl oxygen of the hexose sugar of lig-36 with distance 1.83 Å for the first 10 ns and then began to fluctuates between 10 and 35 ns, then after, acquired stability with bond distance 2.59–3.0 Å (Fig. 14). Interestingly, the H-bond formed between amino-hydrogen of Gly131 and the oxygen atom of hexose moiety of lig-36, was strong and very stable throughout the MD simulation with distance 2.05–3.08 Å. However, H-bond formed between lig-36 and Glu133 was stable only between 25 and 95 ns with distance 2.11 Å. Surprisingly, H-bond formed between Lys197 and lig-36, as appeared in docking analysis and confirmed from cluster analysis, was not stable enough as it fluctuated at the beginning of simulation for the first 45 ns (~ 6.7 Å), and the came to stability with distance 3.95 Å within 45–90 ns, then after, it fluctuated with distance 6.68 Å.

#### 4JRN_lig34_complex

**Fig. 15:**
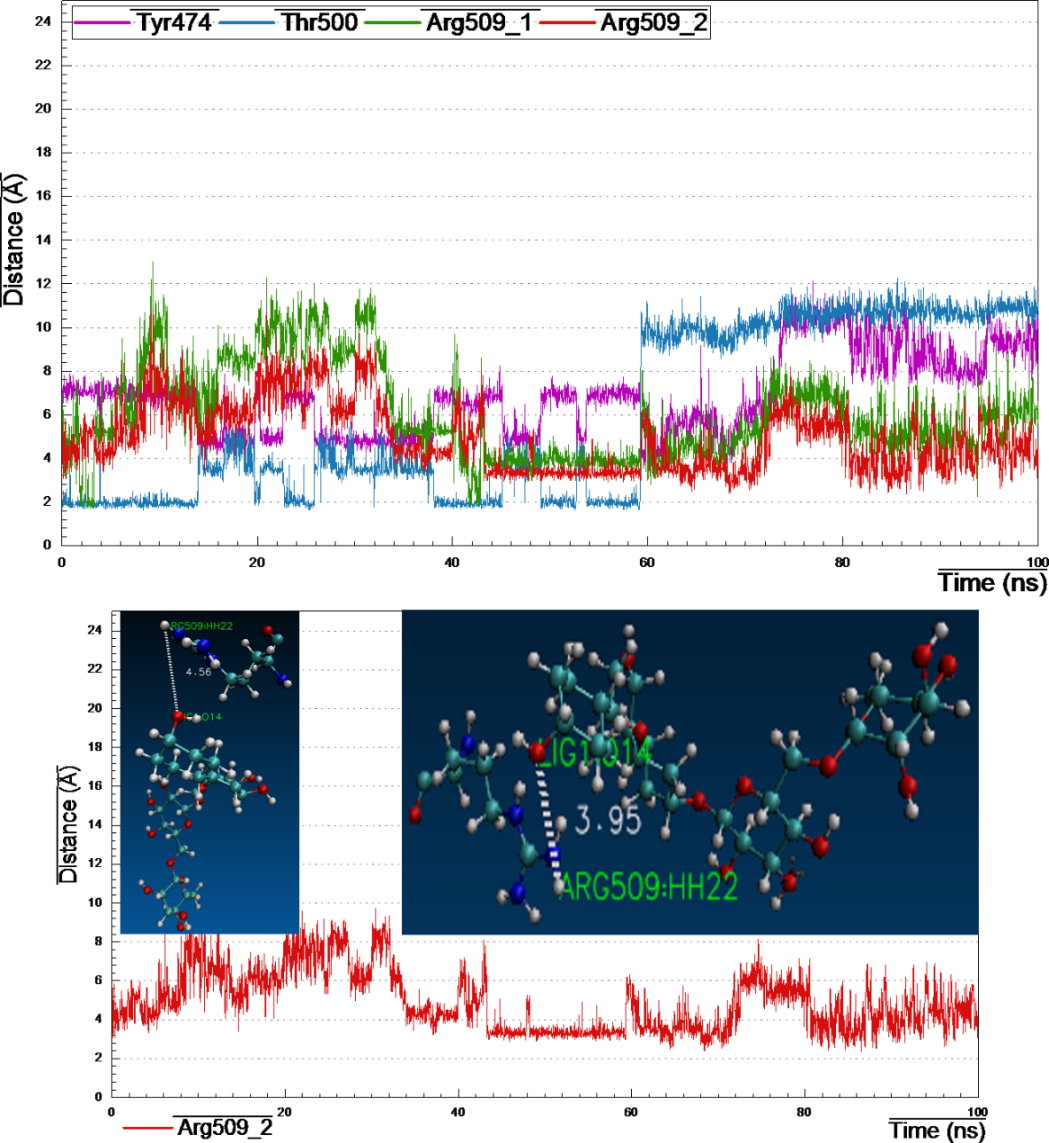
Distance plot of atom pairs involved in the formation of hydrogen bonds between lig-34 and Tyr474, Thr500 and Arg509 in the course of MD simulation trajectory.

Retrieving the docking result of 4JRN_lig34 complex (S_13), showed three H-bonds between lig-34 and amino acids like Lys281, Meth356 and Asp427, however, these bonds were instable enough according to the cluster analysis (Fig. 15), that recognized three H-bonds with Tyr474, Thr500 and Arg509. Furthermore, distance measurements revealed that the two H-bonds formed between the terminal amino-hydrogens of Arg509 backbone and the p-hydroxyl-oxygen at the B-ring of lig-34 were relatively stable with bond distance 3.95 Å, during the most of simulation time.

#### 4M84_lig39_complex

**Fig. 16:**
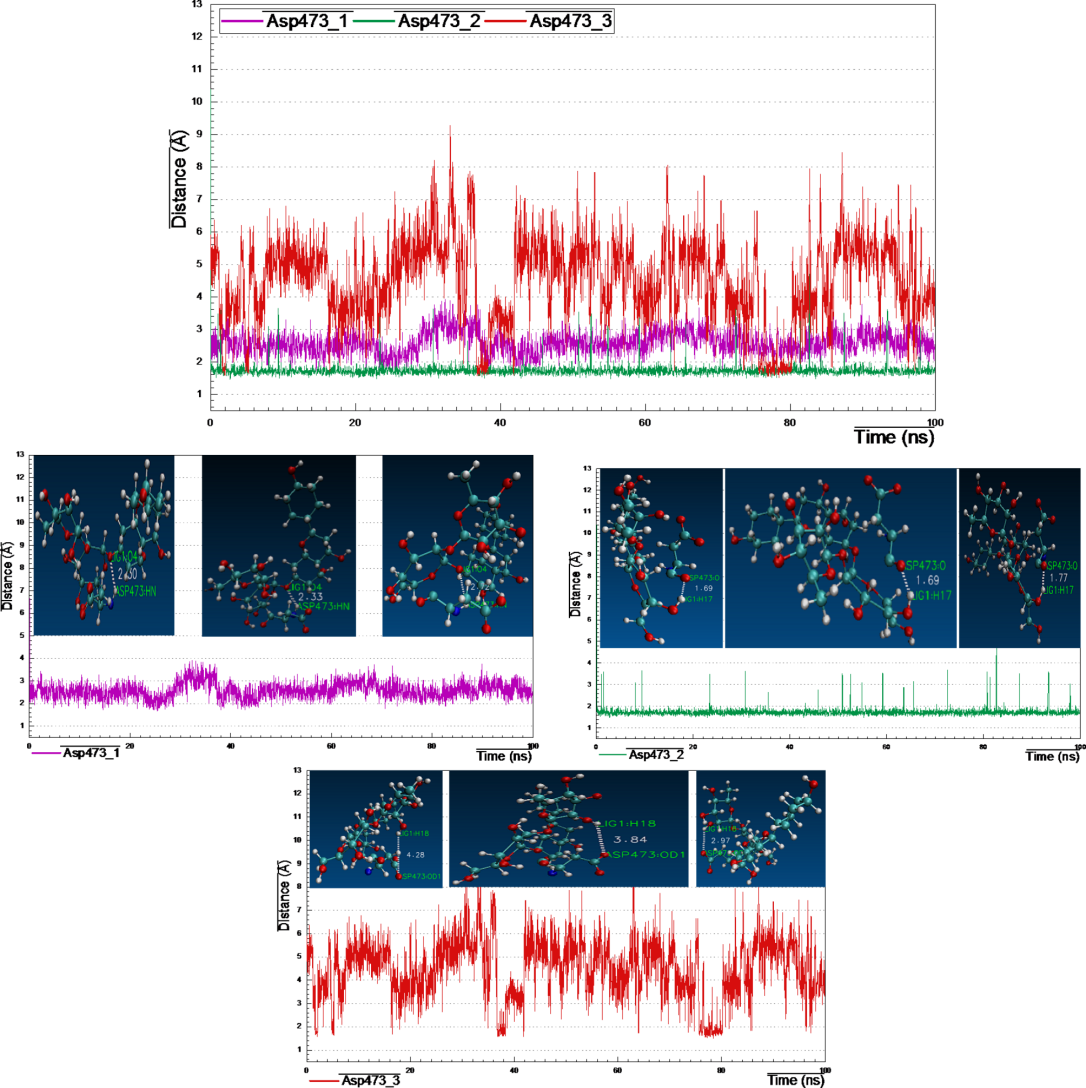
Distance plot of atom pairs involved in the formation of hydrogen bonds between lig-39 and Asp473 in the course of MD simulation trajectory.

Reclaiming the docking results of 4M84_lig-39_complex (S_13), a complex network of H-bonds was established with amino acids like Leu57, Lys59, Tyr131 Glu178, Asp195 and Lys338. Surprisingly, by monitoring cluster analysis of the MD trajectory, we realized that none of the above-mentioned H-bonds were stable enough throughout the entire simulation period (Fig. 16).

Noteworthy that, only one amino acid Asp473 (amino-hydrogen and carbonyl-oxygen atoms) revealed three H-bonds with lig-39 (sugar moieties), conducting distance analysis measurements revealed very strong and stable H-bond formed between carbonyl-oxygen of the side chain of Asp473 and the of the 4-hydroxyl-hydrogen of the glucopyranosyl moiety with ~ 1.70 Å during the MD simulation time, one more strong H-bond formed between the amino-hydrogen of Asp473 and the oxygen atom at position-7 of the A-ring of lig-39, in addition, the third H-bond between the terminal carbonyl-oxygen of Asp473 and the 2-hydroxyl-hydrogen at the rhamnopyranosyl moiety.

#### ADMET predictions

The prediction of the ADMET properties ‘the pharmacokinetic factors’ of a candidate drug is necessary for enzyme induction or inhibition. Absorption, distribution, metabolism, excretion and toxicity (ADMET) proprieties for all characterized compounds (**1–41**) are appeared in (**S_14 & S_15**).

Concisely, as known ‘Lipinski rules of 5’ are not fitted for complex natural products^106^, however, candidates (**1**–**20**, **23**–**30**, **35**, **37**, **38**) did not violate any rule, and therefor considered to be orally active, however, compounds (**34**, **36**, **39**, **41**) gave three violations, two violations were observed for compounds (**21**, **33**, **40**), however, compounds (**22**, **31**, **32**) recorded one violation, hence, it can be expected to be orally inactive (**S_14**). Additionally, the Log *P*_*o/w*_ parameter (for drug potency and distribution in the body after absorption) was predicted, and the obtained values (– 0.59 to + 4.95) reflected good permeability for most of the compounds through the cell membrane, except for compounds **2**, **3** and **14** which showed values out of the range with − 0.79, -4.78 and 5.08 respectively. In addition, good candidates should be water-soluble compounds to be easily formulated and administrated orally, all the tested compounds revealed acceptable predictions of log *s*, with very soluble (**1**–**8**,** 12**,** 13**), soluble (**9**–**11**, **15**–**17**, **19**, **22**, **27**, **31**–**33**, **35**, **36**, **41**) and of moderate solubility for (**14**, **18**, **20**, **21**, **23**–**26**, **28**–**30**, **34**, **37**–**40**) along with the control (**S_14**).

TPSA parameter was chosen to demonstrate the transportation (absorption and passive diffusion) through biological barriers, results from (**S_14**) uncovered that, compound **20** represented the best candidate with TPSA value of 26.30 Å^2^, together with compounds (**1**, **4**–**11**, **14**–**19**, **22**–**25**, **28**–**30**, **37**, **38**) with TPSA range (37.30–127.45 Å^2^), however, compounds (**2**, **3**, **12**, **13**, **21**, **26**, **27**, **31**–**36**, **39**–**41**) displayed relatively high TPSA values implying poor absorption through cell membrane.

Drug discovery demands the study of both pharmacokinetics and pharmacological activities of new candidates, as a consequence, the suitability of an oral drug contender can be determined by its human intestinal absorption (HIA) record^107^, therefore, we can declared from (**S_14**) that, compound **20** is the prime candidate with HIA of 100%, moreover, most of the tested compounds (**1**, **4**, **10**, **11**, **14**, **15**, **17**–**19**, **21**, **23**–**26**, **28**–**30**) and (**7**, **9**, **16**, **37**, **38**) recorded an outstanding HIA values of (90.60–99.07%) and (82.03–88.20%) respectively, and even better than spiramycin with 82.17%, which reflects good oral absorption, whereas, the rest compounds (**2**, **3**, **5**, **6**, **8**, **12**, **13**, **22**, **27**, **31**–**36**, **39**–**41**) showed poor absorption.

To support oral absorption predictions, the trustworthy in vitro models (P_Caco2_ & P_MDCK_) for rapid screening permeability^108^, have been predicted as listed in (**S_14**), compounds (**17**, **18**, **23**–**25**, **28**, **29**) gave the best permeability better than spiramycin with P_Caco2_ (43.20–55.25) and 39.97 nm/s respectively. Whilst, compounds (**1**, **3**, **4**, **6**–**8**, **9**–**11**, **14**, **16**, **22**) showed acceptable P_Caco2_ values > 15 nm/s. In addition, the predicted P_MDCK_ values for all compounds (**1**–**41**) were much better than that of spiramycin of 0.04 nm/s, in other words, compound **10** have the highest permeability with 228.56 nm/s, followed by compounds (**20** and **9**) with P_MDCK_ values of 146.33 and 109.43 nm/s respectively, moreover, compounds (**1**, **4**, **7**, **8**, **12**, **14**–**19**, **27**, **37**, **38**) recorded P_MDCK_ values (23.76–78.43) nm/s, while the rest compounds showed lowest permeability. Additionally, all the tested compounds (**1**–**41**) gave negative values for skin permeability P_Skin_, explained their improper utility for transdermal use with no risk present.

Plasma protein binding (PPB) affinity is a prime objective to study both drug distribution and partitioning within tissues (i.e., adipose tissue), as appeared in (**S_15**), three compounds (**14**, **20**, **38**) scored the potent PPB affinity with 100%, whereas compounds (**6**, **17**–**19**, **21**, **23**–**25**, **27**–**30**, **35**, **37**) displayed higher PPB strength ranged (84.86–99.72%), other compounds (**4**, **5**, **7**, **9**–**13**, **22**, **26**, **31**–**34**, **36**, **39**–**41**) exhibited satisfactory PPB capacity > 30%.

Penetration of blood brain barrier (BBB) by drug candidates can critically harm CNS and may lead to neurotoxicity, however, out of the frontrunner list (**S_15**) only compound (**14**) showed a relatively high penetration value with 8.82, others (**1**, **17**–**20**) showed values ranged from (1.15–2.07), while the rest of the list, having very low C_Brain_/C_Blood_ penetration values (< 1) ranged between (0.01–0.76), proving their poor capability to pass through the BBB and/or affect the CNS, and so classified as inactive^109^. Moreover, Finch and Pillans^110^, documented that, P-glycoprotein is an important efflux pump for drug distribution and transportation, so as listed in (**S_15**), out of the list, only compounds (**14**, **19**–**22**, **24**, **25**, **29**) recorded inhibition for P-gp, which may affect its protection against xenobiotics and drug bioavailability.

Cytochrome P450 as one of CYP enzymes family includes different isoforms (i.e., 2C6, 3A4, …etc.) which mainly distributed in liver and intestine, Lynch and Price^111^ studied their effect on drug metabolism (interactions, response and adverse effects), and concluded that, for a target candidate to be involved in drug-drug interactions it should inhibits one or more CYP isoforms. Two modes of inhibition effects of natural derived metabolites: competitive or non-competitive (reversible inhibition) and mechanism-based inhibition ‘MBI’ concerning with drug metabolism by CYP450s into reactive metabolites bind rigidly to the active site of an enzyme (irreversible inhibition) namely ‘suicide inhibition’^112^. The obtained predictions (**S_15**) for our ligands (**1**–**41**) revealed negative outcomes for CYP2D6, however, the predicted results for CYP3A4 were varied with positive effect as inhibitors for most tested compounds including (**1**, **2**–**6**, **8**–**10**, **12**–**14**, **16**, **17**, **21**–**41**), and negative effect for compounds (**2**, **7**, **11**, **15**, **18**–**20**), compared to positive effect for spiramicyne.

Additionally, before drug candidate undergo clinical trial, the in-silico toxicity measurement is an important procedure, the toxicological predictions (mutagenicity and carcinogenicity) of our targets were characterized (**S_15**), and portended that, compounds (**1**–**11**, **15**–**19**, **23**–**30**, **35**–**41**) were not carcinogenic in mouse only, however, compounds (**12**, **14**, **31**–**33**) and spiramycin recorded positive carcinogenicity in mouse and negative in rat, moreover, compounds (**13**, **22**, **34**) were predicted as carcinogenic in both models, finally, compounds (**20** & **21**) predicted as safe with no carcinogenicity for both mouse and rat, on the other hand, spiramycin and compounds (**14**, **21**, **22**, **31**, **33**, **34**, **36**, **39**–**41**) gave negative predictions and classified as non-mutagenic, whereas, the rest of the list (**1**–**13**, **15**–**20**, **23**–**30**, **32**, **35**, **37**, **38**) considered mutagenic with positive prediction (**S_15**). It is very useful to determine the drug half-life (**t**_½_) as indicator for how fast a drug is removed from the body, the shorter half-life the larger of its total clearance, as predicted in (**S_15**) all compounds revealed relatively long half-life **t**_½_ compared to Spi.

Finally, for a certain target to be highly selected as a drug candidate it should possess a high drug score value, out of the frontrunner list, compound (**39**) wined the best score with value of 1.05, besides, compounds (**8**, **22**, **24**, **25**, **27**–**38**, **40**, **41**) showed values ranged (0.18–0.91), the worst score values were listed from compounds (**1**–**7**, **9**–**21**, **23**, **26**).

## Conclusion

We can acclaim from the obtained results, that, *C. limon* MeOH ext., together with the isolated bergapten, vitexin and isovitexin, work synergistically, to exert their anti-toxoplasmosis activity via one or more of the following mechanisms; blocking the cytoplasmic membrane function, preventing bacterial virulence factors, the suppression of energy metabolism and nucleic acid synthesis. Besides that, they can stimulate natural killer cells and macrophages, phagocytosis, regulation of cytokine excretion and production of immunoglobulins.

## Electronic supplementary material

Below is the link to the electronic supplementary material.

Supplementary Material 1

## Data Availability

Availability of data and materialsAll data generated or analyzed during this study are included in this published article and its supplementary information files.
