# Publisher Correction: Discovery of potent anti-toxoplasmosis drugs from secondary metabolites in *Citrus limon* (lemon) leaves, supported in-silico study

**DOI:** 10.1038/s41598-025-89352-y

**Published:** 2025-02-18

**Authors:** Magdy Mostafa Desoky Mohammed, Hala Sh. Mohammed, Salwa A. Abu El Wafa, Doaa A. Ahmed, Elham A. Heikal, Islam Elgohary, Ashraf M. Barakat

**Affiliations:** 1 Pharmacognosy Department, Pharmaceutical and Drug Industries Research Institute, National Research Centre, Dokki, Giza, 12622 Egypt; 2 Pharmacognosy and Medicinal Plants Department, Faculty of Pharmacy (Girls), Al-Azhar University, Cairo, Egypt; 3 Medical Parasitology Department, Faculty of Medicine, Al-Azhar University for Girls, Cairo, Egypt; 4 Department of Pathology, Agriculture Research Centre, Animal Health Research Institute, Dokki, Giza, Egypt; 5 Department of Zoonotic Diseases, National Research Centre, Dokki, Giza, 12622 Egypt

Correction to: *Scientific Reports* 10.1038/s41598-024-82787-9, published online 03 January 2025

The original version of this Article contained an error in Figure 1, where the column “R^7^” and its respective data was omitted. The original Figure 1 and accompanying legend appear below.

**Fig. 1:**
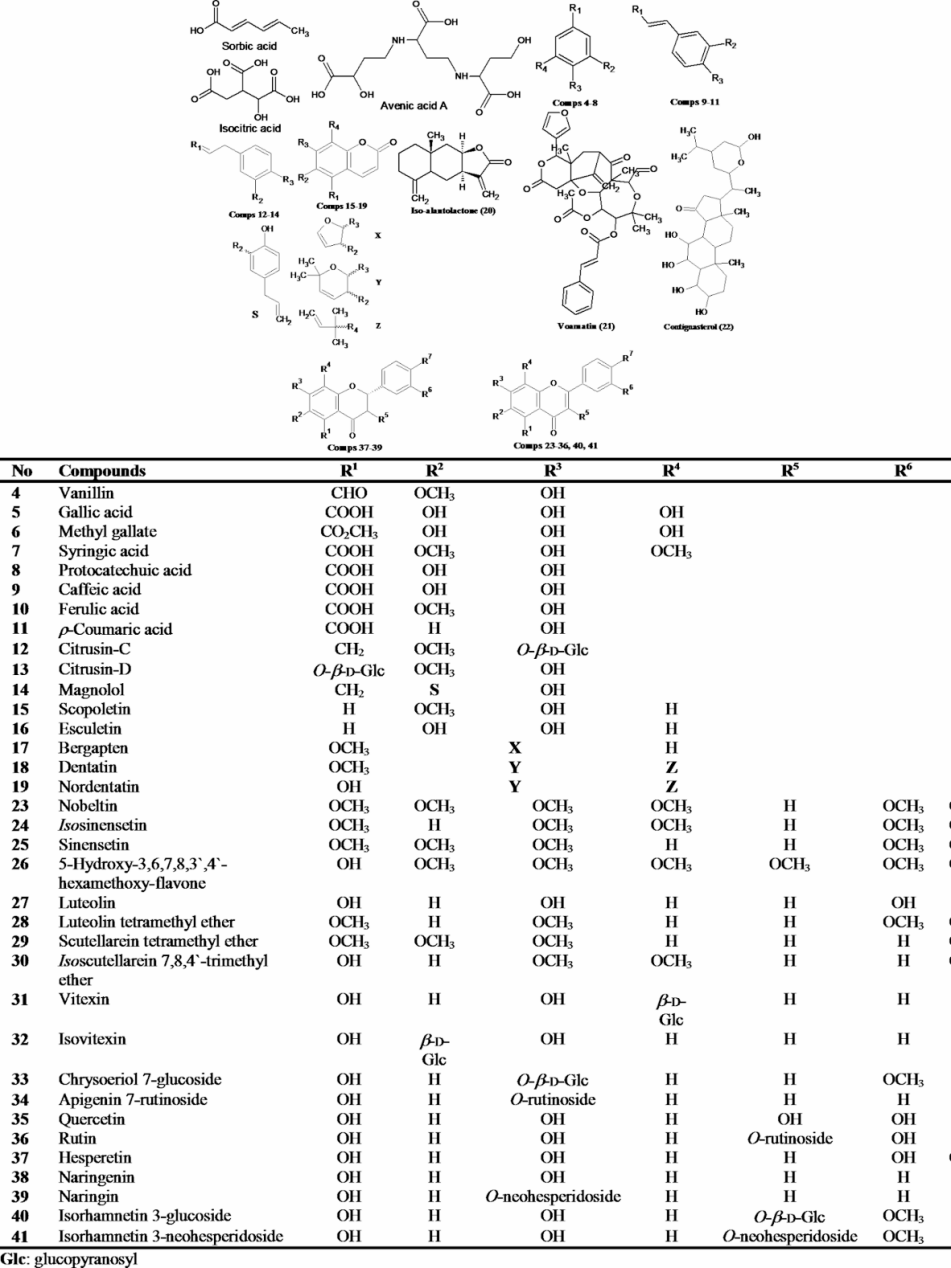
The identified metabolites from *C. limon* leaves MeOH ext.

The original Article has been corrected.

