# Supplementary material for: Discovery of potent anti-toxoplasmosis drugs from secondary metabolites in Citrus limon (lemon) leaves, supported in-silico study
Source: Sci Rep. 2025 Jan 3;15:624. doi: 10.1038/s41598-024-82787-9 (PMC11698829; doi:10.1038/s41598-024-82787-9)

**Discovery of Potent Anti-Toxoplasmosis Drugs from Secondary Metabolites in**

***Citrus limon* (Lemon) Leaves, Supported *in-Silico* Study**

**Magdy M. D. Mohammed^1,*^, Hala Sh. Mohammed^2,*^, Salwa A. Abu El Wafa^2^, Doaa A. Ahmed^3^, Elham A. Heikal^3^, Islam Elgohary^4^, Ashraf M. Barakat^5^**

^1^Pharmacognosy Department, Pharmaceutical and Drug Industries Research Institute, National Research Centre, Dokki-12622, Giza, Egypt.

^2^Pharmacognosy and Medicinal Plants Department, Faculty of Pharmacy (Girls), Al-Azhar University, Cairo, Egypt.

^3^Medical Parasitology Department, Faculty of Medicine, Al-Azhar University for Girls, Cairo, Egypt.

^4^Department of Pathology, Animal Health Research Institute, Agriculture Research Centre, Dokki, Giza, Egypt.

^5^Department of Zoonotic Diseases, National Research Centre, Dokki-12622, Giza, Egypt.

Toxoplasmosis induced by *Toxoplasma gondii* is a well-known health threat, that prompts fatal encephalitis increased with immunocompromised patients, in addition, it can cause chorioretinitis, microcephaly, stillbirth in the fetus and even led to death. Standard therapy uses sulfadiazine and pyrimethamine drugs revealed beneﬁcial results during the acute stage, however, it has severe side effects. UPLC-ESI-MS/MS used to explore *C. limon* MeOH ext. constituents, which revealed a list of **41** metabolites of different classes encompasses; unsaturated fatty acid, tricarboxylic acids, phenolic aldehyde, phenolic acids, phenolic glycosides, coumarins, sesquiterpene lactone, limonoid, steroid and flavonoids. *C. limon* MeOH ext. and the isolates reduced significantly the number of *T. gondii* tachyzoites. Consequently, histopathological examination, proved significant reduction in the number of mononuclear inflammatory cells in the kidney and liver sections, besides, lowering the number of shrunken and degenerative neurons in the brain sections of infected mice. Molecular docking study was performed targeted certain receptors, which are important for the life cycle fundamentals for the parasite mobility including invasion and egress, along with a pharmacophoric mapping confirmed the need for a free hydroxyl group and/or a phenolic substituted one, in order to form HB, Hyd/Aro and ML interactions, through which, cell cycle disruption via iron chelation, could be achieved. In addition, the ADMIT properties of all identified metabolites were predicted.

**Keywords:** *Citrus limon*, Rutaceae, *Toxoplasma gondii*, UPLC-ESI-MS/MS, Molecular Docking, Molecular Dynamics Simulation.

**S-1.** The Co-crystallized ligands, key amino acid residues, the grid box parameters and size dimensions

**S-2.** ^1^H-NMR spectra of compound C1, 400 MHz, (DMSO-*d_6_*)

**S-3.** ^13^C-NMR spectra of compound C1, 100 MHz, (DMSO-*d_6_*)

**S-4.** ^1^H-NMR spectra of compound C2, 400 MHz, (DMSO-*d_6_*)

**S-5.** ^13^C-NMR spectra of compound C2, 100 MHz, (DMSO-*d_6_*)

**S-6.** ^1^H-NMR spectra of compound C1, 400 MHz, (DMSO-*d_6_*)

**S-7.** ^13^C-NMR spectra of compound C2, 100 MHz, (DMSO-*d_6_*)

**S-8.** Base peak chromatogram (BPC) of *C. lemon* MeOH extract

**S-9.** Extracted ion chromatogram of authentic samples

**S-10.** TIC of authentic samples

**S-11.** Extracted ion chromatogram of *C. lemon* MeOH extract

**S-12.** TIC of *C. Lemon* MeOH extract

**S-13.** Docking scores and receptors amino acids involved in the interactions with the ligand compounds

**S-14.** Pharmacokinetics predictions of all compounds computed by SwissADME and PreADMET

**S-15.** Pharmacokinetics predictions of all compounds computed by SwissADME and PreADMET

**S-1. The Co-crystallized ligands, key amino acid residues, the grid box parameters and size dimensions**

| **PDB IDs** | **Grid box parameters**  **Center (x y z) /**  **Dimension (Å) (x y z)** | **Co-crystallized Ligands** |
| --- | --- | --- |
| ***Tg*ROP18**  **(4JRN)** | 4.98 43.83 23.86 /  19.39 21.89 23.86 | beta-D-fructofuranose-(2-1)-alpha-D-glucopyranose |
| ***Tg*ROP5**  **(3Q5Z)** | -9.29 -18.61 18.06 /  18.86 15.28 17.83 | 1,2-ethanediol |
| ***Tg*CDPK1**  **(4M84)** | 17.89 13.95 61.83 /  17.04 19.02 18.74 | 5-amino-1-tert-butyl-3-(quinolin-2-yl)-1H-pyrazole-4-carboxamide |
| **UPRTase**  **(1UPF)** | 12.22 97.09 48.42 /  13.76 20.88 17.12 | 5-fluorouracil |
| **AK**  **(1LIJ)** | 137.76 25.17 -5.93 /  14.88 21.00 18.77 | 2-ribofuranosyl-3-iodo-2,3-dihydro-1h-pyrazolo[3,4-d]pyrimidin-4-ylamine |
| **3MB8** | 50.05 30.45 59.89 /  12.86 16.79 13.07 | 1,4-dideoxy-4-aza-1-(s)-(9-deazahypoxanthin-9-yl)-d-ribitol |
| ***Ts*-DHFR**  **(4KY4)** | -12.95 -15.55 -45.87 /  17.54 17.69 15.96 | 2-amino-5-(phenylsulfanyl)-3,9-dihydro-4H-pyrimido[4,5-b]indol-4-one |
| **2O2S** | 25.01 46.90 40.25 /  13.76 24.17 15.11 | triclosan |
| **6A88** | 15.49 -1.35 26.06 /  20.92 13.86 15.16 | 3-{3-[(2R,3S)-3-hydroxypiperidin-2-yl]-2-oxopropyl}quinazolin-4(3H)-one |
| ***Tg*-MMIF**  **(4DH4)** | 17.67 6.46 24.98 /  16.30 17.27 15.96 | sulfate ion |

**S-2**


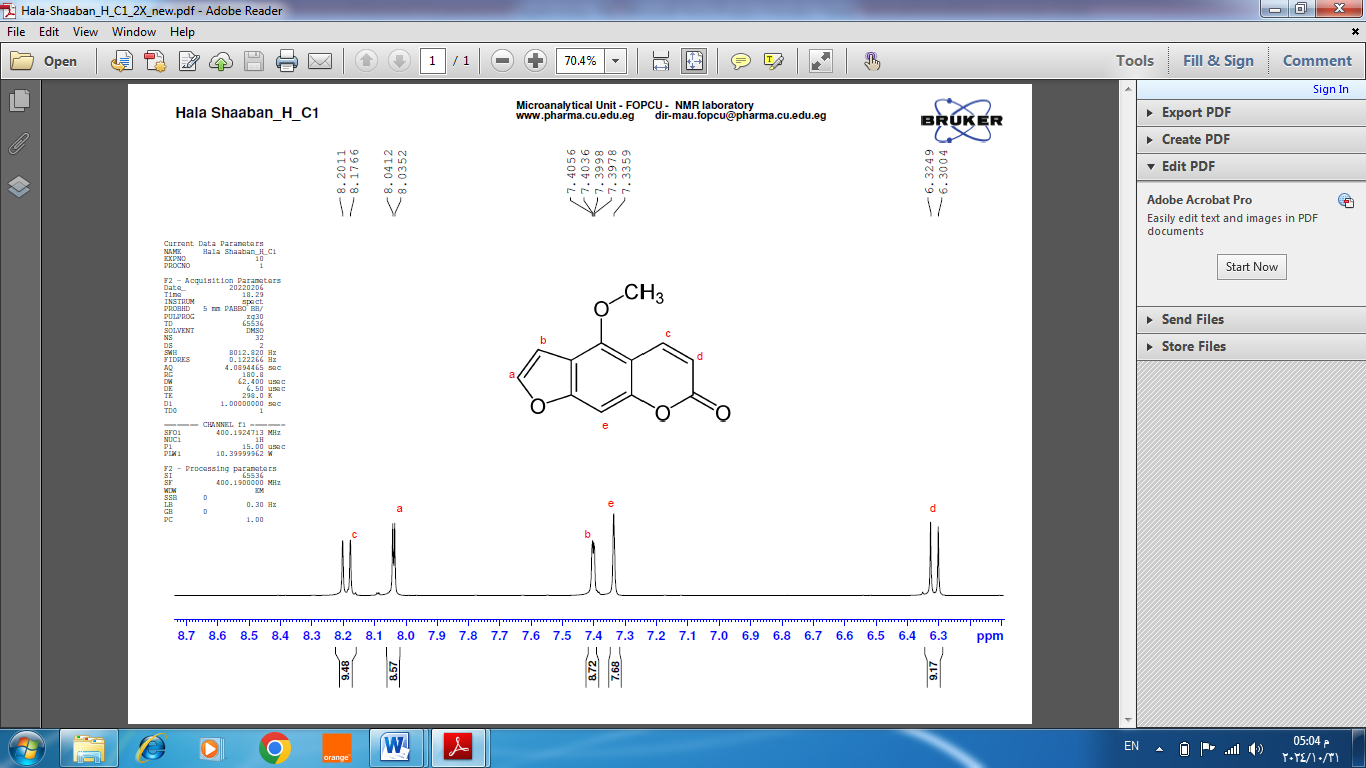


**S-3**


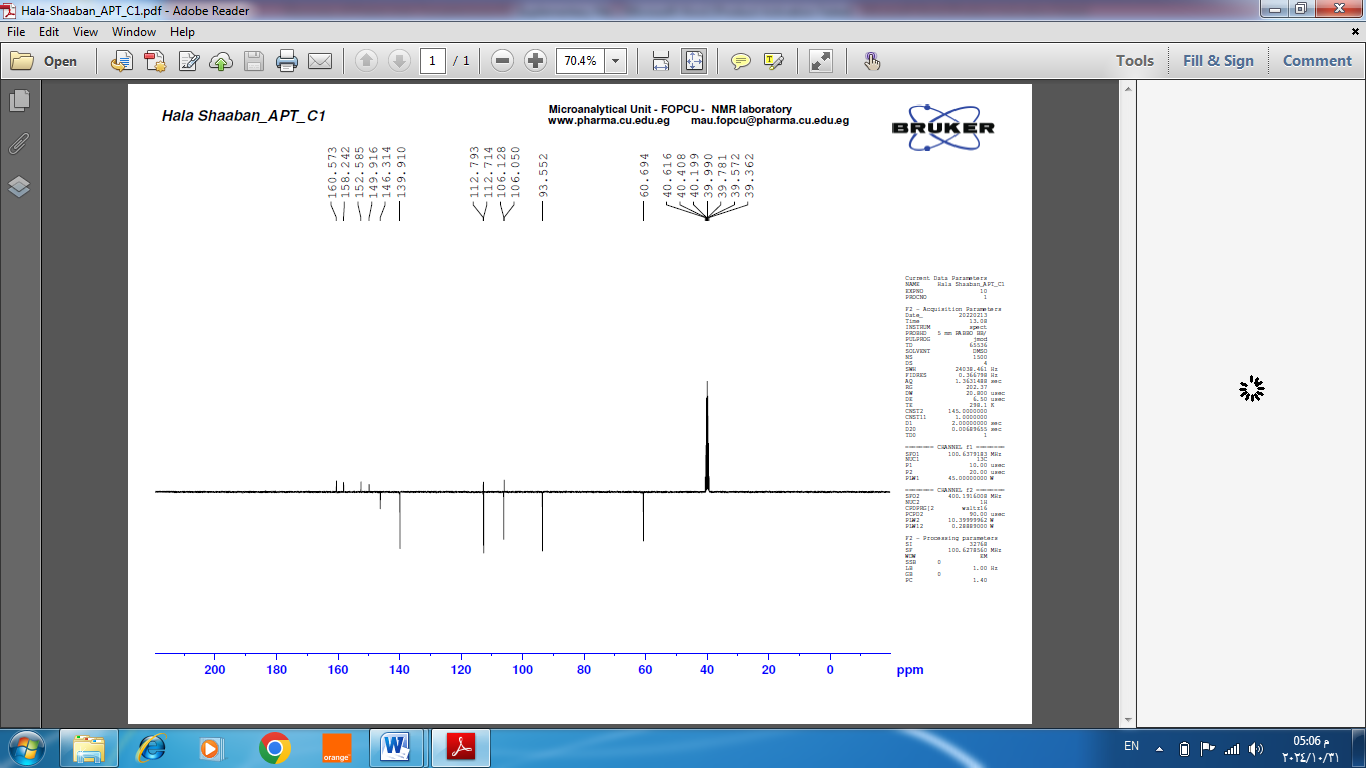


**S-4**


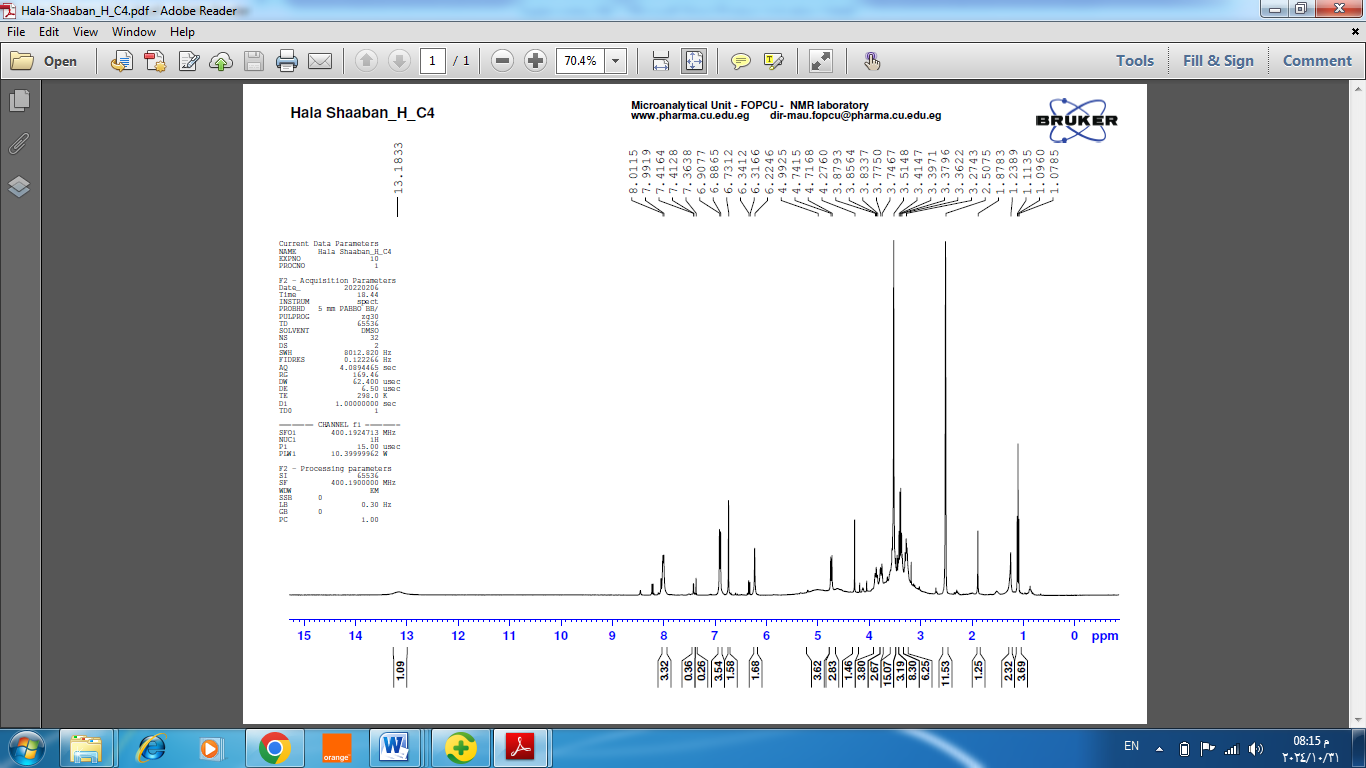


**S-5**


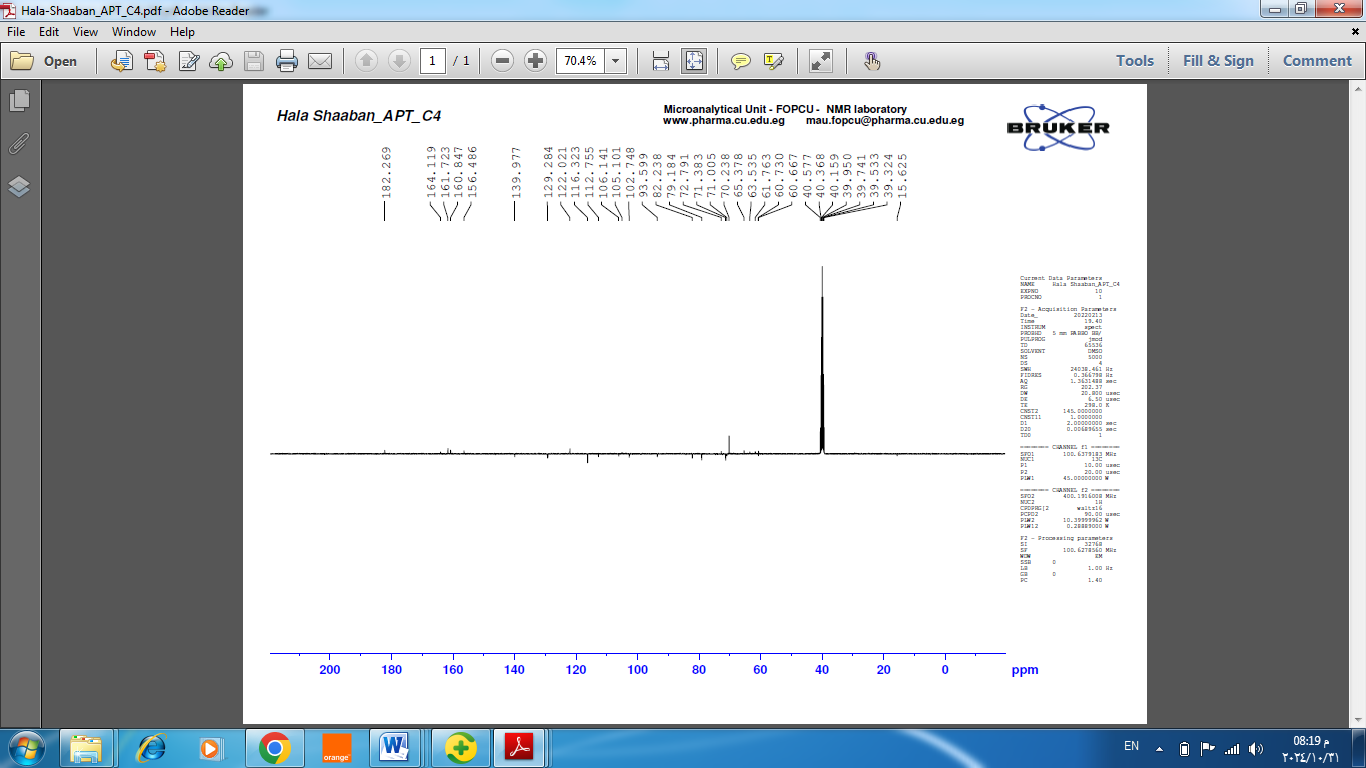


**S-6**


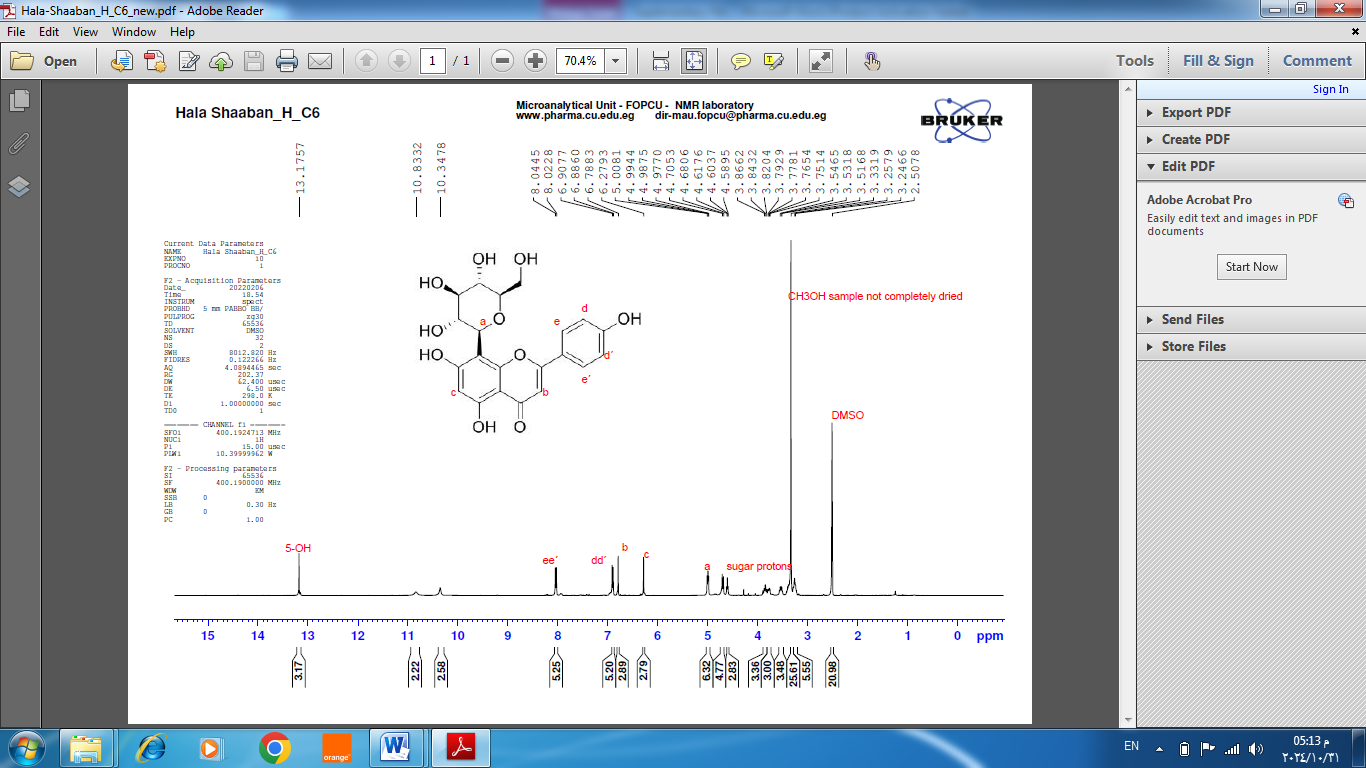


**S-7**


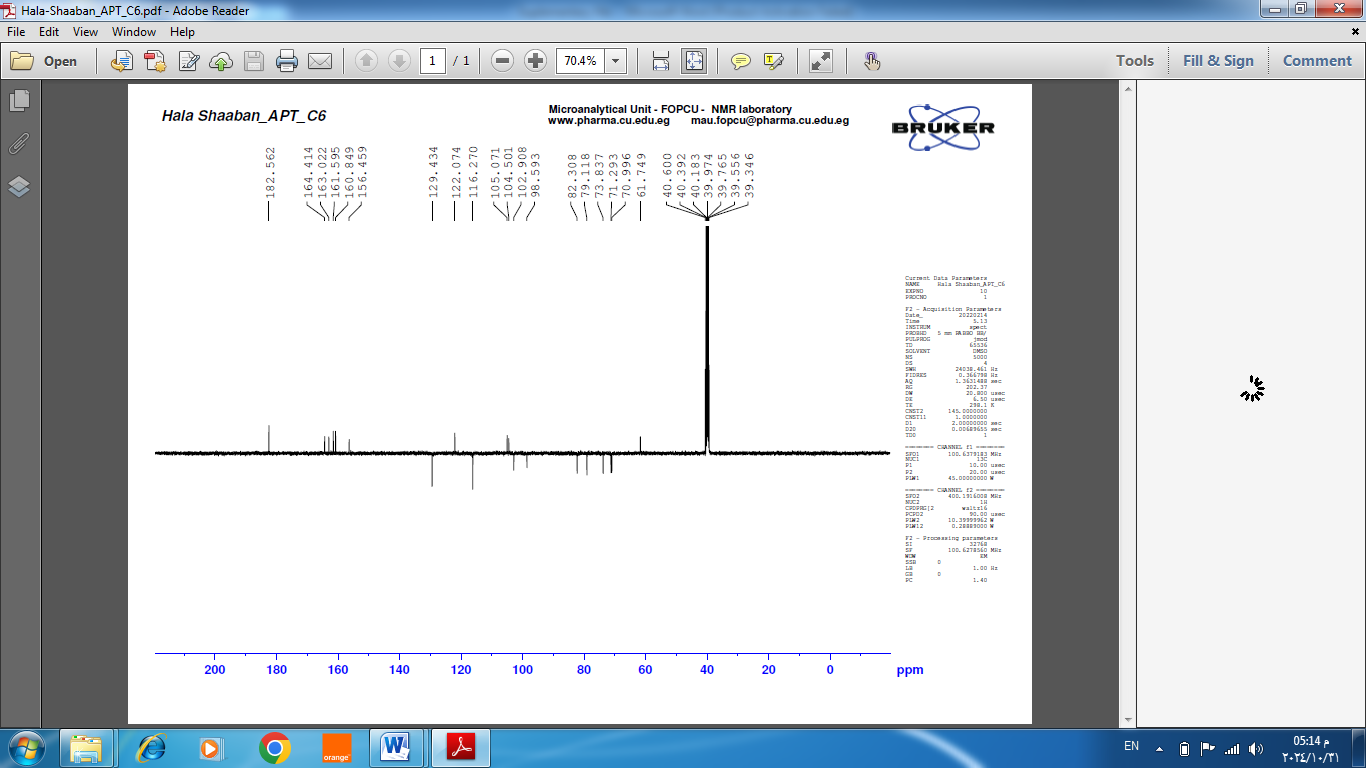


**S-8**


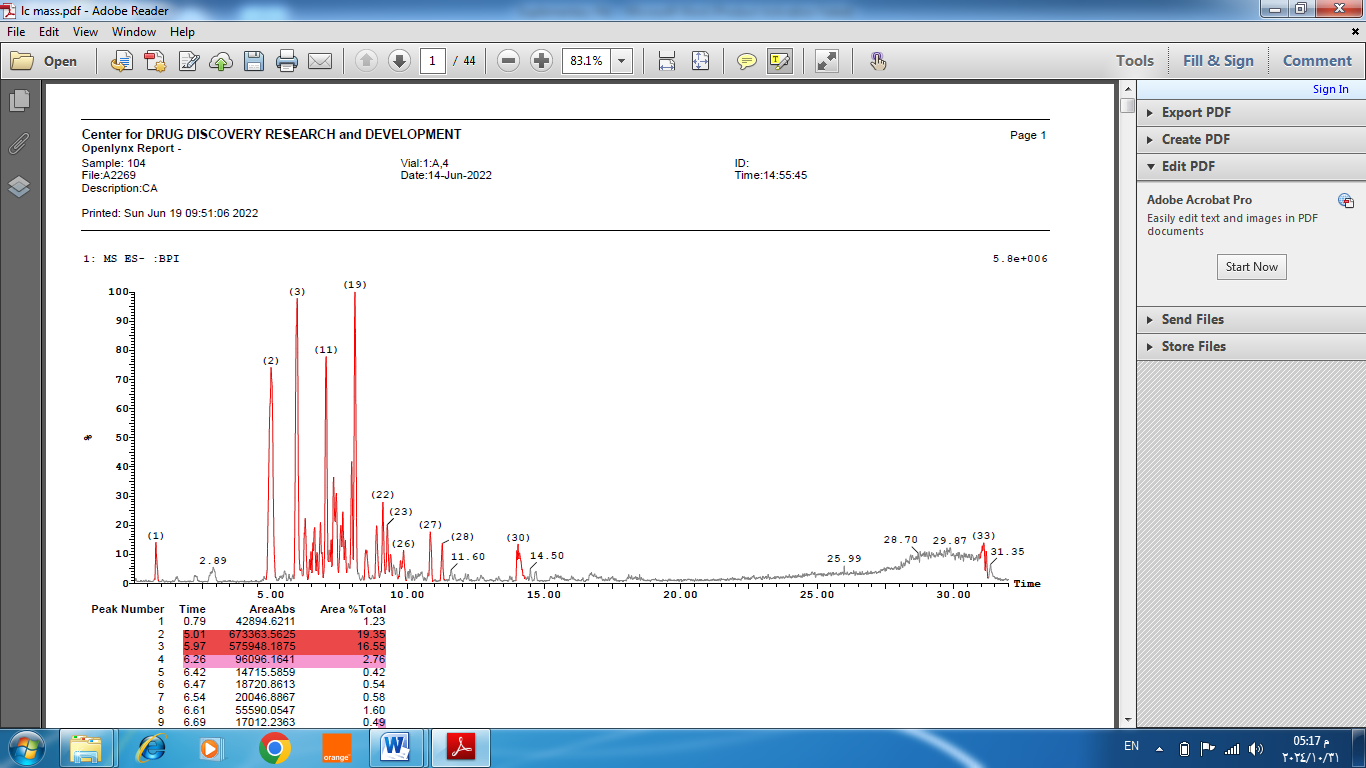


**S-9**

**
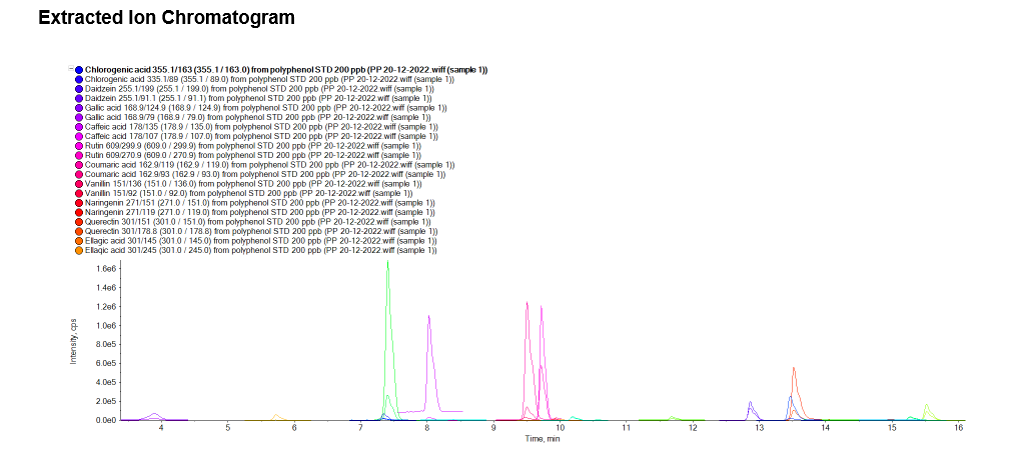
**

**S-10**


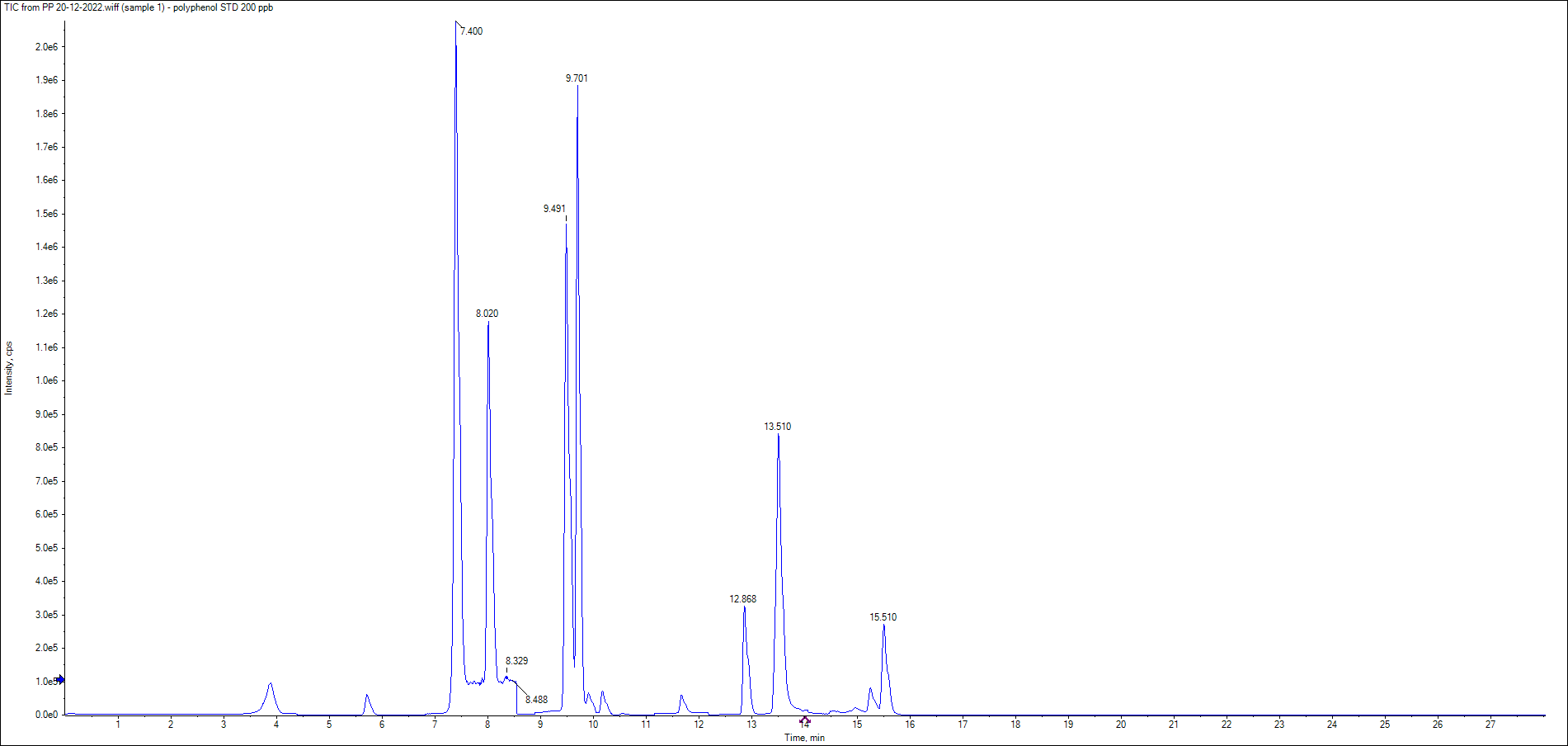


**S-11**


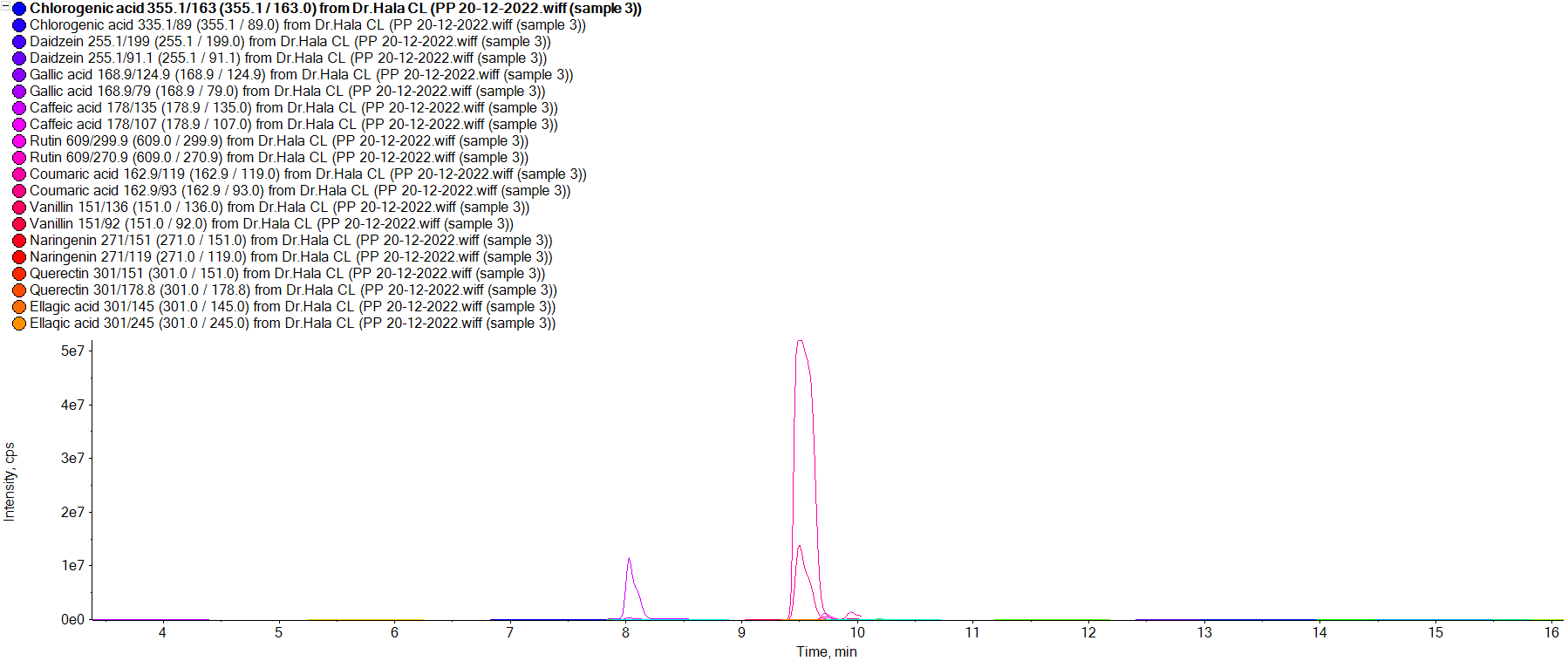


**S-12**


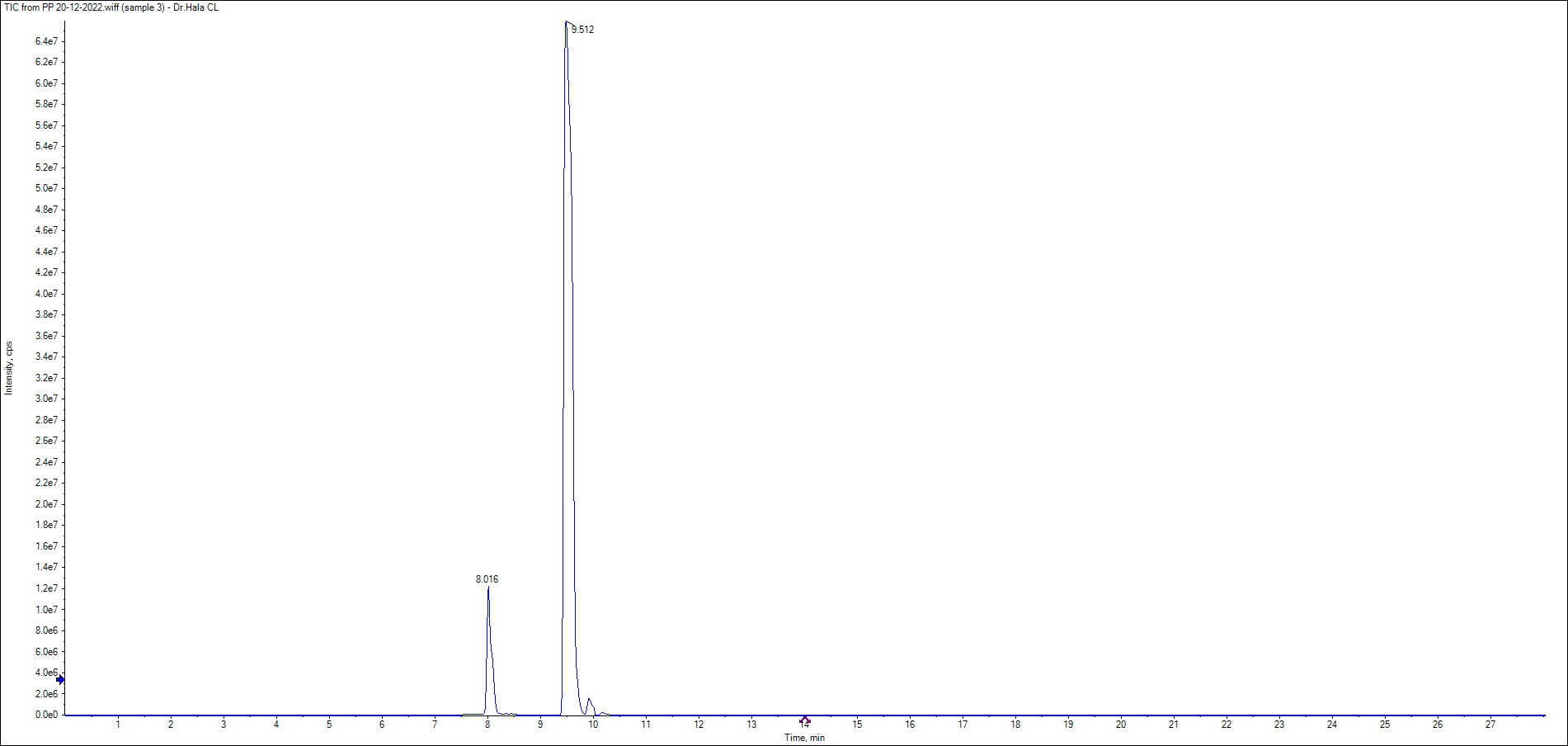


**S-13.** Docking scores and receptors amino acids involved in the interactions with the ligand compounds

| **Ligands** | ***Tg*ROP18**  **(4JRN)** | ***Tg*ROP5**  **(3Q5Z)** | ***Tg*CDPK1**  **4M84** | **UPRTase 1UPF** | **1LIJ** | **3MB8** | **4KY4** | **2O2S** | **6A88** | **4DH4** |
| --- | --- | --- | --- | --- | --- | --- | --- | --- | --- | --- |
|  | **Binding Free Energy ∆G(kcal/mol)/RMSD (Å)**  **Receptor AA Involved (Distance (Å))** | | | | | | | | |  |
| **Spi** | -10.18/*2.07*  **Gly259^b^** (3.22) | -9.67/*3.37*  Asp407^b^ (2.86 & 3.28) | -10.21/*3.11*  Glu178^a^ (2.72) Lys59^a^ (2.98) **Glu135^a,b^** (3.20 & 3.26) Lys338^b^ (3.49) | -9.18/1.70  Arg226^b^ (3.39 & 2.86)  **Tyr 228^d^** (4.58) | -5.46/*3.12*  Asn223^a^ (2.91) Glu135^a^ (3.38) **Thr278^b^** (2.75) | -6.38/*3.46*  Glu35^a^ (2.89) | -8.96/*2.18*  **Cys489^a^** (3.22) **Ser511^b^** (2.89) | -10.41/*2.62*  Ala129^b^ (3.05) **Tyr23^b^** (2.78) | -7.84/*2.80*  Thr592^b^ (2.88) **Thr482^b^** (3.19) **His560^b^** (2.82) | -6.97/*3.30*  Lys102^b^ (3.02) Lys2^b^ (3.01) |
| **1** | -4.22/1.09  **Lys281^b^** (3.11) | -4.38/*2.21*  Asp343^a^ (2.91) | -4.55/1.10  **Glu129^a^** (3.11) | -4.20/0.83  **Gly234^a^** (2.91) **Ile229^b^** (3.48) | -4.66/0.97  **Arg136^b^** (3.14) | -4.51/1.48  **Asp210^a^** (3.00) | -4.12/0.88  **Asn521^a^** (3.43) **Asp513^b^** (2.97 & 3.19) | -4.20/1.63 | -4.75/1.52  **Arg481^b^** (3.00) | -3.96/1.50  **Met109^a^** (4.17) |
| **2** | -4.98/1.24  **Met356^a^** (3.77) **Ala359^b^** (3.11) | -5.45/1.35  Asp343^a^ (3.14) Ala340^b^ (3.32) | -5.13/1.28  **Met112^a^** (3.20) **Tyr131^b^** (3.08) | -4.57/1.83  Arg226^b^ (3.20) **Tyr228^d^** (4.66) | -5.33/1.43  **Asp24^a^** (2.84) **Asn314**^b^ (3.21) **Gly69^b^** (2.89) **Ser70^b^** (3.09) | -5.47/1.02  **Thr96^a^** (2.79) **Met187^b^** (2.82) | -4.59/1.71  Val610^a^ (3.10) **Ala609^b^** (2.95 & 3.06) | -5.53/1.47  **Ala81^b^** (3.11) **Trp43^d^** (3.92) | -3.93/1.47  Lys474^b^ (3.01)  **Arg481^b^** (3.14 & 3.35) **Gln555^b^** (3.01 & 3.41) Arg594^b^ (2.89) | -4.72/1.36  **Pro1^a^** (2.98) Met109^a^ (3.79) Trp107^b^ (2.93) |
| **3** | -6.57/1.90  **Ala359^a^** (2.81) **Met356^a^** (3.20 & 3.28)  Lys365^b^ (2.93)  **2** **Lys281^b^** (3.01) | -7.06/1.86  Asp343^a^ (3.42) **Lys263^b^** (3.38) Asp407^b^ (3.03) Phe396^d^ (4.22) | -7.83/1.69  **Asp195^a^** (3.26) **Tyr131^b^** (3.53) | -6.48/1.78  **Ile229^b^** (3.23) Arg137^b^ (2.97) **Tyr228^d^** (4.13) | -6.56/1.41  **Gly280^a^** (2.77) Glu133^a^ (2.99) | -4.30/1.78  **Arg50^b^** (3.11 & 3.30) | -6.52/1.09  Met608^a^ (4.06) **Ala609^b^** (3.09) | -7.39/1.75  Asp19^a^ (2.89 & 3.15)  Ala18^b^ (3.31) **Trp43^b^** (3.37) **Ala81^b^** (3.22) | -6.30/1.42  **Glu441^a^** (2.96) **Arg470^b^** (2.88) **Gln555^a,b^** (2.95 & 3.16) | -6.08/1.67  **Tyr37^a^** (2.91) Val38^a^ (3.13) |
| **4** | -4.90/1.13  **Asp362^a^** (2.93) Lys365^b^ (2.98) **Ala359^b^** (3.19) **Ala359^d^** (4.39) | -5.03/1.02  Ala339^b^ (3.40) Ala340^b^ (2.93) | -4.93/0.65  **Met112^a^** (3.94) **Tyr131^b^** (3.20) **Val65^d^** (4.07) | -4.60/1.60  Tyr227^a^ (3.01) Met166^a^ (3.80) **Phe236^b^** (3.25) **Tyr228^e^** (3.96) | -5.33/1.56  **Asp24^a^** (2.93, 3.31 & 3.32) | -5.74/1.66  **Thr96^a^** (2.84) | -4.61/1.95  **Ala609^b^** (2.99) | -4.98/0.97  **Leu128^d^** (4.42) | -4.72/1.61  **Glu418^a^** (3.31) **Phe415^e^** (3.94) | -4.41/1.28  **Pro1^a^** (3.14) |
| **5** | -4.82/1.98  **Met356^a^** (3.89) Lys365^b^ (2.99 & 3.19)  **Ala359^d^** (4.10) | -4.97/1.19  Leu304^a^ (2.82) **Lys263^b^** (3.11) | -4.86/1.74  **Glu129^a^** (2.93) **Met112^a^** (3.58 & 4.09)  **Val65^d^** (4.07) | -4.64/1.68  **Gly234^a^** (3.26) **Asp235^a^** (2.77) Tyr227^a^ (3.18) Arg226^b^ (3.28) | -6.22/1.36  **Gly280^a^** (3.23) | -5.09/1.05  **Asp210^a^** (2.77) **Met187^a^** (3.51 & 3.65)  **His12^a^** (3.02) | -4.49/0.49  **Ser511^a^** (2.96) | -5.03/1.12  **Asp80^a^** (2.79) Asn130^b^ (2.90) | -4.90/1.18  **Arg481^b^** (2.94) | -4.16/1.59  Val38^a^ (2.97) **Tyr37^a^** (2.88) Trp107^b^ (3.12) |
| **6** | -5.19/1.13  **Asp362^a^** (2.89) **Ala359^b^** (3.09 & 4.64) | -5.35/1.11  Leu304^a^ (2.85) **Lys263^b^** (3.10) | -5.18/1.08  **Glu129^a^** (2.99) **Met112^a^** (3.90) **Tyr131^b^** (3.19) **Val65^d^** (4.05) | -4.70/0.85  **Asp235^a^** (2.87) **Gly234^a^** (2.98) | -6.76/0.76  **Gly280^d^** (3.91) | -5.81/1.96  **Glu188^a^** (2.78) | -4.59/1.54  **Asn521^a^** (3.34) Tyr429^d^ (4.49) | -5.89/1.78  **Asp80^a^** (2.83) **Ala81^b^** (2.93) **Trp43^e^** (3.69) | -5.06/1.47  **Gln555^b^** (2.94) | -4.44/1.62  Glu106^a^ (2.97) Met39^a^ (3.73) Trp107^e^ (3.98) |
| **7** | -5.37/1.03  **Asp362^a^** (2.92) Lys365^b^ (3.02) **Ala359^d^** (4.45) | -5.21/1.224  Asp343^a^ (3.06) **Val248^d^** (4.28) | -5.56/1.00  **Tyr131^b^** (3.07) **Val65^d^** (3.92) | -5.19/1.71  **Gly234^a^** (3.14) Tyr227^a^ (3.19) Arg226^b^ (3.12) | -6.80/1.65 | -4.89/0.62  **Asp210^a^** (2.91 & 3.37) | -4.90/1.91  **Cys489^a^** (3.83 & 3.84)  **Asn521^b^** (3.09) | -5.66/1.24  **Asp80^a^** (2.77) Asn130^b^ (2.83) **Trp43^e^** (3.85) | -5.62/1.05  **Arg481^b^** (3.58) | -4.65/1.73  **Tyr37^a^** (3.41) |
| **8** | -4.72/1.41  **Met356^a^** (3.90) Lys365^b^ (2.99 & 3.24)  **Ala359^d^** (4.05) | -4.90/2.01  Leu304^a^ (2.82) **Lys263^b^** (3.16) | -4.96/1.52  Ala78^a^ (3.32) **Asp195^a^** (3.56) **Met112^a^** (4.33) | -4.39/1.24  Tyr227^a^ (3.29) **Gly234^a^** (3.06) Arg226^b^ (3.21) | -5.93/*2.64*  **Gly280^d^** (3.83) | -5.20/1.75  **Asp210^a^** (2.84) **Met187^b^** (2.80) | -4.53/1.15  Glu381^a^ (2.88) His490^b^ (3.14) **Asn521^b^** (3.22) | -4.70/1.96  **Ala81^b^** (3.12 & 3.15) | -4.62/1.83  Tyr830^a^ (2.77) | -4.25/0.57  Val38^a^ (3.08) **Tyr37^a^** (2.85) Trp107^b^ (3.10) |
| **9** | -4.86/1.31  **Met356^a^** (3.85) **Lys281^b^** (3.10) **Val266^d^** (4.02 & 4.56) | -5.19/1.13  Asp343^a^ (2.75) | -5.32/1.57  **Glu129^a^** (3.07) **Tyr131^b^** (3.28) **Val65^a^** (3.92) | -4.68/1.83  **Gly234^a^** (2.77) **Ile229^b^** (3.08) | -6.40/1.90  **Asn342^b^** (3.41) | -5.60/1.44  **Glu188^a^** (2.65 & 3.23)  **Met187^b^** (2.84) | -4.65/0.72  **Cys489^a^** (3.22) **Ala609^b^** (2.98) | -5.10/1.57  **Ala81^b^** (3.05 & 3.14)  **Leu128^d^** (4.18) | -5.49/1.59  **Arg481^b^** (3.21) **Gln555^d^** (3.39) | -4.23/1.61  **Tyr37^a^** (2.99) |
| **10** | -5.04/1.97  **Met356^a^** (3.91) **Lys281^b^** (3.08) **Val266^d^** (4.01) | -5.28/0.91  Asp407^b^ (3.23) **Val248^d^** (4.33) | -5.73/1.22  **Asp195^a^** (3.39) | -5.02/1.07 | -5.75/0.70  **Asp24^a^** (3.10) **Asp318**^a^ (3.28) **Ser70^b^** (2.90) **Arg136^b^** (3.29) **Gly315^b^** (3.06) | -5.61/1.55  **Asp210^a^** (3.23) **His12^b^** (2.81) Phe165^d^ (3.78) | -4.80/1.18  **Asn521^a^** (3.06) Met608^a^ (3.29) | -5.22/2.00  **Trp43^e^** (3.85) | -4.80/1.39  **Glu441^a^** (2.83) **Arg470^b^** (3.07) | -4.49/1.87  **Pro1^a^** (3.16) |
| **11** | -4.62/0.71  **Met356^a^** (3.96) **Lys281^b^** (3.09) **Val266^d^** (4.05) | -4.81/1.69  Asp407^b^ (3.40) | -5.25/0.96  **Glu129^a^** (3.51) **Tyr131^b^** (3.03) | -4.41/1.72  Asp164^a^ (3.09) **Ile229^b^** (3.37) | -5.26/0.94  **Asp24^a^** (3.00) **Asp318**^a^ (3.35) **Arg136^b^** (3.23) **Gly315^b^** (3.08) | -5.67/1.69  **Met187^a^** (3.84) | -4.57/1.18  **Asp513^b^** (2.80) | -4.95/1.43  **Leu128^d^** (4.10) | -4.57/1.24  **Thr482^a^** (2.88) **Phe485^e^** (3.94) | -4.23/1.27  Trp107^d^ (3.40) |
| **12** | -6.37/0.85  **Met356^a^** (3.14) **Lys281^b^** (3.10) | -7.15/1.03  **Met337^a^** (3.56)  Asp407^b^ (2.98) **Lys263^b^** (3.10 & 3.26) | -7.33/1.75  **Val65^d^** (4.07) | -6.30/1.52  **Gly234^a^** (3.10) | -5.20/1.05  Gln346^a^ (2.96) Ile310^d^ (3.86) | -3.98/1.82  Thr224^a^ (2.98) | -6.59/1.55  **Asn521^a^** (3.17) **Cys489^a^** (3.64 & 4.10)  **Asp513^b^** (3.45) Phe520^d^ (3.72) | -7.86/1.77  **Ala81^b^** (3.10) **Trp43^d^** (4.01) | -6.28/1.12  **Gln555^a^** (3.23) **Arg470^f^** (4.52) | -5.83/1.79  Met109^a^ (3.21 & 3.71)  Trp107^b^ (2.84) |
| **13** | -6.19/1.12  **Asp427^a^** (3.23) **Lys411^b^** (3.09) **Val266^d^** (4.45) | -7.86/1.43  Ala340^a^ (3.20) **Met337^a^** (3.52)  Asp407^b^ (3.33) **Lys263^b^** (2.93 & 3.00) **Val248^d^** (4.11) | -7.85/1.06  **Met112^a^** (3.35) **Tyr131^b^** (3.39) | -6.37/1.58  **Asp235^a^** (2.88) Tyr227^a^ (2.89) Tyr228^d^ (3.68) | -5.41/1.72  His281^a^ (3.08) **Gly280^d^** (3.72) | -4.06/*2.03* | -6.72/1.22  **Cys489^a^** (3.46) **Ala609^a^** (3.12) | -7.37/1.90  **Ala81^b^** (3.29) **Trp43^d^** (4.81) | -6.79/1.42  **Glu441^a^** (2.94) Phe534^d^ (4.11) **His560^f^** (3.75) | -5.86/1.33  Met109^a^ (3.17 & 3.31)  **Tyr37^a^** (3.19) Trp107^b^ (2.73) |
| **14** | -5.74/1.30  **Asp362^a^** (3.12) Leu258^d^ (4.09 & 4.37)  **Ala359^d^** (4.34) | -6.29/1.76  Ser246^a^ (3.24) **Val248^d^** (3.69) | -6.67/1.14  Ala78^a^ (2.95) **Val65^d^** (3.93) | -5.84/1.27  Asp164^a^ (3.06) Tyr228^d^ (4.21) | -5.42/1.48  Gln346^a^ (2.84) **Gly280^d^** (3.74) | -4.17/1.01 | -5.84/1.42  **Asp513^d^** (3.98) | -6.50/1.80  **Trp43^e^** (3.86) | -6.14/1.70  **Arg470^f^** (3.34 & 4.24) **Phe415^e^** (3.62) | -5.36/0.96  Trp107^d^ (3.88) |
| **15** | -4.74/1.18  **Met356^a^** (4.01  & 3.82)  **Ala359^b^** (3.10) **Val266^d^** (4.13) | -5.63/0.98  Ala340^a^ (3.21) **Val248^d^** (4.23) | -5.43/0.87  **Asp195^a^** (3.46) **Val65^d^** (4.19) | -4-89/1.74  **Ile229^b^** (2.86) | -4.72/0.61  Gln346^a^ (2.85) **Gly280^d^** (4.20) | -5.94/0.76  **Glu188^a^** (3.22) **Asp210^a^** (3.49) | -4.94/1.39  Leu516^d^ (4.04) | -5.05/1.01  **Ala81^b^** (3.28) **Leu128^d^** (3.81) | -4.81/1.30  **Phe415^e^** (3.72 & 3.74) | -4.62/1.72  Met109^a^ (3.39) |
| **16** | -4.58/1.68  **Asp362^a^** (2.88) **Met356^a^** (3.43) Lys365^b^ (2.94) **Val266^d^** (4.35) **Ala359^d^** (4.39) | -5.12/1.40  Asp343^a^ (3.07) **Val248^d^** (4.14) | -4.82/.059  **Met112^a^** (3.72 & 3.90) **Tyr131^b^** (3.44) **Val65^d^** (4.26) | -4.36/1.55  **Gly234^a^** (3.12) Arg226^b^ (3.03) | -4.86/0.90  **Asp24^a^** (2.69 & 3.24)  **Asn73^b^** (3.26) | -5.47/1.04  **Asp210^a^** (3.43) **Met187^a^** (3.60) Asp186^d^ (4.06) | -4.35/0.40  **Ser511^a^** (3.19) Asn406^b^ (3.22) | -4.89/1.61  **Ala81^b^** (2.90) **Leu128^d^** (3.61) | -4.79/.075  Cys591^a^ (4.28) Thr592^b^ (3.28) | -4.23/1.56  Met109^a^ (3.94) **Pro1^a^** (3.15) |
| **17** | -5.03/1.36  **Met356^a^** (3.63) **Lys281^b^** (3.24) **Val266^d^** (4.41) **Ala359^d^** (3.93 & 4.06) | -5.40/0.80  **Met337^a^** (3.76) Arg196^b^ (2.92) Leu240^d^ (4.22) **Val248^d^** (4.23) | -5.43/0.53  **Met112^a^** (4.10) **Tyr131^b^** (3.00) **Val65^d^** (4.11 & 4.19) | -4.73/1.25  **Ile229^b^** (3.13) Tyr228^d^ (4.58) | -5.07/1.21  **Thr278^b^** (3.11) | -5.46/1.36  **Asp210^a^** (3.19) | -5.13/1.27 | -5.53/0.53  **Ala81^b^** (2.99) **Leu128^d^** (4.02 & 4.17) Ala129^d^ (4.33) | -5.33/0.75  Pro438^d^ (3.61) **Phe415^e^** (3.74 & 3.95) | -4.40/0.80  Trp107^e^ (3.83) |
| **18** | -6.62/1.42  **Met356^a^** (3.41) **Asp427^b^** (3.34) | -7.33/1.06  **Lys263**^b^ (3.26) **Val248^d^** (4.01) Phe396^d^ (3.77) | -6.58/.096  Lys80^b^ (2.91) **Val65^d^** (3.68 & 4.50) | -6.19/1.23 | -3.98/1.70 | -4.44/1.74 | -6.58/1.61  **Asn521^b^** (3.02)  Trp403^d^ (4.29 & 4.48) | -6.87/1.27  Ala129^d^ (3.57) | -5.91/1.82  **Arg470^f^** (3.71) | -5.41/*2.06*  Met109^d^ (4.28) |
| **19** | -6.66/1.15  **Met356^a^** (3.71) Met357^a^ (3.43) **Ala359^b^** (2.99) **Val266^d^** (3.97, 4.11 & 4.49) | -6.72/1.91  **Met337^a^** (3.82) **Val248^d^** (3.62) Phe396^d^ (3.64) | -6.51/1.84  **Leu57^d^** (4.29) **Val65^d^** (4.23 & 4.31)  Leu181^d^ (3.67) | -5.95/0.78 | -4.98/1.41  **Gly280^d^** (3.89) | -4.32/1.69 | -6.13/1.71  **Ala609^b^** (3.21) | -7.31/0.98 | -5.97/1.73  **His560^f^** (4.38) | -5.62/1.84  Met109^d^ (4.25) |
| **20** | -5.27/1.30  **Met356^a^** (3.89) | -5.11/1.16  **Lys263^b^** (3.00) | -5.68/1.53 | -5.31/1.34  **Asp235^b^** (3.44) **Phe236^b^** (3.24) | -4.87/1.07 | -3.65/1.97  Arg14^b^ (3.05) | -5.65/1.19  Phe520^d^ (3.67) | -5.65/1.07  **Trp43^b^** (2.96) | -5.09/1.27  Thr592^b^ (3.18) | -4.43/1.46  **Tyr37^d^** (3.64) |
| **21** | -7.75/1.60  **Gly262^b^** (3.28) | -7.47/1.62  Asp393^a^ (2.89) **Lys263^b^** (2.85) Asp244^b^ (2.92 & 2.94) Leu240^d^ (4.36) Val342^d^ (4.37) | -7.34/1.52  Arg55^f^ (4.26) | -7.69/1.59  Gln136^b^ (3.28) Arg137^b^ (2.96 & 3.45) | -5.45/1.56  His281^b^ (3.46) | -5.40/*2.20* | -8.45/1.79  **Cys489^a^** (3.55, 3.77 & 3.82)  **Asp513^b^** (2.83) **Asn521^b^** (2.98) | -8.64/1.44  **Lys197^b^** (3.00) | -7.64/1.95  **Arg470^b^** (2.73, 2.88 & 3.09) **Gln555^b^** (2.87) **Phe485^e^** (3.95) | -6.16/1.77  Lys102^b^ (3.00 & 3.34) Trp107^d^ (3.83) |
| **22** | -7.69/1.86  **Asp427^a^** (2.93) **Lys281^b^** (3.32) | -7.39/1.55  Asp393^a^ (2.53) **Glu275**^a^ (2.82) **Lys263^b^** (2.80) | -6.92/1.90  Gln393^a^ (3.17) Asp394^a^ (2.69) Glu64^a^ (3.14) | -7.38/1.34  Tyr227^a^ (2.87) Met166^a^ (3.64) Glu139^a^ (3.07) Arg226^b^ (2.81) | -4.48/1.52  His281^b^ (3.20) | -4.69/*2.60* | -8.43/1.60  Glu381^a^ (2.68) | -9.66/1.83  Ala129^a,b^ (2.91, 2.99 & 3.43) | -6.22/1.55  **Glu441^a^** (2.65 & 2.83)  **Arg481^b^** (3.17) Lys474^b^ (2.86) Phe534^d^ (3.15) | -5.78/*2.24*  Trp107^d^ (3.85) |
| **23** | -6.90/0.81  **Gly259^d^** (4.12) | -8.09/1.18  **Met337^a^** (3.21 & 3.86) **Val248**^d^ (3.81) | -7.70/1.37  **Glu135^a^** (3.42) **Val65^d^** (3.93) | -6.74/1.76  Tyr227^d^ (3.67) | -6.64/1.61 | -4.61/1.92 | -7.23/1.35  **Cys489^a^** (3.83) Asn406^b^ (2.98) | -8.02/1.11  **Lys197^b^** (3.44) | -7.11/1.36  **His560^b^** (2.82) **Phe415^e^** (3.68) | -6.04/0.94  Met109^d^ (4.13) Trp107^e^ (3.81) |
| **24** | -6.57/1.16  **Asp427^a^** (3.28) | -7.37/1.25  **Met337^a^** (3.63) **Val248^d^** (4.45) | -7.79/0.91  **Asp195^a^** (3.27) **Tyr131^b^** (3.23) **Leu57^a^** (4.24) **Val65^d^** (3.77) | -6.93/1.38  Met166^a^ (3.94) Arg226^b^ (3.21) | -6.06/1.33 | -4.50/1.93  Thr224^d^ (3.97) | -7.13/1.84  **Cys489^a^** (3.88) Asn406^b^ (3.02) | -7.54/0.93  **Ala81^b^** (2.92) | -7.34/1.66  **Thr439^d^** (3.63) **Phe415^e^** (3.70 & 3.84) | -6.05/1.60  Ala108^d^ (3.93) |
| **25** | -6.79/1.87  **Gly261^d^** (3.96) **Val266^d^** (4.23) | -7.65/1.27  **Val248^d^** (3.81) | -7.59/1.67  **Val65^d^** (4.04) | -6.74/1.78  Tyr227^a^ (3.30) Met166^a^ (3.93) | -6.72/1.48  **Gly280^d^** (3.86) | -4.43/1.61 | -7.05/1.23  Ile402^d^ (4.71) | -8.02/0.95  Ala129^d^ (4.84) **Trp43^e^** (3.77) | -7.75/1.10  Cys591^b^ (3.32) **Phe485^d^** (4.12) | -6.25/1.32  Met109^d^ (4.46) Trp107^e^ (3.64) |
| **26** | -7.01/1.25  **Met356^a^** (3.87) **Gly261^d^** (4.46) | -7.96/1.79  **Val248^d^** (3.85) **Lys263^f^** (3.83 & 4.96) Phe396^d^ (3.65) | -7.76/1.89  **Val65^d^** (3.99) | -7.17/1.17  **Gly234^a^** (3.31) Met166^a^ (4.14) | -5.96/1.81  Gln346^a^ (3.31) Val305^a^ (3.14) | -4.44/1.77 | -7.63/1.56  **Cys489^a^** (3.40) **Asn521^b^** (3.45) | -8.81/1.19  **Leu128^d^** (4.25) | -7.01/1.09  **Phe415^e^** (3.55 & 3.57) | -6.65/1.19  Met109^a,d^ (3.84 & 4.34) Trp107^e^ (3.95) |
| **27** | -5.44/1.85  **Asp427^a^** (3.01) **Met356^a^** (3.57) **Ala359^a^** (3.06) **Val266^d^** (3.73) | -6.24/1.21  Pro338^a^ (3.45) Leu304^a^ (2.73) **Lys263^b^** (3.69) **Val248^d^** (4.54) | -5.97/0.70  **Asp195^a^** (2.76) Glu178^a^ (2.94) **Val65^d^** (3.84) | -5.54/1.10  **Asp235^a^** (2.88) Arg226^b^ (3.14) Tyr228^d^ (4.84) | -6.98/1.28  **Asp24^a^** (3.14) **Asn20^b^** (3.41) Cys127^d^ (4.97) **Tyr169^e^** (3.72) | -3.92/1.79  Thr224^a^ (3.09) | -5.69/1.66  Glu381^a^ (3.13) **Asn521^b^** (2.98) **Asp513^d^** (3.83) | -6.24/1.43  **Ala81^b^** (2.98) **Leu128^d^** (4.11) | -5.72/1.21  **Thr439^a^** (2.76) Trp589^a^ (2.93) **His560^b^** (3.18) | -5.16/1.57  **Tyr37^a^** (2.97) Trp107^d,e^ (3.81 & 4.57) Ala108^d^ (4.33) Met109^d^ (4.52) |
| **28** | -6.40/1.28  Lys365^b^ (2.98)  Leu258^d^ (3.64 & 4.15)  **Val266^d^** (3.89) | -7.23/1.16  **Met337^a^** (3.31) Asp343^a^ (3.41) **Val248^d^** (3.78 & 4.59) | -7.37/1.17  **Asp195^a^** (3.27) **Tyr131^b^** (3.22) **Leu57^d^** (4.33) **Val65^d^** (3.80) | -6.78/1.23 | -6.52/0.91 | -4.39/1.22 | -6.73/1.06  **Asn521^b^** (3.22) | -7.53/1.34  **Trp43^d^** (3.57) | -6.72/1.20  **Arg470^b^** (2.98) **Phe415^e^** (3.69) | -5.69/1.64  Trp107^d^ (4.35) |
| **29** | -6.70/0.74  Met323^a^ (3.73) **Val266^d^** (4.26) | -7.19/1.31  **Lys263^b^** (2.76 & 3.61) | -7.21/0.89  **Val65^d^** (4.00) | -6.50/1.17  Tyr227^a^ (3.29) Met166^a^ (3.88) | -6.13/1.44  **Gly280^d^** (3.96) | -4.51/0.93 | -6.29/1.41  **Asn521^b^** (3.24) Gly517^d^ (4.17) | -7.71/1.87  Gly16^b^ (3.35) **Leu128^d^** (4.47) | -7.00/1.14  **Phe415^e^** (3.54 & 3.64) | -5.67/0.97  Met39^d^ (4.83) Trp107^d^ (4.43) |
| **30** | -6.27/1.66  **Val266^d^** (4.35) | -7.01/1.84  Pro338^a^ (3.45) **Val248^d^** (3.90, 4.11 & 4.52) | -7.12/1.20  **Tyr131^b^** (3.54) **Val65^d^** (3.74 & 4.46)  Leu181^d^ (3.58) | -5.86/1.29  **Ile229^b^** (3.02) | -5.78/0.77  **Gly69^b^** (3.10) **Asn73^b^** (2.95) **Arg136^f^** (4.02) | -3.89/2.00 | -6.64/1.84  **Cys489^a^** (3.93) **Asn521^b^** (2.96) | -6.95/1.66  Thr42^b^ (3.36) **Trp43^b,d^** (3.21 & 4.41) | -6.60/1.64  **Thr439^d^** (4.22) **Phe415^e^** (3.43) | -6.00/1.21  Ala108^d^ (4.61) Trp107^d,e^ (3.86 & 4.59) |
| **31** | -6.88/1.68  **Asn414^a^** (2.86) Lys365^b^ (2.86) **Val266^d^** (4.07) | -7.62/1.49  Ser246^a^ (2.86) Asp393^a^ (3.25 & 3.38) **Lys263^b^** (2.92) **Val248^d^** (4.38) | -7.20/0.56  **Glu135^a^** (2.66 & 2.99) **Met112^a^** (3.14) Val130^b^ (3.37) **Tyr131^b^** (3.11) **Leu57^d^** (4.47) **Val65^d^** (4.10) | -6.83/1.59  Asp164^a^ (3.33) **Gly234^a^** (2.93) Tyr228^d^ (4.62) | -4.92/1.54  Val305^a^ (2.74) **Gly280^d^** (3.90) His281^d^ (4.43) | -4.22/2.10 | -7.17/1.40  Asn406^a^ (2.86) **Ala609^a^** (2.81) Tyr553^a^ (2.96) Met608^a^ (3.70) | -7.88/1.73  Ala129^a,b^ (2.71 & 3.34) Ala231^b^ (3.25) **Trp43^d^** (4.17 & 4.54) | -6.61/1.45  **Gln555^a^** (3.01) **Glu441^a^** (3.17) **Arg470^b^** (3.10) Phe534^d^ (3.86) | -5.43/1.23  Met39^a^ (3.64) Glu106^a^ (3.02)  Lys2^b^ (3.02 & 3.07)  Trp107^d^ (3.47) |
| **32** | -6.45/1.36  **Glu300^a^** (3.00) **Asp427^a^** (2.84) **Ala264^a^** (3.01) **Lys281^b^** (3.09) **Gly259^d^** (3.80) | -7.37/*2.01*  Ala340^a^ (3.15) **Lys263^b^** (2.93 & 2.96) | -7.46/1.79  **Asp195^a^** (3.40) **Val65^d^** (3.71, 4.28 & 4.62) | -6.43/1.19  Val111^a^ (2.89) Tyr227^e^ (3.73) | -4.78/*2.05*  Glu135^a^ (2.97) | -4.68/1.61  Thr224^d^ (3.81) | -7.00/1.32  **Cys489^a^** (3.14) Arg510^b^ (3.21 & 3.37) | -7.45/1.61  Asn152^a^ (2.72) Ala129^a^ (2.77) **Ala81^b^** (3.02) | -6.10/1.80  **Gln555^b^** (3.17) **Arg481^b^** (3.30) Lys796^b^ (3.02) | -6.03/1.03  **Tyr37^a^** (3.04) Ser36^a^ (3.02) Met109^a^ (3.01 & 3.46) |
| **33** | -7.11/1.65  **Asp427^a^** (3.02) **Met356^a^** (3.42) **Lys281^b^** (3.03) **Ala359^b^** (3.14) | -8.66/1.66  Leu304^a^ (2.85) **Met337^a^** (3.51) **Val248^d^** (4.08 & 4.13) | -7.45/1.99  Glu178^a^ (2.88) Lys59^a^ (3.47) | -7.16/1.71  **Asp235^a^** (3.09 & 3.22)  **Ile229^b^** (3.17) | -6.10/1.61  Val305^a^ (3.22) His281^a^ (3.20) Gln346^a^ (2.65) **Gly280^d^** (4.40) | -4.82/1.50  Thr229^a^ (3.00) | -7.56/1.52  **Cys489^a^** (3.68 & 3.65)  Phe520^d^ (3.78) Gly517^d^ (3.65) | -8.60/1.38  **Ala81^b^** (3.23) Gly24^b^ (3.26) **Trp43^d^** (3.68) | -7.88/1.76  Thr592^b^ (2.93) **Phe415^e^** (3.67) | -6.05/1.65  Met109^a^ (3.03 & 3.13)  **Tyr37^a^** (2.77) |
| **34** | -8.58/1.62  **Asp427^a^** (3.00) **Met356^a^** (3.29) **Lys281^b^** (2.95) | -9.24/1.87  Asp393^a^ (2.73) Asp407^a^ (3.23) **Met337^a^** (3.48) Ser341^a^ (3.08) **Lys263^b^** (3.02) Asp244^b^ (3.17) | -8.20/1.26  **Met112^a^** (2.99 & 3.41) Glu178^a^ (3.21) | -7.98/1.99  Val111^a^ (2.72) Ile135^a^ (2.96) **Asp235^a^** (2.78) **Gly234^a^** (2.73)  Lys150^b^ (3.12) | -5.78/1.18  Asn223^a^ (2.65 & 2.90) **Thr278^b^** (3.03) | -5.21/1.99  Thr224^a^ (2.97) | -8.67/1.34  **Ala609^a^** (2.98)  Glu381^a^ (2.81 & 2.91)  Asp342^a^ (3.26) His551^d^ (4.12) | -9.72/1.95  **Ala81^b^** (2.95) **Lys197^b^** (2.88) Ala129^d^ (3.76) | -7.82/1.69  **Glu441^a^** (2.91) **Arg470^b^** (3.08 & 3.16) **Gln555^b^** (2.87) **Arg481^b^** (3.10) Phe534^d^ (3.91) Arg594^f^ (4.15) | -6.89/1.88  Ala108^a^ (2.90) Met109^a^ (3.71)  Trp107^b,d^ (2.98, 3.07 & 3.73) |
| **35** | -5.68/1.03  **Met357^a^** (3.45) **Asp427^a^** (2.82) **Ala264^a^** (3.44) **Val266^d^** (4.15 & 4.22)  **Lys281^d^** (3.65) | -6.50/1.68  **Val248^d^** (4.42) | -6.39/1.98  **Met112^a^** (3.73) **Asp195^a^** (2.69) **Tyr131^b^** (3.47) **Val65^d^** (3.87 & 4.41) | -5.45/1.21  **Gly234^a^** (3.21) Tyr227^a^ (3.00) | -5.41/1.02  Thr313^a^ (3.23) **Asp318**^a^ (3.15) **Gly69^b^** (2.79) **Asn73^b^** (3.11) **Tyr169^d^** (3.99) | -3.92/1.21  Thr224^a^ (3.22) | -5.84/1.68  **Cys489^a^** (3.23) **Ser511^a^** (3.00) **Asn521^b^** (2.99) | -6.20/0.87  Asp19^a^ (2.90) | -5.62/1.48  **Glu441^a^** (2.87) **Arg470^b^** (3.41) **Phe415^e^** (3.69) | -5.28/1.08  **Tyr37^a^** (3.03) Glu106^a^ (2.94) Trp107^d,e^ (3.73 & 4.46) |
| **36** | -8.28/*2.27*  **Ala264^a^** (3.04) **Ala359^a^** (3.11) **Met356^a^** (3.67 & 3.94)  Leu258^d^ (4.12) | -8.70/1.18  **Lys263^b^** (3.14) **Leu279^d^** (4.34) | -8.41/*2.13*  **Leu57^a^** ((2.92 & 3.07) **Glu135^a^** (3.06) Glu178^a^ (2.85) **Asp195^a^** (2.87) **Val65^d^** (3.91 & 4.43) | -8.04/1.86  Val111^a^ (3.09) Thr169^a^ (3.03) Arg226^b^ (3.02) | -4.66/*2.13*  Gly42^a^ (3.21) | -5.17/1.95 | -8.37/1.96  **Cys489^a^** (3.03) **Asp513^a^** (2.70) Glu381^a^ (2.73) Arg510^b^ (3.36) **Asn521^d^** (3.97) | -10.20/1.18  Asp19^a^ (3.01) **Lys197^b^** (3.00) **Tyr189^d^** (4.15 & 4.26) | -8.36/1.91  **Glu441^a^** (2.84 & 3.20) **Arg470^b^** (3.47) **Arg481^b^** (3.24) | -6.31/1.92  Met109^a^ (3.08 & 3.67)  Ala108^a^ (3.21) Lys2^b^ (3.16) |
| **37** | -5.81/1.31  **Met356^a^** (3.33 & 3.54)  **Ala359^a^** (2.85) | -6.73/1.55  **Val248^d^** (3.97) | -6.64/0.66  Ala78^a^ (2.84) **Tyr131^b^** (3.22) | -6.00/1.45  **Asp235^a^** (3.09) | -5.95/0.94 | -4.00/*2.04* | -6.33/0.89  Leu516^a^ (2.99) **Asn521^b^** (3.14) | -6.72/1.71  Ala18^b^ (3.25) **Trp43^d,e^** (3.64 & 3.95) | -5.92/0.89  **Glu441^a^** (2.75) | -5.25/1.61  Trp107^b^ (3.22) Ala108^d^ (4.45) |
| **38** | -5.46/1.80  **Met356^a^** (3.38) **Val266^d^** (4.06) | -6.18/1.30  **Met337^a^** (3.79) Phe396^d^ (3.63) | -5.97/1.58  **Glu135^a^** (3.14) | -5.22/1.66  **Gly234^a^** (3.22) | -5.53/1.38  **Gly69^b^** (2.97) **Asn73^b^** (2.99) **Arg136^f^** (4.08) | -3.21/1.72  Thr224^a^ (3.00) | -5.71/0.65  Glu381^a^ (2.94) **Asn521^b^** (3.01) **Asp513^d^** (3.71) | -6.12/1.24  **Leu128^d^** (4.11) | -5.37/1.41  **Arg470^b^** (3.08) | -4.87/1.09  Trp107^d^ (4.17) |
| **39** | -8.32/*2.08*  Ala413^a^ (2.84) **Asp427^a^** (3.40) Leu258^d^ (3.59) **Val266^d^** (3.59) | -8.47/1.49  Asp407^a^ (2.93) **Met337^a^** (3.48) **Arg245^b^** (3.00) **Val248^d^** (3.72) | -8.75/1.26  **Leu57^a^** (3.25) Glu178^a^ (2.77 & 3.03)  Lys59^a^ (3.16) **Asp195^a^** (3.39) Lys338^b^ (2.80) **Tyr131^b^** (3.12) | -7.93/*2.07*  Glu139^a^ (3.08) **Gly234^a^** (2.85) Gln136^b^ (3.42) | -5.81/1.88  **Asn342^a^** (2.74) Asn223^a^ (3.18) Gln346^a^ (3.37) | -5.17/*2.27*  Thr224^a^ (2.92) | -8.66/1.22  **Ser511^a^** (3.21) **Cys489^a^** (3.64) **Asp513^b^** (2.96) | -9.83/1.59  Asp19^a^ (3.24) **Tyr23^b^** (3.34) **Lys197^b^** (2.89) | -7.80/1.26  Gly590^a^ (2.82) **Glu441^a^** (3.15) **His560^b^** (3.35) Phe534^d^ (3.66) | -6.51/1.99  Met39^a^ (3.68) Glu106^a^ (2.84) Val38^a^ (3.27) Ala108^b^ (3.32)  Trp107^d^ (3.86, 4.18 & 4.68) Met109^a,d^ (4.02 & 4.10) |
| **40** | -7.16/1.38  Asp362^a^ (2.96) **Ala359^a^** (3.01) **Asn414^a^** (2.98) Lys365^b^ (3.52) **Ser260^b^** (3.29) Lys365^b^ (3.14) | -8.76/1.0  Asp393^a^ (3.16) **Lys263^b^** (3.01) **Val248^d^** (3.94 & 4.59) | -7.93/1.69  **Met112^a^** (3.09 & 3.25) **Tyr131^a^** (2.82) **Asp195^b^** (2.99) **Leu57^d^** (4.35) **Val65^d^** (4.21) | -7.77/1.25  Ile135^a^ (2.82) Arg137^b^ (3.07) | -6.46/1.30  Gln346^a^ (3.03 & 3.22) | -4.50/*2.33* | -7.52/1.06  **Asn521^a^** (3.01) Ile402^a^ (3.33) | -8.41/1.34  Ser127^a^ (2.89) Leu227^b^ (3.13) **Tyr189^b^** (2.76) | -7.44/1.14  **Glu441^a^** (2.80) Trp589^a^ (3.05) **His560^b^** (2.93) **Arg470^b^** (3.37) | -6.43/1.62  Met109^d^ (4.28) |
| **41** | -8.01/1.86  **Met356^a^** (3.07) **Ala359^a^** (3.26) **Gly261^b^** (3.29) | -9.47/1.75  **Met337^a^** (3.07 & 3.32) **Lys263^b^** (3.09) Asp407^a,b^ (3.39 & 2.93) His389^d^ (3.68) | -7.98/1.71  Gln341^a^ (2.80) **Asp195^a^** (2.68) **Met112^a^** (3.60 & 3.92) | -8.08/0.95  Asp164^a^ (2.96) Arg137^b^ (3.66) Tyr228^d^ (4.38) Arg226^f^ (4.76) | -4.05/1.68 | -5.40/1.86 | -7.77/1.58  Glu381^a^ (3.21)  **Asp513^a^** (2.59) Glu381^a^ (3.20 & 3.55)  Met608^a^ (4.39) Tyr429^b^ (2.99) Met608^b^ (3.31) **Ala609^b^** (3.27) | -9.82/1.13  Leu227^a^ (2.84) Asp19 ^a^ (2.79) Ala231^b^ (3.27) **Lys197^b^** (2.98) | -7.98/1.59  **Gln555^a^** (2.86) Cys591^a^ (3.88) **Arg470^b^** (2.82 & 2.97) | -6.26/1.43  **Tyr37^a^** (3.45) Met39^a^ (3.84) Ala108^b^ (3.04) |

^a^ H-donor, ^b^ H-acceptor, ^c^ Ionic, ^d^ pi-H, ^e^ pi-pi, ^f^ pi-cation

**Bold:** Key amino acid residues at the active pocket

**S-14.** Pharmacokinetics predictions of all compounds computed by SwissADME and PreADMET

| **Comps** | **HBA** | **HBD** | **n-rotb** | **TPSA**  **(Å^2^)** | **Log *P_o/w_*** | **Log *s*** | **Ro5** | **HIA**  **%** | **P_MDCK_** | **P_Skin_** | **P_Caco2_** |
| --- | --- | --- | --- | --- | --- | --- | --- | --- | --- | --- | --- |
|  |  |  |  |  |  |  |  |  | **nm/s** | | |
| **Spi** | 16 | 4 | 11 | 195.38 | 1.98 | -4.30 | 3 | 82.17 | 0.04 | -2.89 | 39.97 |
| **1** | 2 | 1 | 2 | 37.30 | 0.23 | -1.70 | 0 | 94.27 | 51.49 | -2.02 | 20.72 |
| **2** | 7 | 4 | 5 | 132.13 | -0.79 | -0.47 | 0 | 11.89 | 0.58 | -4.28 | 9.20 |
| **3** | 10 | 7 | 13 | 176.42 | -4.78 | -1.00 | 0 | 3.93 | 0.49 | -5.19 | 20.48 |
| **4** | 3 | 1 | 2 | 46.53 | 1.10 | -1.52 | 0 | 93.05 | 78.43 | -2.06 | 19.59 |
| **5** | 5 | 4 | 1 | 98.00 | 0.64 | -1.22 | 0 | 53.70 | 9.54 | -3.63 | 13.85 |
| **6** | 5 | 3 | 2 | 86.99 | 1.03 | -1.23 | 0 | 69.75 | 15.61 | -3.40 | 18.32 |
| **7** | 5 | 2 | 3 | 75.99 | 1.21 | -1.97 | 0 | 82.03 | 29.70 | -2.31 | 18.83 |
| **8** | 4 | 3 | 1 | 77.76 | 1.04 | -1.40 | 0 | 74.75 | 23.70 | -3.31 | 18.30 |
| **9** | 4 | 3 | 2 | 77.76 | 1.01 | -2.55 | 0 | 82.30 | 109.43 | -2.67 | 21.11 |
| **10** | 4 | 2 | 3 | 66.76 | 1.34 | -2.60 | 0 | 90.60 | 228.56 | -1.87 | 21.12 |
| **11** | 3 | 2 | 2 | 57.53 | 1.44 | -2.57 | 0 | 92.09 | 75.06 | -1.71 | 21.11 |
| **12** | 7 | 4 | 6 | 108.61 | 0.57 | -1.73 | 0 | 73.41 | 70.02 | -4.17 | 11.09 |
| **13** | 8 | 5 | 6 | 128.84 | -0.59 | -1.12 | 0 | 51.75 | 0.49 | -4.44 | 6.63 |
| **14** | 2 | 2 | 5 | 40.46 | 5.08 | -4.42 | 0 | 93.65 | 67.65 | -1.42 | 27.85 |
| **15** | 4 | 1 | 1 | 59.67 | 1.19 | -2.15 | 0 | 93.92 | 67.46 | -3.13 | 0.28 |
| **16** | 4 | 2 | 0 | 70.67 | 0.91 | -2.38 | 0 | 88.20 | 39.72 | -3.97 | 18.58 |
| **17** | 4 | 0 | 1 | 52.58 | 2.38 | -3.39 | 0 | 98.17 | 52.92 | -3.71 | 43.47 |
| **18** | 4 | 0 | 3 | 48.67 | 4.95 | -4.56 | 0 | 97.60 | 35.73 | -1.52 | 43.20 |
| **19** | 4 | 1 | 2 | 59.67 | 4.65 | -3.76 | 0 | 95.61 | 32.87 | -1.45 | 12.94 |
| **20** | 2 | 0 | 0 | 26.30 | 3.29 | -4.62 | 0 | 100 | 146.33 | -1.75 | 39.35 |
| **21** | 11 | 0 | 8 | 144.64 | 3.34 | -4.27 | 2 | 98.55 | 0.04 | -1.71 | 34.89 |
| **22** | 7 | 5 | 3 | 127.45 | 2.29 | -3.99 | 1 | 74.50 | 0.05 | -4.66 | 20.90 |
| **23** | 8 | 0 | 7 | 85.59 | 2.89 | -4.24 | 0 | 99.07 | 0.07 | -3.66 | 54.02 |
| **24** | 7 | 0 | 6 | 76.36 | 3.04 | -4.76 | 0 | 98.89 | 0.21 | -3.56 | 55.25 |
| **25** | 7 | 0 | 6 | 76.36 | 3.03 | -4.29 | 0 | 98.89 | 0.07 | -3.55 | 51.22 |
| **26** | 9 | 1 | 7 | 105.82 | 2.79 | -4.47 | 0 | 96.93 | 0.34 | -3.89 | 38.35 |
| **27** | 4 | 6 | 1 | 111.13 | 2.90 | -3.63 | 0 | 79.43 | 36.52 | -4.28 | 4.54 |
| **28** | 6 | 0 | 5 | 67.13 | 3.53 | -5.21 | 0 | 98.44 | 0.28 | -3.44 | 53.17 |
| **29** | 6 | 0 | 5 | 67.13 | 3.42 | -4.74 | 0 | 98.44 | 0.43 | -3.40 | 53.77 |
| **30** | 6 | 1 | 4 | 78.13 | 3.58 | -4.50 | 0 | 96.49 | 5.85 | -3.40 | 37.52 |
| **31** | 10 | 7 | 3 | 181.05 | 1.23 | -3.78 | 1 | 31.37 | 0.54 | -4.61 | 5.49 |
| **32** | 10 | 7 | 3 | 181.05 | 1.13 | -3.53 | 1 | 31.37 | 0.82 | -4.62 | 6.97 |
| **33** | 11 | 6 | 5 | 179.28 | 1.19 | -3.74 | 2 | 42.15 | 0.32 | -4.59 | 5.02 |
| **34** | 14 | 8 | 6 | 228.97 | 0.76 | -4.32 | 3 | 13.77 | 0.12 | -4.49 | 7.16 |
| **35** | 7 | 5 | 1 | 131.36 | 2.15 | -3.67 | 0 | 63.48 | 13.35 | -4.43 | 3.41 |
| **36** | 16 | 10 | 6 | 269.43 | -0.04 | -3.74 | 3 | 2.86 | 0.33 | -4.67 | 7.91 |
| **37** | 6 | 3 | 2 | 96.22 | 2.49 | -4.14 | 0 | 87.19 | 24.42 | -4.19 | 7.00 |
| **38** | 5 | 3 | 1 | 86.99 | 2.60 | -4.02 | 0 | 87.32 | 44.63 | -4.18 | 10.52 |
| **39** | 14 | 8 | 6 | 225.06 | 0.59 | -4.19 | 3 | 11.75 | 0.05 | -4.54 | 7.89 |
| **40** | 12 | 7 | 5 | 199.51 | 0.56 | -4.10 | 2 | 21.60 | 0.53 | -4.70 | 9.93 |
| **41** | 16 | 9 | 7 | 258.43 | 0.09 | -3.43 | 3 | 5.31 | 0.04 | -4.66 | 10.80 |

*HBA:* Hydrogen bond acceptor ≤10; *HBD:* hydrogen bond donor ≤5; *n-rotb:* no. of rotatable bond ≤10; *TPSA:* topological polar surface area ≤130Å^2^; *Log Po/w:* octanol/water partition coefficient -0.7 – +5.0; *Log s:* aqueous solubility scale Insoluble < -10 < Poorly < -6 < Moderately < -4 < Soluble < -2 < Very Soluble < 0 < Highly Soluble; *Ro5*: *Lipinski* number of violations of Lipinski’s rule of five ´max. is 4`; *HIA:* Human Intestinal Absorption; *P_CaCO2_*: Cellular permeability; *P_MDCK_*: Cell permeability, Maden Darby Canine Kidney; *P_Skin_*: Skin permeability.

**S-15.** Pharmacokinetics predictions of all compounds computed by SwissADME and PreADMET

| **Comps** | **T_1/2_**  **(h)** | **BBB**  **C_brain_/C_blood_** | **P-gp** | **CYP2D6** | **CYP3A4** | **PPB**  **%** | **Ames** | **Carcino test** | | **DLs** |
| --- | --- | --- | --- | --- | --- | --- | --- | --- | --- | --- |
|  |  |  |  |  |  |  |  | **Mouse** | **Rat** |  |
| **Spi** | 0.08 | 0.03 | Non | Yes | Yes | 14.53 | Non-mutagenic | + | - | 1.50 |
| **1** | 0.81 | 1.22 | Non | No | No | 9.35 | Mutagenic | - | + | -1.04 |
| **2** | 0.87 | 0.06 | Non | No | No | 5.23 | Mutagenic | - | + | -0.49 |
| **3** | 0.73 | 0.02 | Non | No | Yes | 14.45 | Mutagenic | - | + | -0.44 |
| **4** | 0.88 | 0.56 | Non | No | Yes | 63.14 | Mutagenic | - | + | -1.24 |
| **5** | 0.95 | 0.35 | Non | No | Yes | 65.38 | Mutagenic | - | + | -0.22 |
| **6** | 0.94 | 0.38 | Non | No | Yes | 88.44 | Mutagenic | - | + | -0.65 |
| **7** | 0.95 | 0.54 | Non | No | No | 69.77 | Mutagenic | - | + | -0.81 |
| **8** | 0.94 | 0.44 | Non | No | Yes | 27.11 | Mutagenic | - | + | 0.23 |
| **9** | 0.92 | 0.50 | Non | No | Yes | 40.29 | Mutagenic | - | + | -0.35 |
| **10** | 0.92 | 0.76 | Non | No | Yes | 50.41 | Mutagenic | - | + | -0.61 |
| **11** | 0.90 | 0.69 | Non | No | No | 63.05 | Mutagenic | - | + | -0.81 |
| **12** | 0.83 | 0.07 | Non | No | Yes | 58.57 | Mutagenic | + | - | -0.58 |
| **13** | 0.88 | 0.05 | Non | No | Yes | 39.61 | Mutagenic | - | - | -0.19 |
| **14** | 0.44 | 8.82 | Yes | No | Yes | 100 | Non-mutagenic | + | - | -0.86 |
| **15** | 0.85 | 0.64 | Non | No | No | 29.42 | Mutagenic | - | + | -1.23 |
| **16** | 0.88 | 0.57 | Non | No | Yes | 11.47 | Mutagenic | - | + | -1.22 |
| **17** | 0.46 | 2.07 | Non | No | Yes | 91.66 | Mutagenic | - | + | -0.65 |
| **18** | 0.16 | 1.15 | Non | No | No | 93.49 | Mutagenic | - | + | -1.56 |
| **19** | 0.19 | 1.48 | Yes | No | No | 97.46 | Mutagenic | - | + | -1.47 |
| **20** | 0.18 | 1.24 | Yes | No | No | 100 | Mutagenic | + | + | -0.85 |
| **21** | 0.03 | 0.13 | Yes | No | Yes | 91.02 | Non-mutagenic | + | + | -0.74 |
| **22** | 0.11 | 0.12 | Yes | No | Yes | 73.71 | Non-mutagenic | - | - | 0.18 |
| **23** | 0.37 | 0.03 | Non | No | Yes | 84.86 | Mutagenic | - | + | -0.23 |
| **24** | 0.52 | 0.01 | Yes | No | Yes | 86.04 | Mutagenic | - | + | 0.18 |
| **25** | 0.47 | 0.02 | Yes | No | Yes | 86.24 | Mutagenic | - | + | 0.29 |
| **26** | 0.65 | 0.01 | Non | No | Yes | 76.81 | Mutagenic | - | + | -0.10 |
| **27** | 0.90 | 0.38 | Non | No | Yes | 99.72 | Mutagenic | - | + | 0.38 |
| **28** | 0.46 | 0.01 | Non | No | Yes | 87.39 | Mutagenic | - | + | 0.23 |
| **29** | 0.25 | 0.06 | Yes | No | Yes | 88.11 | Mutagenic | - | + | 0.37 |
| **30** | 0.32 | 0.02 | Non | No | Yes | 87.34 | Mutagenic | - | + | 0.51 |
| **31** | 0.76 | 0.04 | Non | No | Yes | 61.32 | Non-mutagenic | + | - | 0.60 |
| **32** | 0.74 | 0.03 | Non | No | Yes | 61.48 | Mutagenic | + | - | 0.59 |
| **33** | 0.73 | 0.04 | Non | No | Yes | 62.68 | Non-mutagenic | + | - | 0.56 |
| **34** | 0.54 | 0.03 | Non | No | Yes | 52.45 | Non-mutagenic | - | - | 0.85 |
| **35** | 0.93 | 0.17 | Non | No | Yes | 93.24 | Mutagenic | - | + | 0.52 |
| **36** | 0.73 | 0.03 | Non | No | Yes | 43.90 | Non-mutagenic | - | - | 0.91 |
| **37** | 0.76 | 0.22 | Non | No | Yes | 96.79 | Mutagenic | - | + | 0.59 |
| **38** | 0.78 | 0.60 | Non | No | Yes | 100 | Mutagenic | - | + | 0.82 |
| **39** | 0.59 | 0.03 | Non | No | Yes | 51.06 | Non-mutagenic | - | - | 1.05 |
| **40** | 0.91 | 0.03 | Non | No | Yes | 47.83 | Non-mutagenic | - | - | 0.59 |
| **41** | 0.82 | 0.03 | Non | No | Yes | 37.75 | Non-mutagenic | - | - | 0.74 |

*T_1/2_:* Half-life; *CYP2D6 & CYP3A4*: Inhibitor/substrate hepatotoxicity; *Carcino-test*: carcinogenicity positive or negative; *P-gp*: P-glycoprotein substrate; *PPB*: Plasma Protein Binding; C_Brain/Blood_: Penetration of the blood-brain barrier; *DLs*: Drug Likeness Score.


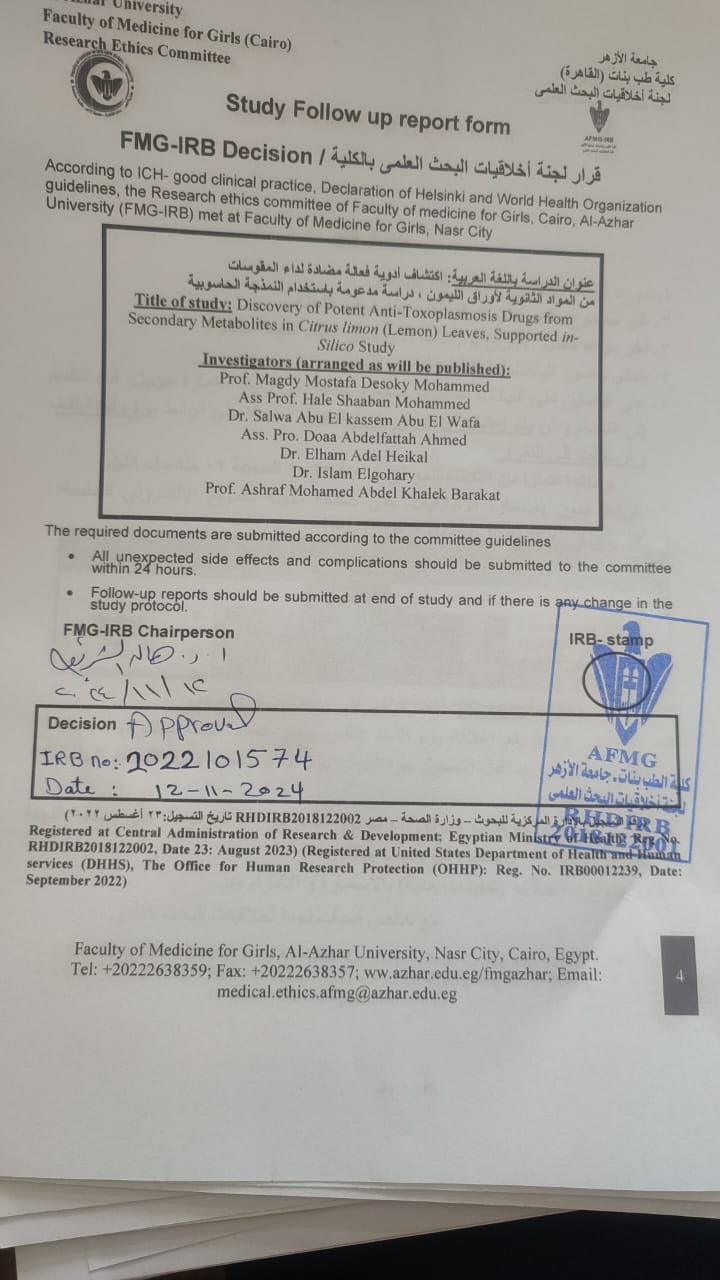

Supplement: Supplementary file 1 — Supplementary Material 1 [file 41598_2024_82787_MOESM1_ESM.docx]
